# The microRNA Expression Profiling in Heart Failure: A Systematic Review and Meta-Analysis

**DOI:** 10.3389/fcvm.2022.856358

**Published:** 2022-06-15

**Authors:** Nan-Nan Shen, Jia-Liang Wang, Yong-ping Fu

**Affiliations:** ^1^Department of Pharmacy, Affiliated Hospital of Shaoxing University, Shaoxing, China; ^2^Department of Cardiology, Affiliated Hospital of Shaoxing University, Shaoxing, China

**Keywords:** miRNAs, heart failure, systematic review, meta-analysis, biomarker

## Abstract

**Background:**

Heart failure (HF) is a main consequence of cardiovascular diseases worldwide. Abnormal expression levels of microRNAs (miRNAs) in HF are observed in current studies. Novel biomarkers miRNAs may play an important role in the development of HF. Nevertheless, the inconsistency of miRNA expression limits the clinical application. We thus perform this systematic review of the miRNAs expression profiling to identify potential HF biomarkers.

**Methods:**

The electronic databases of Embase, Medline, and Cochrane Library were systematically searched to identify the miRNA expression profiles between HF subjects and non-HF controls before May 26th, 2021. The pooled results were shown as log10 odds ratios (logORs) with 95% confidence intervals (CI) using random-effect models. Subgroup analyses were conducted according to species, region, and sample source. The quality assessment of included studies was independently conducted based on Diagnostic Accuracy Study 2 (QUADAS-2). The sensitivity analysis was conducted based on sample size.

**Results:**

A total of 55 miRNA expression articles reporting 276 miRNAs of HF were included. 47 consistently up-regulated and 10 down-regulated miRNAs were identified in the overall analysis, with the most up-regulated miR-21 (logOR 8.02; 95% CI: 6.76–9.27, *P* < 0.001) and the most down-regulated miR-30c (logOR 6.62; 95% CI: 3.04–10.20, *P* < 0.001). The subgroup analysis of sample source identified 35 up-regulated and 10 down-regulated miRNAs in blood sample, the most up-regulated and down-regulated miRNAs were miR-210-3p and miR-30c, respectively. In the region sub-groups, let-7i-5p and miR-129 were most up-regulated and down-regulated in Asian countries, while in non-Asian countries, let-7e-5p and miR-30c were the most dysregulated. It’s worth noting that miR-622 was consistently up-regulated in both Asian and non-Asian countries. Sensitivity analysis showed that 46 out of 58 (79.31%) miRNAs were dysregulated.

**Conclusion:**

A total of 57 consistently dysregulated miRNAs related to HF were confirmed in this study. Seven dysregulated miRNAs (miR-21, miR-30c, miR-210-3p, let-7i-5p, miR-129, let-7e-5p, and miR-622) may be considered as potential non-invasive biomarkers for HF. However, further validation in larger-scale studies are needed to verify our conclusions.

## Introduction

Heart failure (HF), a terminal stage of most cardiovascular diseases, is a major cause of hospitalizations and mortality worldwide (1, 2). The incidence of HF is raising rapidly because of increasing risk factors including hypertension and diabetes, and it is more common in elderly people over 80 years old (3, 4). Currently, the serum brain natriuretic peptide is the only routinely used biomarker for HF recommended in guideline (5), but its clinical value is still uncertain. Therefore, the predictive biomarkers are urgently needed for the early diagnosis of HF. In recent years, a growing number of researches have reported transcriptomic changes are associated with the pathophysiological mechanism of HF (6). Circulating microRNAs (miRNAs) gained significant interest as potential novel HF biomarkers because of high stability, sequence conservation, and non-invasive detection (7). However, prognostic value of microRNAs in HF is still unclear. Thus, the systematic review of miRNAs may contribute to identifying biomarker for early clinical diagnosis of HF.

MiRNAs are a class of conserved non-coding RNAs that regulate protein synthesis on the post-transcriptional level (8). MiRNAs are involved in the pathophysiology of various diseases (9–14), and the aberrant expression of miRNAs were potential biomarker for cardiovascular diseases (15, 16). Increasing evidence has demonstrated dysregulated miRNAs could alter the cellular response of cardiomyocytes, leading to cardiac concentric hypertrophy (17, 18). For example, miR-127 aggravates myocardial failure by regulating the expression of TGF-β1/Smad3 signaling pathway (19), miR-129-5p improves cardiac function in rats with chronic HF through targeting HMGB1 (20), and upregulated miR-132 improved the cardiac dysfunction by inhibiting PTEN expression (21). In general, understanding the pathophysiologic mechanisms in HF could provide the chance to develop new therapies (22, 23).

In previous studies, numerous studies have verified that the circulating miRNAs could be serve as potential biomarkers. For example, miRNA-208 and miRNA-150 have been identified as potential biomarkers for cardiac hypertrophy and remodeling (24, 25), miRNA-21 may serve as a biomarker for coronary heart diseases in the elderly patients (26). MiRNAs change in certain diseases, especially circulating miRNAs due to their stability makes them possible biomarkers in HF (27). Previous studies have reported that the dysregulated miRNAs can serve as the potential diagnostic biomarker in HF (16, 28, 29). Additionally, miRNA-19b, miR-21, miR-423-5p, and miR-92b-5p were observed to be promising biomarker for HF (16, 29–31). Given the pathogenic role of miRNAs in HF, the present study explored an association between miRNAs expression signatures and HF.

Over the past years, the dysregulated miRNAs were investigated in multiple studies, which come from different pathophysiological process, sample size, and inconsistent inclusion criteria, and the expression profiles of miRNAs in HF remain elusive (32, 33). Therefore, we aim to gather the currently available data about dysregulated miRNAs in HF, and to explore promising biomarker for diagnosis and prognosis of HF.

## Methods

### Literature Sources and Search Strategy

Two investigators (N.S. and J.W.) independently searched the PubMed, Embase, and Cochrane Library databases to identify relevant miRNA expression profiling studies with English restrictions from inception to May 26th, 2021. The search terms in the title/abstract were used as follows: (microRNA or miR- or miRNA), (HF or heart failure), (expression or profiling or profile). The detailed search strategies are shown in Supplementary Table 1. All inconsistency was discussed to reach consensus by the corresponding authors (Y-PF and J-LW).

### Literature Selection

The inclusion criteria were as follows: (1) the original studies that investigated miRNA expression level between HF and non-HF subjects; (2) the obtained miRNAs were derived from blood or tissue specimens; (3) sample sizes were represented for dysregulated miRNAs; (4) the expression level of miRNAs were evaluated by qPCR, real-time PCR, and microarray, etc.; (5) only English-written papers were included. The exclusion criteria were as follows: (1) HF patients combined with other diseases; (2) studies based on cell experiments; (3) the literature were letters, comments, case reports, meta-analyses, editorials, or reviews; (4) lack of sufficient data. For duplicate studies from the same research, the one most similar to the inclusion criteria was involved. Two investigators (N.S. and J.W.) separately assessed all the studies to determine their eligibility, and any discrepancies were resolved with consensus by a third reviewer (Y.F.).

### Data Collection and Quality Assessment

Two researchers (N.S. and J.W.) separately extracted the following data from all eligible articles: the name of the first author, year of publication, study population ethnicity, baseline characteristics of patients, species, miRNA detection methods, sample source, number of specimen, direction, and number of dysregulated miRNAs. The quality of included articles was independently assessed using the Quality Assessment of Diagnostic Accuracy Studies-2 (QUADAS-2), which was conducted with eight questions by two investigators (N.S. and J.W.). Any discrepancies were resolved by a third reviewer (Y.F.), and final consensus was achieved through discussion.

### Data Synthesis and Statistical Analysis

All statistical analyses were done using STATA software (version 13; Statacorp, College Station, Texas, United States) by the random-effects model. The results were shown as logORs according to the number of dysregulation between HF and non-HF subjects. The results were shown as logORs according to the number and direction of dysregulation between HF and non-HF subjects. In comparison with those obtained for the non-HF group, logOR values obtained for the HF group higher than 1 revealed upregulation. In comparison with the HF, a marked logOR obtained for the non-HF group higher than 1 revealed downregulation. The *P*-value < 0.05 was considered as statistically different. The importance of ranking of dysregulated miRNAs in HF were based on: (1) number of consistent sub-studies; (2) total sample size; (3) logOR values. Subgroup analysis was performed according to the species, tissue types, and ethnicity. The serum, plasma, or whole blood was classified to blood sample. The sensitivity analysis was performed to explore the heterogeneity. As a crucial determinant for sample size, the sensitivity analysis was repeatedly performed after excluding studies with the sample size of 10 or less.

## Results

### Literature Retrieval, Screening, and Features of Included Articles

**FIGURE 1:**
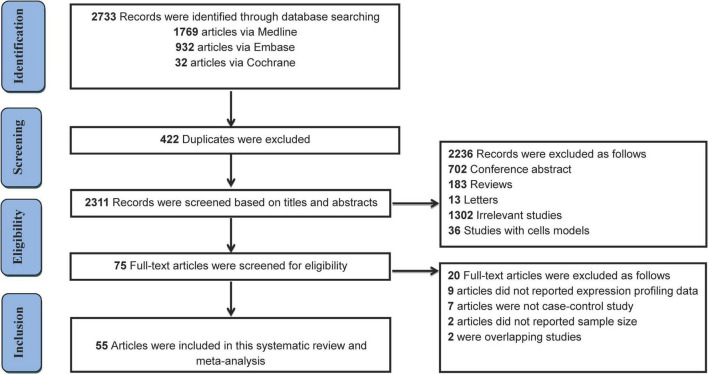
Flow chart for the selection of eligible studies.

The literature selection process is shown in Figure 1. A total of 2,733 studies were initially retrieved from the databases according to the eligibility criteria (Supplementary Table 1). After excluding 422 duplicates, 2,311 studies were further screened based on the eligibility criteria. Ultimately, 55 articles were identified for this meta-analysis (16, 19–21, 26, 28–31, 34–82). The detailed reasons for the excluded studies were shown in Supplementary Table 2. The characteristics of the 55 included studies (41 human and 14 animal articles) were given in Tables 1, 2, respectively. The number of differentially expressed miRNAs in single article ranged from 1 to 69. The patient characteristics of the human studies are given in Supplementary Table 3. The sample sizes varied from 6 to 300 among the studies, and the mean year and the average age ranged 50.4–81.3.

**TABLE 1:** The characteristics of the included human miRNA expression studies.

First author	Country	Sample source	Method	Differentially expressed microRNAs				
				Normalization standards	Sample size Case/Control	Total	Increased	Decreased
Zhang et al. (29)	China	Blood	qRT-PCR	Endogenous U6 rRNA	200/100	1	0	1
Zhang et al. (30)	China	Serum	qRT-PCR	Endogenous U6 rRNA	80/40	1	1	0
Zhang Hao et al. (36)	China	Serum	qRT-PCR	Endogenous U6 rRNA	70/62	1	0	1
Zhang et al. (37)	China	Serum	qRT-PCR	Endogenous U6 rRNA	90/80	1	1	0
Xu and Li (19)	China	Myocardium	qRT-PCR	Endogenous U6 rRNA	51/50	1	1	0
Xiao et al. (20)	China	Serum	qRT-PCR	Endogenous U6 rRNA	83/102	1	0	1
Wu et al. (31)	China	Serum	qRT-PCR	Exosomal miR-451b	43/34	1	1	0
Wong et al. (41)	New Zealand	Plasma	qRT-PCR	Spike-in cel-miR-39	90/90	8	2	6
Watson et al. (28)	Ireland	Serum	qRT-PCR	No reference RNA	75/75	5	0	5
Wang et al. (43)	China	Peripheral blood	qRT-PCR	Endogenous U6 rRNA	31/31	3	3	0
Wahlquist et al. (44)	United States	Heart	qRT-PCR	No reference RNA	5/5	1	1	0
Vogel et al. (45)	Germany	Peripheral blood	miRNA array	Endogenous U6 rRNA	53/39	40	20	20
Tijsen et al. (16)	Netherlands	Plasma	miRNA array	No reference RNA	12/12	13	12	1
Thomé et al. (47)	Brazil	Plasma	qRT-PCR	Spike-in cel-miR-39	20/17	3	3	0
Tao et al. (49)	China	Plasma	qRT-PCR	Spike-in cel-miR-39	34/30	1	1	0
Shirazi-Tehrani et al. (51)	Iran	Serum	qRT-PCR	5s rRNA	20/17	1	0	1
Seeger et al. (52)	Germany	Peripheral blood	qRT-PCR	No reference RNA	20/13	5	0	5
Scrutinio et al. (53)	Italy	Blood	qRT-PCR	No reference RNA	25/15	1	0	1
Schneider et al. (54)	Brazil	Plasma	qRT-PCR	Spike-in cel-miR-39	48/17	3	0	3
Qiang et al. (56)	China	blood	real-time PCR	No reference RNA	51/30	16	11	5
Ovchinnikova et al. (57)	Netherlands	Plasma	qRT-PCR	No reference RNA	20/41	15	15	0
Olivieri et al. (26)	Italy	Plasma	qRT-PCR	Spike-in cel-miR-17	81/99	4	4	0
Melman et al. (58)	Israel	Plasma	qRT-PCR	Endogenous U6 rRNA	6/6	1	1	0
Matsumoto et al. (59)	Japan	Serum	qRT-PCR	Endogenous U6 rRNA	21/65	3	0	3
Marques et al. (60)	Australia	Plasma	real-time PCR	Endogenous U6 rRNA	9/8	21	9	12
Li et al. (63)	China	Plasma	qRT-PCR	No reference RNA	14/10	8	6	2
Lai et al. (65)	China	Heart	real-time PCR	Endogenous U6 rRNA	17/17	7	7	0
He et al. (67)	China	Plasma	qRT-PCR	Endogenous U6 rRNA	8/9	1	1	0
Han et al. (69)	China	Serum	qRT-PCR	Endogenous U6 rRNA	50/30	1	1	0
Guo et al. (70)	China	Blood	qRT-PCR	Spike-in cel-miR-39	94/31	2	2	0
Goren et al. (71)	Israel	Serum	qRT-PCR	Endogenous U6 rRNA	30/30	4	4	0
Gao et al. (72)	China	Plasma	qRT-PCR	Spike-in cel-miR-39	32/16	3	3	0
Galluzzo et al. (73)	Italy	Plasma	real-time PCR	No reference RNA	30/30	32	20	12
Endo et al. (74)	Japan	Plasma	miRNA array	Endogenous U6 rRNA	13/9	11	11	0
Ding et al. (76)	China	Plasma	qRT-PCR	No reference RNA	62/62	6	6	0
D’Alessandra et al. (77)	Italy	Plasma	qRT-PCR	No reference RNA	16/10	12	12	0
Cakmak et al. (78)	Turkey	Serum	miRNA array	No reference RNA	42/15	28	17	11
Ben-Zvi et al. (79)	United States	Serum	qRT-PCR	Spike-in cel-miR-16-5p	39/21	4	4	0
Beg et al. (80)	United States	Plasma	qRT-PCR	Spike-in cel-miR-16	40/20	1	1	0
Abu-Halima et al. (81)	Germany	Blood	qRT-PCR	Endogenous U6 rRNA	40/20	2	0	2
Abu-Halima et al. (82)	Germany	Blood	miRNA array	Endogenous U6 rRNA	3/3	69	35	34

*RT-PCR, reverse transcription-polymerase chain reaction.*

**TABLE 2:** The characteristics of the included animal miRNA expression studies.

First author	Country	Sample source	Differentially expressed microRNAs						
			Method	Normalization standards	Sample size Case/control	Animal model	Total	Increased	Decreased
Zhou et al. (34)	China	Myocardium	Real-time PCR	Endogenous U6 rRNA rRNArrraRNA rRNA	5/5	Rat	1	1	0
Zhang et al. (35)	China	Myocardium	Real-time PCR	No reference RNA	6/6	Mice	1	0	1
Yang et al. (38)	China	Plasma	qRT-PCR	No reference RNA	15/12	Rat	40	33	7
Wong et al. (40)	New Zealand	Plasma	qRT-PCR	No reference RNA	6/13	Sheep	10	10	0
Wang et al. (42)	China	Myocardium	qRT-PCR	Endogenous U6 rRNA	12/12	Mice	29	21	8
Wang et al. (21)	China	Heart	qRT-PCR	Endogenous U6 rRNA	8/8	Rat	1	0	1
Tian et al. (46)	Netherlands	left ventricle	TaqMan microRNA	Endogenous U6 rRNA	6/6	Rat	6	5	1
Su et al. (50)	China	Myocardium	qRT-PCR	Endogenous U6 rRNA	8/8	Rat	1	0	1
Sang et al. (55)	China	Heart	qRT-PCR	No reference RNA	6/6	Rat	1	0	1
Liu et al. (61)	China	Heart	qRT-PCR	Endogenous U6 rRNA	35/15	Rat	4	3	1
Li et al. (62)	China	Heart	qRT-PCR	Endogenous U6 rRNA	5/5	Rat	1	1	0
Jung and Bohan (66)	United States	Plasma	qRT-PCR	No reference RNA	8/9	Dog	8	4	4
Dickinson et al. (68)	United States	Plasma	qRT-PCR	Endogenous U6 rRNA	10/10	Rat	6	6	0
Du et al. (75)	China	Heart	qRT-PCR	Endogenous U6 rRNA	Rat	7	6	1	

*RT-PCR, reverse transcription-polymerase chain reaction.*

### Study Quality

The QUADAS-2 tool was used to assess the quality of all included literature. The detailed context was mentioned in “Methods” section. The results of quality assessment is given in Supplementary Table 4, the included studies satisfied a majority of the items on the QUADAS list. The evaluation results showed that the overall quality of articles was high.

### Differentially Expressed MicroRNAs in Overall Analysis

We included the pooled analysis of 55 studies involving 275 differentially expressed miRNAs in that compared HF subjects with non-HF (Figure 2). Among these miRNAs, 47 were up-regulated and 10 were down-regulated (Supplementary Tables 5, 6). The details of each miRNA are summarized in Supplementary Figures 1–57. According to the results of 10 sub-studies with 709 samples and 3 sub-studies with 173 samples, miR-21 (logOR 8.02; 95% CI: 6.76–9.27, *P* < 0.001) was identified to be the most upregulated miRNA, and miR-30c (logOR 6.62; 95% CI: 3.04–10.20, *P* < 0.001) being the most downregulated one (Figures 3, 4). In addition, 60 miRNAs were identified in at least two articles with inconsistently dysregulated direction. And the inconsistently dysregulated miRNAs in the overall analysis classified by different subgroups are shown in Supplementary Table 7.

**FIGURE 2:**
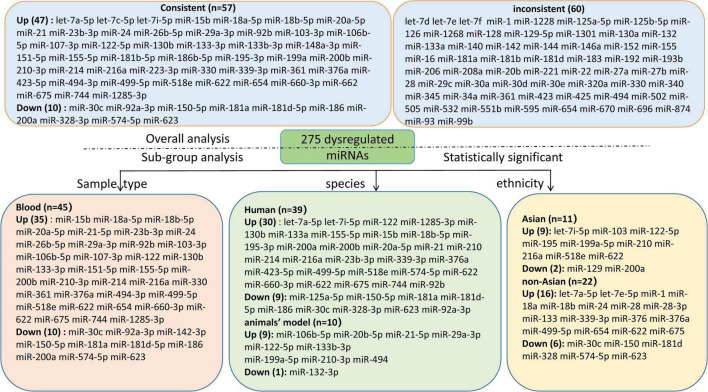
The flow diagram of miRNA categories in this systematic review. miR, microRNA.

**FIGURE 3:**
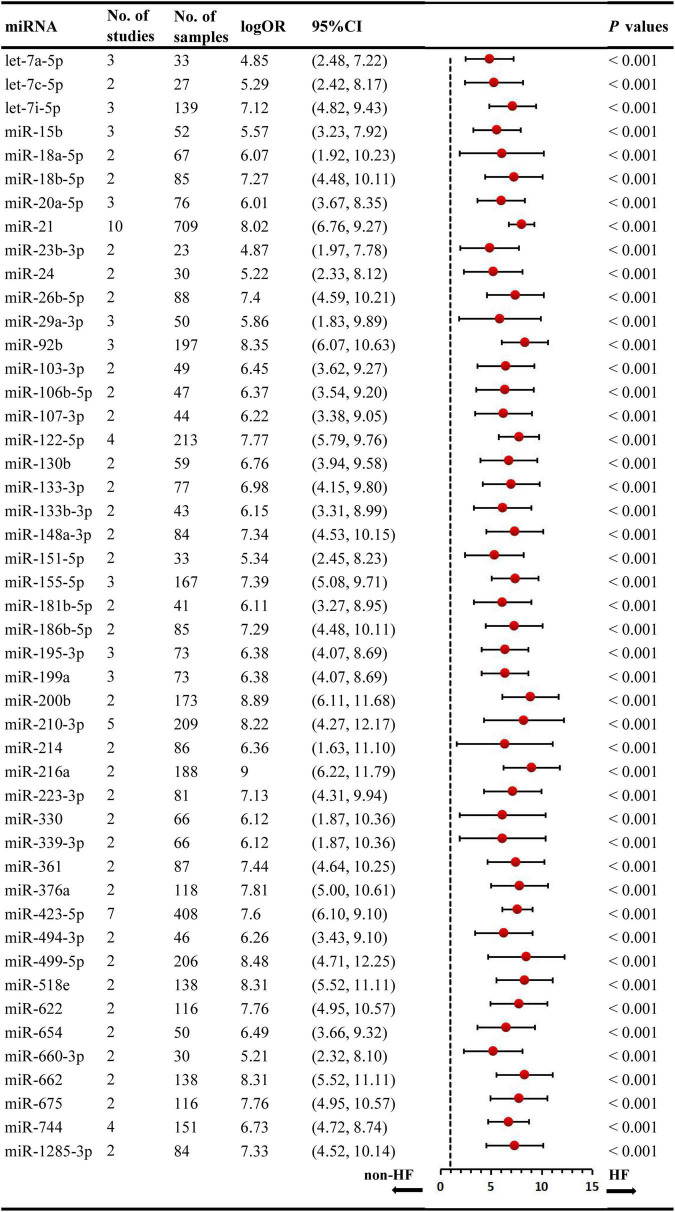
Statistically significant up-regulated miRNAs in overall analysis. miR, microRNA; No, number; HF, heart failure.

**FIGURE 4:**
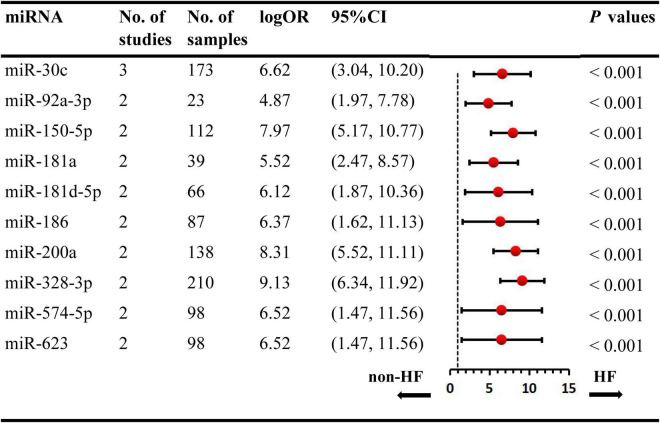
Statistically significant down-regulated miRNAs in overall analysis. miR, microRNA; No, number; HF, heart failure.

### Subgroup Analysis

The subgroup analysis was conducted according to sample source including blood and tissue. 12 studies investigated miRNAs in tissue including myocardium, heart, and left ventricle, 43 studies detected circulating miRNAs in blood source including plasma, peripheral blood, serum, and whole blood. Overall, 45 miRNAs (35 up-regulation and 10 down-regulation) were identified to be aberrantly expressed in blood sample, with the most upregulated and downregulated miRNA being miR-210-3p (logOR 7.16; 95% CI: 5.38–8.94, *P* < 0.001) and miR-30c (logOR 6.62; 95% CI: 3.04–10.20, *P* < 0.001), respectively (Supplementary Table 8). While, no miRNAs were observed to be dysregulated statistically in tissue sample source. The results in the sub-analysis based on serum and plasma were similar to that of the overall analysis (Supplementary Table 9).

The subgroup analyses of ethnicity were conducted based on Asian and non-Asian countries. The subgroup analysis included 33 Asian studies and 22 non-Asian studies, respectively. In the subgroup of Asian countries, there were 9 upregulated and 2 downregulated miRNAs (Supplementary Table 10). For non-Asian subgroup, 16 upregulated miRNAs and 6 downregulated miRNAs were identified (Supplementary Table 11). Remarkably, the expression level of miR-622 was consistently increased in both Asian and non-Asian studies. The expression signature of miRNAs is region-specific. For example, let-7i-5p was significantly upregulated, and miR-129 was downregulated in Asian countries. While, in non-Asian countries, let-7e-5p was significantly upregulated, and miR-30c was downregulated.

Among 55 miRNA expression profiling articles in HF, 41 were human studies, and 14 were from animal articles. There were differences in the expression level of miRNAs between animals and humans, and the species subgroups of the miRNAs were analyzed in Supplementary Tables 12, 13. In the human miRNA studies, 30 significantly upregulated and 9 downregulated miRNAs were identified, with the most upregulated miR-21 (logOR 8.02; 95% CI: 6.76–9.27, *P* < 0.001) and with miR-328-3p (logOR 9.13; 95% CI: 6.34–11.92, *P* < 0.001) being the most downregulated. Among the animal’s studies, several miRNAs were consistently upregulated in two studies, including miR-106a, miR-20b-5p, miR-21-5p, miR-29a-3p, miR-122-5p, etc. But only one miRNA, miR-132-3p, was significantly downregulated in animal research.

### Sensitivity Analysis

The small sample size might influence the robustness of this systematic review, which was determined by sensitivity analysis. We systematically removed 1 study with sample size less than 10, the remaining 54 studies were reanalyzed. As a result, 46 miRNAs were identified significant, with 42 of them upregulated and 4 downregulated (Supplementary Table 14). Finally, the results of sensitivity analysis confirmed that the other 11 miRNAs were not significant, but significant in the overall analysis. The above results showed that a small sample size may lead to differences in miRNA expression profiling.

## Discussion

Over the past years, miRNAs have be observed to play key roles in the physiopathology of HF (56, 72), and there is increasing number of articles reporting on the differentially expressed miRNAs in HF (21, 29, 35). The identification of the dysregulated miRNAs may lead to the novel discovery to monitor the progression of HF. Nevertheless, a major problem with miRNA expression profiling was the inconsistency among different studies. Therefore, we reassembled the data and summarized the abnormal expression signatures of multiple miRNAs in HF through comprehensive data analysis. Finally, this systematic review identified 57 aberrantly expressed miRNAs (47 upregulated, and 10 downregulated) in all included studies. Based on further analysis, several miRNAs (miR-21, miR-30c, miR-210-3p, let-7i-5p, miR-129, let-7e-5p, and miR-622) were identified as potential biomarkers of HF.

The dysregulation of miRNAs, their target genes, are related to signaling pathways of HF, including cardiac hypertrophy, oxidative stress, cardiac fibrosis, etc. (30, 34, 35). The upregulated let-7a regulates β_1_-adrenoceptors (AR) signaling pathway by suppressing β_1_-AR expression in ischemia HF (75). The target genes of the top dysregulation miRNAs identified in this systematic review are all associated with HF. MiR-21 induces fibrotic process by regulating TGFβ1-Smad3 signaling (83), and the inhibition of miR-21 by specific antagomiR leads to reduced hypertrophy and fibrosis (84). Hypoxia-inducible factor-1α (HIF-1α), the target of miR-221-3p inhibits angiogenesis in HF (62). Additionally, miR-122-5p and miR-184 were proved to promote apoptosis in post-infarction HF (61). MiR-150 regulates apoptosis signaling by targeting pro-apoptotic genes and suppressing p53 activity as a major inducer of apoptosis (85).

To date, the early diagnosis of HF is still challenging, a more novel and advanced biomarker are urgently needed. According to the previous studies, miRNAs can be used as a new method for monitoring of HF. The dysregulated miRNAs (miR-19b, miR-129-5p, miR-221-3p, miR-208a, etc.) may provide non-invasive biomarkers for the diagnosis and prognosis of HF (29, 36, 62, 64). Therefore, the practicable detection methods are essential to ensure the accuracy and precision of miRNAs (86). Currently, there are the increasing concern about normalization strategies, that may contribute to the inconsistent expression data of microRNAs in PCR detection (87). The appropriate internal controls in PCR technology were required for the quantification of miRNAs, and relevant studies have verified that the spiked exogenous *C. elegans* miRNAs (e.g., miR-17, miR-454, cel-miR-39, and RNU6) as assay-normalization controls, did not significantly alter assay imprecision (16, 87–90). In PCR-based microRNAs detecting, the findings were robust irrespective of the way of standardization (91). Therefore, the findings in this meta-analysis were relatively reliable. However, in the future study, the rigorous experimental verification for confirming their validity with HF is necessary.

It was investigated whether miRNAs could serve as a promising biomarker of HF. Circulating miRNAs are promising diagnostic biomarkers for HF because of their high sensitivity and specificity. The increasing numbers of studies have focused on the clinical role of miRNAs. However, due to different specimens, sample sizes and regions, the current miRNAs expression characteristics were contradictive. The inconsistent results about dysregulated miRNAs may be caused by a variety of factors, including ethnic differences, the source of samples, and sample sizes. For example, plasma let-7e was upregulated (60, 66), while was downregulated in myocardial tissue (75). MiR-125a-5p was observed to be significantly downregulated (73), but upregulated in animal sample (40). Meanwhile, effects of concomitant treatment on miRNA expression in cardiovascular diseases should not be ignored. Actually, concomitant pharmacological treatment may have an important influence on circulating miRNA signatures. As reported in previous studies, significant changes in circulating miRNAs were observed in response to antiplatelet therapy (92, 93), and antidiabetic treatment increased the levels of let-7a and let-7f (94). The pharmacological treatment should be addressed and provided in the miRNA studies. Regrettably, among our included studies, few studies reported concomitant drugs, and little is known about the influence of drug treatment on the miRNA modulation. Therefore, in future studies, concomitant treatment should be taken into account when using miRNAs as diagnostic biomarker for cardiovascular diseases.

In the present systematic review, subgroup analysis was performed to explore probable sources of heterogeneity. The findings suggest that blood sample may be more informative about HF indicator. 45 dysregulated miRNAs were identified, the most down-regulated miRNAs was miR-30c, and the most upregulated was miR-210-3p. Because the circulating miRNAs are relatively stable and easily detectable by non-invasive methodology (95), circulating miR-30c and miR-210-3p, may be ideal candidate biomarkers for HF. Besides, in Asian countries, let-7i-5p and miR-195 were the most upregulated miRNAs, and miR-129 was the most downregulated one. On the contrary, in non-Asian countries, the significantly upregulated and downregulated miRNAs were let-7e-5p and miR-30c, respectively. Further analysis reveals that more miRNAs are dysregulated in humans than in animals, including miR-21, miR-423-5p, and miR-328-3p. These miRNAs may be the most suitable non-invasive biomarkers in the human’s blood for diagnosing and monitoring of HF.

To the best of our knowledge, the present meta-analysis was the comprehensive and credible to evaluate the miRNA expression signatures in HF. We combined the data of 55 articles to enhance the statistical power and the reliability of the results. However, there are still some limitations. Firstly, individual studies included relatively few patients, which limiting the strength of the conclusions. We thus conducted the sensitivity analysis to guarantee the robustness of the results. Secondly, although a comprehensive literature search was applied, some valuable research may be missing. Additionally, the biological characteristics and mechanisms of the different miRNAs in HF may differ, which may limit the applicability of the pooled results. We thus carried out subgroup analysis to increase the validity and reliability of miRNA analysis for HF.

## Conclusion

This systematic review on miRNA expression profiling studies confirmed several valuable miRNAs, including miR-21, miR-30c, miR-210-3p, let-7i-5p, miR-129, let-7e-5p, and miR-622. These miRNAs may be used as potential non-invasive biomarkers and drug targets for HF. In the future, further investigations are required to verify the value of miRNAs in diagnostic and therapeutic approaches in HF.

## Data Availability Statement

The original contributions presented in the study are included in the article/Supplementary Material, further inquiries can be directed to the corresponding author/s.

## Author Contributions

Y-PF was the guarantors of the entire manuscript. Y-PF and N-NS contributed to the study conception and design, critical revision of the manuscript for important intellectual content, and final approval of the version to be published. J-LW contributed to the data acquisition, analysis, and interpretation. All authors contributed to the article and approved the submitted version.

## Conflict of Interest

The authors declare that the research was conducted in the absence of any commercial or financial relationships that could be construed as a potential conflict of interest.

## Publisher’s Note

All claims expressed in this article are solely those of the authors and do not necessarily represent those of their affiliated organizations, or those of the publisher, the editors and the reviewers. Any product that may be evaluated in this article, or claim that may be made by its manufacturer, is not guaranteed or endorsed by the publisher.
