# Supplementary material for: The microRNA Expression Profiling in Heart Failure: A Systematic Review and Meta-Analysis
Source: Front Cardiovasc Med. 2022 Jun 15;9:856358. doi: 10.3389/fcvm.2022.856358 (PMC9240229; doi:10.3389/fcvm.2022.856358)
Supplement: Supplementary file 1 [file Data_Sheet_1.pdf]

SUPPLEMENTAL FILE

Title: The microRNA expression profiling in heart failure: a systematic review and meta-analysis

Contents

Tables

Supplementary Table 1. Search strategy used onMay26th, 2021 6

Supplementary Table 2. Excluded studies with reasons 7

Supplementary Table 3. Patient demographics and clinical characteristics of included studies. 8

Supplementary Table 4. Quality assessment of miRNA studies according to QUADAS 2 10

Supplementary Table 5. Statistically significant up-regulated miRNAs in overall analysis 13

Supplementary Table 6. Statistically significant down-regulated miRNAs in overall analysis 8

Supplementary Table 7.Inconsistently dysregulated miRNAs classified by different subgroups 17

Supplementary Table 8. Statistically significant dysregulated miRNAs in blood 19

Supplementary Table 9. Statistically significant dysregulated miRNAs in plasma and serum 22

Supplementary Table 10. Statistically significant dysregulated miRNAs in Asian countries 24

Supplementary Table 11. Statistically significant dysregulated miRNAs in non-Asian countries 25

Supplementary Table 12. Statistically significant dysregulated miRNAs in human 27

Supplementary Table 13. Statistically significant dysregulated miRNAs in animals’ model 29

Supplementary Table 14. Statistically significant dysregulated miRNAs in sensitivity analysis according to sample size 30

Figures

Supplementary Figure 1. Forest plot of let-7a-5p 33

Supplementary Figure 2. Forest plot of let-7c-5p 34

Supplementary Figure 3.Forest plot of let-7i-5p 35

Supplementary Figure 4. Forest plot of miR-15b 36

Supplementary Figure 5. Forest plot of miR-18a-5p 37

Supplementary Figure 6. Forest plot of miR-18b-5p 38

Supplementary Figure 7. Forest plot of miR-20a-5p 39

Supplementary Figure 8. Forest plot of miR-21 40

Supplementary Figure 9. Forest plot of miR-23b-3p 41

Supplementary Figure 10. Forest plot of miR-24 42

Supplementary Figure 11. Forest plot of miR-26b-5p 43

Supplementary Figure 12. Forest plot of miR-29a-3p 44

Supplementary Figure 13.Forest plot of miR-92b 45

Supplementary Figure 14. Forest plot of miR-103-3p 46

Supplementary Figure 15. Forest plot of miR-106b-5p 47

Supplementary Figure 16. Forest plot of miR-107-3p 48

Supplementary Figure 17. Forest plot of miR-122-5p 49

Supplementary Figure 18. Forest plot of miR-130b 50

Supplementary Figure 19. Forest plot of miR-133-3p 51

Supplementary Figure 20. Forest plot of miR-133b-3p 52

Supplementary Figure 21. Forest plot of miR-148a-3p 53

Supplementary Figure 22. Forest plot of miR-151-5p 54

Supplementary Figure 23. Forest plot of miR-155-5p 55

Supplementary Figure 24. Forest plot of miR-181b-5p 56

Supplementary Figure 25. Forest plot of miR-186b-5p 57

Supplementary Figure 26. Forest plot of miR-195-3p 58

Supplementary Figure 27. Forest plot of miR-199a 59

Supplementary Figure 28. Forest plot of miR-200b 60

Supplementary Figure 29. Forest plot of miR-210-3p 61

Supplementary Figure 30. Forest plot of miR-214 62

Supplementary Figure 31. Forest plot of miR-216a 63

Supplementary Figure 32. Forest plot of miR-223-3p 64

Supplementary Figure 33. Forest plot of miR-330 65

Supplementary Figure 34. Forest plot of miR-339-3p 66

Supplementary Figure 35. Forest plot of miR-361 67

Supplementary Figure 36. Forest plot of miR-376a 68
Supplementary Figure 37. Forest plot of miR-423-5p 69
Supplementary Figure 38. Forest plot of miR-494-3p 70
Supplementary Figure 39. Forest plot of miR-499-5p 71
Supplementary Figure 40. Forest plot of miR-518e 72
Supplementary Figure 41. Forest plot of miR-622 73
Supplementary Figure 42. Forest plot of miR-654 74
Supplementary Figure 43. Forest plot of miR-660-3p 75
Supplementary Figure 44. Forest plot of miR-662 76
Supplementary Figure 45. Forest plot of miR-675 77
Supplementary Figure 46. Forest plot of miR-744 78
Supplementary Figure 47. Forest plot of miR-1285-3p 79
Supplementary Figure 48. Forest plot of miR-30c 80
Supplementary Figure 49. Forest plot of miR-92a-3p 81
Supplementary Figure 50. Forest plot of miR-150-5p 82
Supplementary Figure 51. Forest plot of miR-181a 83
Supplementary Figure 52. Forest plot of miR-181d-5p 84
Supplementary Figure 53. Forest plot of miR-186 85
Supplementary Figure 54. Forest plot of miR-200a 86

Supplementary Figure 55. Forest plot of miR-328-3p 87

Supplementary Figure 56. Forest plot of miR-574-5p 88

Supplementary Figure 57. Forest plot of miR-623 89

References 90

Abbreviations in Supplementary Figures

HF: heart failure; AF: atrial fibrillation; miRNA: microRNAs; NR: not reported; 3'-UTRs: 3'-untranslated regions; qRT-PCR:quantitative real-time polymerase chain reaction; SBP: systolic blood pressure; DBP: diastolic blood pressure; BMI: body mass index; DM: diabetes mellitus; LVEF: left ventricular ejection fraction;Y: Yes; N: No; U: Unclear.

Supplementary Table 1. Search strategy used on May 26th, 2021

**Literature databases**	**Search items**	**Items found**
MEDLINE	(“microRNA”[MeSH Terms] OR “microRNA”[Title/Abstract] OR “miRNA”[Title/Abstract] OR “miR-”[Title/Abstract]) AND[(“heart failure”[Title/Abstract] OR “heart failure”[MeSH Terms] OR “HF”[Title/Abstract]) OR (“heart exhaustion”[Title/Abstract] OR “heart exhaustion”[MeSH Terms]] AND (“expression”[Title/Abstract] OR “profile” [Title/Abstract] OR “profiling”[Title/Abstract])	1769
EMBASE	(‘microRNA’/exp OR ‘microRNA’:ti, ab,kw OR ‘miRNA’: ti,ab,kw OR ‘miR-’: ti,ab,kw) AND (‘heart failure’/exp OR ‘heart failure’:ti, ab,kw OR ‘HF’: ti,ab,kw OR ‘heart exhaustion’/exp OR ‘heart exhaustion’:ti, ab,kw) AND (‘expression’ : ti,ab,kw OR ‘profile’: ti,ab,kw OR ‘profiling’: ti,ab,kw)	932
COCHRANE	(MeSH descriptor: [microRNA] OR microRNA: ti,ab,kw OR miRNA: ti,ab,kw OR miR-: ti,ab,kw) AND (MeSH descriptor: [heart failure] OR heart failure: ti,ab,kw OR heart exhaustion: ti,ab,kw) AND (expression : ti,ab,kw OR profile: ti,ab,kw OR profiling: ti,ab,kw)	32
Overall		2733

Supplementary Table 2. Excluded studies with reasons

**Study**	**Sample type**	**Reason for exclusion**
Wang N 2009(1)	tissue	Not reported sample size
Silva, F 2020(2)	tissue	Not reported sample size
Paseban, M 2020(3)	tissue	Not reportednon-HF control samplesfor comparison
Masson S 2017(4)	tissue	Not reportednon-HF control samplesfor comparison
Maria E 2018(5)	cell	Cell research
Liu, Q 2013(6)	cell	Cell research
Lin X 2019(7)	serum	Not reportednon-HF control samplesfor comparison
Lin B 2019(8)	atrial tissue	Not reportednon-HF control samplesfor comparison
Bauters C 2013(9)	atrial tissue	Not reportednon-HF control samplesfor comparison
Bayés-Genis 2018(10)	serum	Not reportednon-HF control samplesfor comparison
Beaumont J 2017(11)	plasma	Not reportednon-HF control samplesfor comparison
Cai W 2015(12)	cell	Cell research
Chen F 2018(13)	serum	No data
Dawson K 2013(14)	atrialtissue	No data
Schneider S 2018(15)	plasma	No data
Duan Q 2015(16)	serum	No data
Funahashi H 2011(17)	serum	No data
Goldraich L 2014(18)	serum	No data
Gupta M 2013(19)	cell	Cell research
He Y 2020(20)	serum	No data
Hu L 2021(21)	serum	No data
Dubois-Deruy, E 2017(22)	cell	Cell research
Lin, B 2019(8)	serum	No data

Supplementary Table 3. Patient demographics and clinical characteristics of included studies.

Study	Total number	Mean age(y)	Female (%)	Smoking (%)	AF (%)	BMI (kg/m2) (mean)	SBP (mmHg) (mean)	DBP (mmHg) (mean)	Hypertension (%)	DM (%)	NT-pro BNP (pg/mL)	Creatinine (mmol/l) (mean)	LVEF (%)
Zhang et al. 2020	300	62	80	6.0	20.0	NR	NR	NR	48	17	2141.7	NR	59.4
Zhang et al. 2017	120	59.68	28.75	NR	11.25	NR	115.54	71.13	28.75	25.0	NR	79.31	63.07
Zhang Hao et al. 2021	132	67.24	47.14	37.14	NR	24.95	NR	NR	68.57	65.71	1519.83	NR	30.17
Zhang et al. 2019	170	64.31	46.67	18.3	NR	22.91	126.44	NR	10.9	13.9	NR	98.41	NR
Xu et al.2018	101	NR	NR	NR	NR	NR	NR	NR	NR	NR	NR	NR	NR
Xiao et al. 2019	185	61.27	40.2	NR	NR	26.1	NR	NR	NR	NR	1486.9	NR	NR
Wu et al. 2018	77	62	25.6	46.5	NR	24	NR	NR	51.2	46.5	NR	NR	39.1
Wong et al. 2015	180	60.21	NR	NR	NR	26.93	120.02	69.91	81	62.1	3084.57	NR	38.67
Watson et al. 2015	150	75	39	NR	80	31	NR	NR	84	31	NR	NR	NR
Wang et al. 2018	62	61.3	41.94	NR	NR	NR	NR	NR	NR	6.45	1566.8	NR	31.5
Wahlquist et al. 2014	10	NR	NR	NR	NR	NR	NR	NR	NR	NR	NR	NR	NR
Vogel et al. 2013	92	60	16.98	NR	NR	NR	127	77	68	21	2399	133	33
Tijsen et al. 2010	24	68.2	46.7	NR	NR	NR	NR	NR	NR	NR	NR	98.9	37.1
Thom et al. 2015	37	54.9	80.0	NR	NR	21.8	135	80	40	80	NR	NR	NR
Tao et al. 2019	64	58.8	39.06	15.6%	NR	NR	104.6	74.4	15.6	23.4	NR	NR	34.8
Shirazi-Tehrani et al. 2020	37	64.83	45.71	31.4	NR	26.0	NR	NR	NR	37.1	NR	NR	NR
Seeger et al. 2013	33	64	12.5	NR	NR	NR	NR	NR	61.11	38.89	NR	NR	NR
Scrutinio et al. 2017	40	60	20	NR	38	27.6	104	NR	55	14	NR	112.9	21.4
Schneider et al. 2018	65	64	27	73	73	NR	133	87	82	64	NR	114	26
Qiang et al. 2013	81	66.4	23.53	13.7	NR	NR	129.7	63.4	NR	NR	NR	NR	31
Ovchinnikova et al. 2015	61	65.4	16.7	NR	58	NR	119.4	71.3	20.8	20	1153.5	NR	34.1
Olivieri et al. 2013	180	81.3	58	NR	NR	NR	NR	NR	69	48	NR	NR	41

Melman et al. 2015	12	61.7	20	NR	NR	NR	NR	NR	60	33	NR	NR	24.9
Matsumoto et al. 2013	86	NR	NR	NR	NR	NR	NR	NR	NR	NR	NR	NR	NR
Marques et al. 2016	17	50.4	40	NR	NR	29.4	106	60	NR	NR	NR	NR	NR
Li et al. 2016	10	NR	NR	NR	NR	NR	NR	NR	NR	NR	NR	NR	NR
Lai et al. 2015	34	54	17.6	NR	NR	NR	NR	NR	NR	NR	NR	NR	NR
He et al. 2017	17	55.98	30	33.33	NR	NR	NR	NR	NR	12.5	6626.48	98.33	27.55
Han et al. 2020	80	62.8	46	48	NR	23.94	NR	NR	NR	NR	NR	NR	NR
Guo et al. 2018	125	77.4	52.13	NR	NR	22.9	NR	NR	NR	NR	2425	NR	55.2
Goren et al. 2012	60	64.5	17	13	NR	NR	NR	NR	NR	57	NR	NR	30
Gao et al. 2019	48	NR	NR	NR	NR	NR	NR	NR	NR	NR	NR	NR	NR
Galluzzo et al. 2021	60	65	87	NR	NR	23.9	NR	NR	23	23	4406	NR	21
Endo et al. 2013	22	70.7	15.38	NR	NR	23.1	NR	NR	NR	NR	NR	NR	NR
Ding et al. 2020	124	62	60	NR	NR	26.07	NR	NR	NR	NR	1809.6	NR	NR
Alessandra et al. 2020	26	64	21	57	NR	NR	NR	NR	50	29	NR	NR	38.2
Cakmak et al. 2015	57	56.57	28.6	NR	NR	27.97	112.4	72.07	NR	NR	NR	NR	31.67
Ben-Zvi et al. 2020	60	69.5	36	13	23	35	NR	NR	77	44	NR	NR	30
Beg et al. 2017	60	66.5	5	NR	NR	30.4	NR	NR	80	58	NR	NR	NR
Abu-Halima et al. 2019	60	NR	NR	NR	NR	NR	123.2	66.2	NR	NR	NR	NR	55.3
Abu-Halima et al. 2017	6	NR	NR	NR	NR	NR	122.7	70.0	NR	NR	1747.0	NR	33.3

Supplementary Table 4. Quality assessment of miRNA studies according toQUADAS 2.

Studies	Was a consecutive or random sample of patients enrolled?	Did the studyavoidina ppropriate exclusions?	Is the reference standard likely to correctly classify the targetcondition?	Were the referencestandard resultsinterpreted without knowledge of theresults of the indextest?	Was there an appropriate interval between index tests and reference standard?	Was the blind method used in result of referance standard?	Did allpatients receive thesame reference standard?	Were all patients included in the analysis?
Zhou et al.2015	Y	U	Y	Y	Y	Y	Y	Y
Zhang et al. 2021	Y	U	Y	Y	Y	Y	Y	Y
Zhang et al. 2020	N	U	Y	Y	Y	Y	Y	Y
Zhang et al. 2017	Y	N	Y	Y	Y	Y	Y	Y
Zhang Hao et al. 2021	N	N	Y	Y	Y	Y	Y	Y
Zhang et al. 2019	Y	Y	Y	Y	Y	Y	Y	Y
Yang et al. 2020	N	N	Y	Y	Y	Y	Y	Y
Xu et al.2018	N	U	Y	Y	Y	Y	Y	Y
Xiao et al. 2019	U	U	Y	Y	Y	Y	Y	Y
Wu et al. 2018	U	N	Y	Y	Y	Y	Y	Y
Wong et al. 2017	Y	N	Y	Y	Y	Y	Y	Y
Wong et al. 2015	N	N	Y	Y	Y	Y	Y	Y
Watson et al. 2015	U	Y	Y	Y	Y	Y	Y	Y
Wang et al. 2017	U	N	Y	Y	Y	Y	Y	Y
Wang et al. 2018	U	U	Y	Y	Y	Y	Y	Y

Wang et al. 2020	N	U	Y	Y	Y	Y	Y	Y
Wahlquist et al. 2014	N	N	Y	Y	Y	Y	Y	Y
Vogel et al. 2013	U	Y	Y	Y	Y	Y	Y	Y
Tijsen et al. 2010	U	U	Y	Y	Y	Y	Y	Y
Tian et al. 2018	N	U	Y	Y	Y	Y	Y	Y
Thom et al. 2015	N	N	Y	Y	Y	Y	Y	Y
Tao et al. 2019	N	Y	Y	Y	Y	Y	Y	Y
Su et al. 2019	N	Y	Y	Y	Y	Y	Y	Y
Shirazi-Tehrani et al. 2020	U	N	Y	Y	Y	Y	Y	Y
Seeger et al. 2013	U	N	Y	Y	Y	Y	Y	Y
Scrutinio et al. 2017	N	U	Y	Y	Y	Y	Y	Y
Schneider et al. 2018	Y	U	Y	Y	Y	Y	Y	Y
Sang et al. 2015	Y	U	Y	Y	Y	Y	Y	Y
Qiang et al. 2013	N	Y	Y	Y	Y	Y	Y	Y
Ovchinnikova et al. 2015	N	Y	Y	Y	Y	Y	Y	Y
Olivieri et al. 2013	N	Y	Y	Y	Y	Y	Y	Y
Melman et al. 2015	Y	U	Y	Y	Y	Y	Y	Y
Matsumoto et al. 2013	U	N	Y	Y	Y	Y	Y	Y
Marques et al. 2016	N	Y	Y	Y	Y	Y	Y	Y
Liu et al. 2016	N	Y	Y	Y	Y	Y	Y	Y
Li et al. 2016	N	Y	Y	Y	Y	Y	Y	Y
Li et al. 2021	Y	N	Y	Y	Y	Y	Y	Y

Lai et al. 2015	Y	U	Y	Y	Y	Y	Y	Y
Jung et al. 2018	N	U	Y	Y	Y	Y	Y	Y
He et al. 2017	U	N	Y	Y	Y	Y	Y	Y
Dickinson et al. 2013	U	Y	Y	Y	Y	Y	Y	Y
Han et al. 2020	N	Y	Y	Y	Y	Y	Y	Y
Guo et al. 2018	Y	U	Y	Y	Y	Y	Y	Y
Goren et al. 2012	Y	N	Y	Y	Y	Y	Y	Y
Gao et al. 2019	N	Y	Y	Y	Y	Y	Y	Y
Galluzzo et al. 2021	N	Y	Y	Y	Y	Y	Y	Y
Endo et al. 2013	N	Y	Y	Y	Y	Y	Y	Y
Du et al. 2017	Y	N	Y	Y	Y	Y	Y	Y
Ding et al. 2020	U	U	Y	Y	Y	Y	Y	Y
Alessandra et al. 2020	N	U	Y	Y	Y	Y	Y	Y
Cakmak et al. 2015	N	U	Y	Y	Y	Y	Y	Y
Ben-Zvi et al. 2020	Y	N	Y	Y	Y	Y	Y	Y
Beg et al. 2017	N	N	Y	Y	Y	Y	Y	Y
Abu-Halima et al. 2019	U	Y	Y	Y	Y	Y	Y	Y
Abu-Halima et al. 2017	U	N	Y	Y	Y	Y	Y	Y

Supplementary Table 5.Statistically significant up-regulated miRNAs in overall analysis (n=47)

miRNA	No. of studies	No. of samples	logOR	95%CI	*P* values
let-7a-5p	3	33	4.85	(2.48, 7.22)	< 0.001
let-7c-5p	2	27	5.29	(2.42, 8.17)	< 0.001
let-7i-5p	3	139	7.12	(4.82, 9.43)	< 0.001
miR-15b	3	52	5.57	(3.23, 7.92)	< 0.001
miR-18a-5p	2	67	6.07	(1.92, 10.23)	< 0.001
miR-18b-5p	2	85	7.27	(4.48, 10.11)	< 0.001
miR-20a-5p	3	76	6.01	(3.67, 8.35)	< 0.001
miR-21	10	709	8.02	(6.76, 9.27)	< 0.001
miR-23b-3p	2	23	4.87	(1.97, 7.78)	< 0.001
miR-24	2	30	5.22	(2.33, 8.12)	< 0.001
miR-26b-5p	2	88	7.40	(4.59, 10.21)	< 0.001
miR-29a-3p	3	50	5.86	(1.83, 9.89)	< 0.001
miR-92b	3	197	8.35	(6.07, 10.63)	< 0.001
miR-103-3p	2	49	6.45	(3.62, 9.27)	< 0.001
miR-106b-5p	2	47	6.37	(3.54, 9.20)	< 0.001
miR-107-3p	2	44	6.22	(3.38, 9.05)	< 0.001
miR-122-5p	4	213	7.77	(5.79, 9.76)	< 0.001
miR-130b	2	59	6.76	(3.94, 9.58)	< 0.001
miR-133-3p	2	77	6.98	(4.15, 9.80)	< 0.001

miR-133b-3p	2	43	6.15	(3.31, 8.99)	< 0.001
miR-148a-3p	2	84	7.34	(4.53, 10.15)	< 0.001
miR-151-5p	2	33	5.34	(2.45, 8.23)	< 0.001
miR-155-5p	3	167	7.39	(5.08, 9.71)	< 0.001
miR-181b-5p	2	41	6.11	(3.27, 8.95)	< 0.001
miR-186b-5p	2	85	7.29	(4.48, 10.11)	< 0.001
miR-195-3p	3	73	6.38	(4.07, 8.69)	< 0.001
miR-199a	3	73	6.38	(4.07, 8.69)	< 0.001
miR-200b	2	173	8.89	(6.11, 11.68)	< 0.001
miR-210-3p	5	209	8.22	(4.27, 12.17)	< 0.001
miR-214	2	86	6.36	(1.63, 11.10)	< 0.001
miR-216a	2	188	9.00	(6.22, 11.79)	< 0.001
miR-223-3p	2	81	7.13	(4.31, 9.94)	< 0.001
miR-330	2	66	6.12	(1.87, 10.36)	< 0.001
miR-339-3p	2	66	6.12	(1.87, 10.36)	< 0.001
miR-361	2	87	7.44	(4.64, 10.25)	< 0.001
miR-376a	2	118	7.81	(5.00, 10.61)	< 0.001
miR-423-5p	7	408	7.60	(6.10, 9.10)	< 0.001
miR-494-3p	2	46	6.26	(3.43, 9.10)	< 0.001
miR-499-5p	2	206	8.48	(4.71, 12.25)	< 0.001
miR-518e	2	138	8.31	(5.52, 11.11)	< 0.001

miR-622	2	116	7.76	(4.95, 10.57)	< 0.001
miR-654	2	50	6.49	(3.66, 9.32)	< 0.001
miR-660-3p	2	30	5.21	(2.32, 8.10)	< 0.001
miR-662	2	138	8.31	(5.52, 11.11)	< 0.001
miR-675	2	116	7.76	(4.95, 10.57)	< 0.001
miR-744	4	151	6.73	(4.72, 8.74)	< 0.001
miR-1285-3p	2	84	7.33	(4.52, 10.14)	< 0.001

Supplementary Table 6.Statistically significant down-regulated miRNAs in overall analysis (n=10)

miRNA	No. of studies	No. of samples	logOR	95%CI	*P* values
miR-30c	3	173	6.62	(3.04, 10.20)	< 0.001
miR-92a-3p	2	23	4.87	(1.97, 7.78)	< 0.001
miR-150-5p	2	112	7.97	(5.17, 10.77)	< 0.001
miR-181a	2	39	5.52	(2.47, 8.57)	< 0.001
miR-181d-5p	2	66	6.12	(1.87, 10.36)	< 0.001
miR-186	2	87	6.37	(1.62, 11.13)	0.001
miR-200a	2	138	8.31	(5.52, 11.11)	< 0.001
miR-328-3p	2	210	9.13	(6.34, 11.92)	< 0.001
miR-574-5p	2	98	6.52	(1.47, 11.56)	< 0.001
miR-623	2	98	6.52	(1.47, 11.56)	< 0.001

Supplementary Table 7. Inconsistently dysregulated miRNAs classified by different subgroups.

miRNA	blood			tissue		
	**No. of studies**	**No. of samples**	**expression**	**No. of studies**	**No. of samples**	**expression**
let-7e	3	126	up	1	10	down
let-7f	1	27	down	1	10	up
miR-142	3	47	down	1	12	up
miR-152	1	60	up	1	12	down
**miRNA**	**human**			**animal**		
	**No. of studies**	**No. of samples**	**expression**	**No. of studies**	**No. of samples**	**expression**
miR-125a-5p	2	120	down	1	19	up
miR-128	1	61	up	1	17	down
miR-130a-3p	1	24	down	1	27	up
miR-133a	2	305	up	1	12	down
miR-144	1	12	up	1	92	down
miR-27b-3p	1	17	down	1	27	up
**miRNA**	**Asian**			**non-Asian**		
	**No. of studies**	**No. of samples**	**expression**	**No. of studies**	**No. of samples**	**expression**

miR-126	1	81	up	1	65	down
miR-129-5p	2	317	down	1	24	up
miR-132	1	16	down	1	6	up
miR-140-3p	1	24	up	1	17	down
miR-155	1	170	up	2	90	down
miR-192	1	86	down	1	6	up
miR-193b-5p	1	27	up	1	180	down
miR-206	1	24	up	1	6	down
miR-330-3p	1	24	down	2	66	up
miR-340	1	10	up	2	66	up
miR-425	1	62	up	1	6	down
miR-494	1	22	up	2	186	down
miR-93	1	16	down	1	20	up

Supplementary Table 8.Statistically significant dysregulated miRNAs in blood (n= 45, 35 upregulated and 10 downregulated)

	**miRNA**	**No. of studies**	**No. of samples**	**logOR**	**95%CI**	***P* values**
	miR-15b	3	52	5.57	(3.23, 7.92)	< 0.001
up-regulated	miR-18a-5p	2	67	6.07	(1.92, 10.23)	< 0.001
	miR-18b-5p	2	85	7.29	(4.48, 10.11)	< 0.001
	miR-20a-5p	3	76	6.01	(3.67, 8.35)	< 0.001
	miR-21-5p	4	230	7.60	(5.61, 9.59)	< 0.001
	miR-23b-3p	2	23	4.87	(1.97, 7.78)	< 0.001
	miR-24	2	30	5.22	(2.33, 8.12)	< 0.001
	miR-26b-5p	2	88	7.40	(4.59, 10.21)	< 0.001
	miR-29a-3p	3	52	5.52	(3.17, 7.87)	< 0.001
	miR-92b	3	197	8.35	(6.07, 10.63)	< 0.001
	miR-103-3p	2	49	6.45	(3.62, 9.27)	< 0.001
	miR-106b-5p	2	47	6.37	(3.54, 9.20)	< 0.001
	miR-107-3p	2	34	6.22	(3.38, 9.05)	< 0.001
	miR-122	2	149	8.46	(5.67, 11.26)	< 0.001
	miR-130b	2	59	6.76	(3.94, 9.58)	< 0.001
	miR-133-3p	2	77	6.98	(4.15, 9.80)	< 0.001
	miR-151-5p	2	33	5.34	(2.45, 8.23)	< 0.001
	miR-155-5p	3	168	7.39	(5.08, 9.71)	< 0.001
	miR-200b	2	173	8.89	(6.11, 11.68)	< 0.001

	miR-210-3p	5	209	7.16	(5.38, 8.94)	< 0.001
	miR-214	2	86	6.36	(1.63, 11.10)	< 0.001
	miR-216a	2	188	9.00	(6.22, 11.79)	< 0.001
	miR-330	2	66	6.12	(1.87, 10.36)	< 0.001
	miR-361	2	87	7.44	(4.64, 10.25)	< 0.001
	miR-376a	2	118	7.81	(5.00, 10.61)	< 0.001
	miR-494-3p	2	46	6.26	(3.43, 9.10)	< 0.001
	miR-499-5p	2	206	8.48	(4.71, 12.25)	< 0.001
	miR-518e	2	138	8.31	(5.52, 11.11)	< 0.001
	miR-622	2	116	7.76	(4.95, 10.57)	< 0.001
	miR-654	2	50	6.49	(3.66, 9.32)	< 0.001
	miR-660-3p	2	30	5.21	(2.32, 8.10)	< 0.001
	miR-622	2	138	8.31	(5.52, 11.11)	< 0.001
	miR-675	2	116	7.76	(4.95, 10.57)	< 0.001
	miR-744	4	151	6.73	(4.72, 8.74)	< 0.001
	miR-1285-3p	2	84	7.33	(4.52, 10.14)	< 0.001
	miR-30c	3	173	6.62	(3.04, 10.20)	< 0.001
down-regulated	miR-92a-3p	2	23	4.87	(1.97, 7.78)	< 0.001
	miR-142-3p	2	41	6.11	(3.27, 8.95)	< 0.001
	miR-150-5p	2	112	7.97	(5.17, 10.77)	< 0.001
	miR-181a	2	39	5.52	(2.47, 8.57)	< 0.001

miR-181d-5p	2	66	6.12	(1.87, 10.36)	< 0.001
miR-186	2	87	6.37	(1.62, 11.13)	< 0.001
miR-200a	2	138	8.31	(5.52, 11.11)	< 0.001
miR-574-5p	2	98	6.52	(1.47, 11.56)	< 0.001
miR-623	2	98	6.52	(1.47, 11.56)	< 0.001

Supplementary Table 9.Statistically significant dysregulated miRNAs in plasma (n= 45, 21 upregulated and 2 downregulated）and serum (n= 3, 2 upregulated and 1 downregulated）

source	direction	miRNA	No. of studies	No. of samples	logOR	95%CI	*P* values
		miR-18b-5p	2	85	7.29	(4.48, 10.11)	< 0.001
		miR-26b-5p	2	88	7.40	(4.59, 10.21)	< 0.001
		miR-103-3p	2	49	6.45	(3.62, 9.27)	< 0.001
		miR-106b-5p	2	47	6.37	(3.54, 9.20)	< 0.001
plasma	up-regulated	miR-107-3p	2	34	6.22	(3.38, 9.05)	< 0.001
		miR-130b	2	59	6.76	(3.94, 9.58)	< 0.001
		miR-155-5p	3	168	7.39	(5.08, 9.71)	< 0.001
		miR-210-3p	5	209	7.16	(5.38, 8.94)	< 0.001
		miR-216a	2	188	9.00	(6.22, 11.79)	< 0.001
		miR-361	2	87	7.44	(4.64, 10.25)	< 0.001
		miR-494-3p	2	46	6.26	(3.43, 9.10)	< 0.001
		miR-499-5p	2	206	8.48	(4.71, 12.25)	< 0.001
		miR-654	2	50	6.49	(3.66, 9.32)	< 0.001
		miR-1285-3p	2	84	7.33	(4.52, 10.14)	< 0.001
		miR-1	2	43	6.16	(3.33, 9.00)	< 0.001
		miR-20a	2	70	6.96	(4.15, 9.78)	< 0.001
		miR-20b-5p	2	47	6.37	(3.54, 9.20)	< 0.001
		miR-21	5	376	7.85	(6.05, 9.65)	< 0.001

		miR-29a-3p	2	46	6.26	(3.43, 9.10)	< 0.001
		miR-221	2	63	6.91	(4.09, 9.72)	< 0.001
		miR-223-3p	2	81	7.13	(4.31, 9.94)	< 0.001
plasma	down-regulated	miR-142-3p	2	41	6.11	(3.27, 8.95)	< 0.001
		miR-15b	2	46	6.34	(3.51, 9.17)	< 0.001
		miR-21	3	237	8.50	(6.22, 10.78)	< 0.001
serum	up-regulated	miR-92b	3	197	8.35	(6.07, 10.63)	< 0.001
	down-regulated	miR-129-5p	2	317	10.11	(7.33, 12.89)	< 0.001

Supplementary Table 10.Statistically significant dysregulated miRNAs in Asian countries (n=11, 9 upregulated and 2 downregulated)

	miRNA	No. of studies	No. of samples	logOR	95%CI	*P* values
	let-7i-5p	3	139	7.12	(4.82, 9.43)	< 0.001
up-regulated	miR-103	2	49	6.45	(3.62, 9.27)	< 0.001
	miR-122-5p	2	74	7.07	(4.26, 9.89)	< 0.001
	miR-195	3	73	6.38	(4.07, 8.69)	< 0.001
	miR-199a-5p	2	74	7.07	(4.26, 9.89)	< 0.001
	miR-210	2	103	7.51	(4.70, 10.32)	< 0.001
	miR-216a	2	188	9.00	(6.22, 11.79)	< 0.001
	miR-518e	2	138	8.31	(5.52, 11.11)	< 0.001
	miR-622	2	116	7.76	(4.95, 10.57)	< 0.001
down-regulated	miR-129	3	374	9.37	(7.10, 11.65)	< 0.001
	miR-200a	2	138	8.31	(5.52, 11.11)	< 0.001

Supplementary Table 11.Statistically significant dysregulated miRNAs in non-Asian countries (n=22, 16 upregulated and 6 downregulated)

	**miRNA**	**No. of studies**	**No. of samples**	**logOR**	**95%CI**	***P* values**
	let-7a-5p	2	23	4.87	(1.97, 7.78)	< 0.001
	let-7e-5p	3	126	6.90	(4.59, 9.21)	< 0.001
up-regulated	miR-1	2	43	6.16	(3.33, 9.00)	< 0.001
	miR-18a	2	67	6.07	(1.92, 10.23)	< 0.001
	miR-18b	2	85	7.29	(4.48, 10.11)	< 0.001
	miR-24	2	30	5.22	(2.33, 8.12)	< 0.001
	miR-28	2	18	4.53	(1.61, 7.45)	< 0.001
	miR-28-3p	2	18	4.53	(1.61, 7.45)	< 0.001
	miR-133	2	77	6.98	(4.15, 9.80)	< 0.001
	miR-339-3p	2	66	6.12	(1.87, 10.36)	< 0.001
	miR-376	2	118	7.81	(5.00, 10.61)	< 0.001
	miR-376a	2	118	7.81	(5.00, 10.61)	< 0.001
	miR-499-5p	2	206	8.48	(4.71, 12.25)	< 0.001
	miR-654	2	50	6.49	(3.66, 9.32)	< 0.001
	miR-622	2	128	8.31	(5.52, 11.11)	< 0.001
	miR-675	2	116	7.76	(4.95, 10.57)	< 0.001
	miR-30c	3	173	6.62	(3.04, 10.20)	0.001

	miR-150	2	100	7.74	(4.94, 10.54)	< 0.001
down-regulated	miR-181d	2	66	6.12	(1.87, 10.36)	< 0.001
	miR-328	2	210	9.13	(6.34, 11.92)	< 0.001
	miR-574-5p	2	98	6.52	(1.47, 11.56)	< 0.001
	miR-623	2	98	6.52	(1.47, 11.56)	< 0.001

Supplementary Table 12.Statistically significant dysregulated miRNAs in human (n= 39, 30 upregulated and 9 downregulated)

	miRNA	No. of studies	No. of samples	logOR	95%CI	*P* values
	let-7a-5p	2	23	4.87	(1.97, 7.78)	< 0.001
	let-7i-5p	2	129	8.21	(5.41, 11.01)	< 0.001
	miR-122	2	149	8.46	(5.67, 11.26)	< 0.001
	miR-1285-3p	2	84	7.33	(4.52, 10.14)	< 0.001
up-regulated	miR-130b	2	59	6.75	(3.94, 9.58)	< 0.001
	miR-133a	2	305	9.89	(7.10, 12.67)	< 0.001
	miR-155-5p	2	141	7.44	(3.94, 11.54)	< 0.001
	miR-15b	3	52	5.57	(3.23, 7.92)	< 0.001
	miR-18b-5p	2	85	7.29	(4.48, 10.11)	< 0.001
	miR-195-3p	3	73	6.38	(4.07, 8.69)	< 0.001
	miR-200a	2	138	8.31	(5.52, 11.11)	< 0.001
	miR-200b	2	173	8.89	(6.11, 11.68)	< 0.001
	miR-20a-5p	3	76	6.01	(3.67, 8.35)	< 0.001
	miR-21	10	709	8.02	(6.76, 9.27)	< 0.001
	miR-210	3	163	7.75	(5.46, 10.04)	< 0.001
	miR-214	2	86	6.36	(1.63, 11.10)	< 0.001
	miR-216a	2	188	9.00	(6.22, 11.79)	< 0.001
	miR-23b-3p	2	23	4.87	(1.97, 7.78)	< 0.001
	miR-339-3p	2	66	6.12	(1.87, 10.36)	< 0.001

	miR-376a	2	118	7.81	(5.00, 10.61)	< 0.001
	miR-423-5p	6	388	7.85	(6.23, 9.47)	< 0.001
	miR-499-5p	2	206	8.48	(4.71, 12.25)	< 0.001
	miR-518e	2	138	8.31	(5.52, 11.11)	< 0.001
	miR-574-5p	2	98	6.52	(1.47, 11.56)	< 0.001
	miR-622	2	116	7.76	(4.95, 10.57)	< 0.001
	miR-660-3p	2	30	5.21	(2.32, 8.10)	< 0.001
	miR-622	2	138	8.31	(5.52, 11.11)	< 0.001
	miR-675	2	116	7.76	(4.95, 10.57)	< 0.001
	miR-744	4	151	6.73	(4.72, 8.74)	< 0.001
	miR-92b	3	197	8.35	(6.07, 10.63)	< 0.001
	miR-125a-5p	2	120	8.16	(5.37, 10.96)	< 0.001
	miR-150-5p	2	112	7.97	(5.17, 10.77)	< 0.001
down-regulated	miR-181a	2	39	5.52	(2.47, 8.57)	< 0.001
	miR-181d-5p	2	66	6.12	(1.87, 10.36)	< 0.001
	miR-186	2	87	6.37	(1.62, 11.13)	< 0.001
	miR-30c	2	156	7.01	(0.99, 13.03)	< 0.001
	miR-328-3p	2	210	9.13	(6.34, 11.92)	< 0.001
	miR-623	2	98	6.52	(1.47, 11.56)	< 0.001
	miR-92a-3p	2	23	4.87	(1.97, 7.78)	< 0.001

Supplementary Table 13.Statistically significant dysregulated miRNAs in animals' model (n=10, 9 upregulated and 1 downregulated)

	miRNA	No. of studies	No. of samples	logOR	95%CI	*P* values
up-regulated	miR-106b-5p	2	47	6.37	(3.54, 9.20)	< 0.001
	miR-20b-5p	2	47	6.37	(3.54, 9.20)	< 0.001
	miR-21-5p	2	46	6.26	(3.43, 9.10)	< 0.001
	miR-29a-3p	2	46	6.26	(3.43, 9.10)	< 0.001
	miR-122-5p	2	74	7.07	(4.26, 9.89)	< 0.001
	miR-133b-3p	2	43	6.15	(3.31, 8.99)	< 0.001
	miR-199a-5p	2	74	7.07	(4.26, 9.89)	< 0.001
	miR-210-3p	2	46	6.26	(3.43, 9.10)	< 0.001
	miR-494	2	46	6.26	(3.43, 9.10)	< 0.001
down-regulated	miR-132-3p	2	40	6.06	(3.22, 8.89)	< 0.001

Supplementary Table 14.Statistically significant dysregulated miRNAs in sensitivity analysis according to sample sizes (n= 46, 42 upregulated and 4 downregulated)

	miRNA	No. of studies	No. of samples	logOR	95%CI	*P* values
up-regulated	let-7a-5p	2	27	5.29	(2.42, 8.17)	< 0.001
	let-7c-5p	2	27	5.29	(2.42, 8.17)	< 0.001
	let-7i-5p	3	139	7.12	(4.82, 9.43)	< 0.001
	miR-15b	2	46	6.34	(3.51, 9.17)	< 0.001
	miR-18a-5p	2	67	6.07	(1.92, 10.23)	< 0.001
	miR-18b-5p	2	85	7.27	(4.48, 10.11)	< 0.001
	miR-20a-5p	2	70	6.96	(4.15, 9.78)	< 0.001
	miR-21	10	709	8.02	(6.76, 9.27)	< 0.001
	miR-21-5p	4	230	7.60	(5.61, 9.59)	< 0.001
	miR-26b-5p	2	88	7.40	(4.59, 10.21)	< 0.001
	miR-29a-3p	2	44	6.26	(3.43, 9.10)	< 0.001
	miR-92b	3	197	8.35	(6.07, 10.63)	< 0.001
	miR-103-3p	2	49	6.45	(3.62, 9.27)	< 0.001
	miR-106b-5p	2	47	6.37	(3.54, 9.20)	< 0.001
	miR-107-3p	2	44	6.22	(3.38, 9.05)	< 0.001
	miR-122-5p	4	213	7.77	(5.79, 9.76)	< 0.001
	miR-130b	2	59	6.76	(3.94, 9.58)	< 0.001

miR-133-3p	2	77	6.98	(4.15, 9.80)	< 0.001
miR-133b-3p	2	43	6.15	(3.31, 8.99)	< 0.001
miR-148a-3p	2	84	7.34	(4.53, 10.15)	< 0.001
miR-155-5p	3	167	7.39	(5.08, 9.71)	< 0.001
miR-181b-5p	2	41	6.11	(3.27, 8.95)	< 0.001
miR-186b-5p	2	85	7.29	(4.48, 10.11)	< 0.001
miR-195-3p	3	73	6.38	(4.07, 8.69)	< 0.001
miR-199a	3	73	6.38	(4.07, 8.69)	< 0.001
miR-200b	2	173	8.89	(6.11, 11.68)	< 0.001
miR-210-3p	5	209	8.22	(4.27, 12.17)	< 0.001
miR-216a	2	188	9.00	(6.22, 11.79)	< 0.001
miR-223-3p	2	81	7.13	(4.31, 9.94)	< 0.001
miR-330	2	66	6.12	(1.87, 10.36)	< 0.001
miR-361	2	87	7.44	(4.64, 10.25)	< 0.001
miR-376a	2	118	7.81	(5.00, 10.61)	< 0.001
miR-423-5p	7	408	7.60	(6.10, 9.10)	< 0.001
miR-494-3p	2	46	6.26	(3.43, 9.10)	< 0.001
miR-499-5p	2	206	8.48	(4.71, 12.25)	< 0.001
miR-518e	2	138	8.31	(5.52, 11.11)	< 0.001
miR-622	2	116	7.76	(4.95, 10.57)	< 0.001
miR-654	2	50	6.49	(3.66, 9.32)	< 0.001

	miR-662	2	138	8.31	(5.52, 11.11)	< 0.001
	miR-675	2	116	7.76	(4.95, 10.57)	< 0.001
	miR-744	3	145	7.58	(5.28, 9.87)	< 0.001
	miR-1285-3p	2	84	7.33	(4.52, 10.14)	< 0.001
down-regulated	miR-30c	2	167	7.93	(3.76, 12.10)	< 0.001
	miR-150-5p	2	112	7.97	(5.17, 10.77)	< 0.001
	miR-200a	2	138	8.31	(5.52, 11.11)	< 0.001
	miR-328-3p	2	210	9.13	(6.34, 11.92)	< 0.001

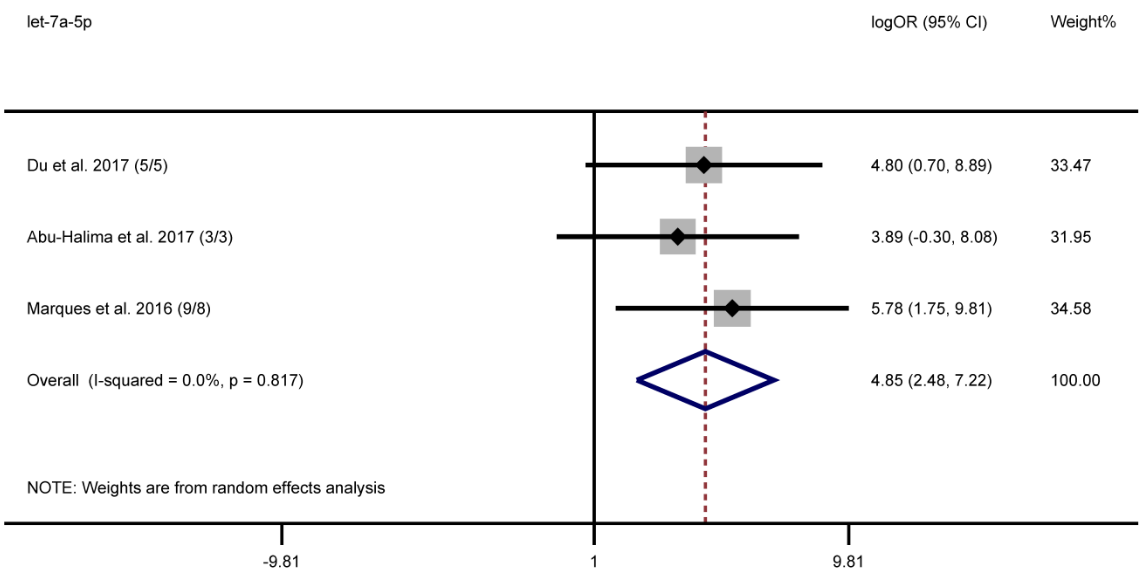

Supplementary Figure1. Forest plot of let-7a-5p

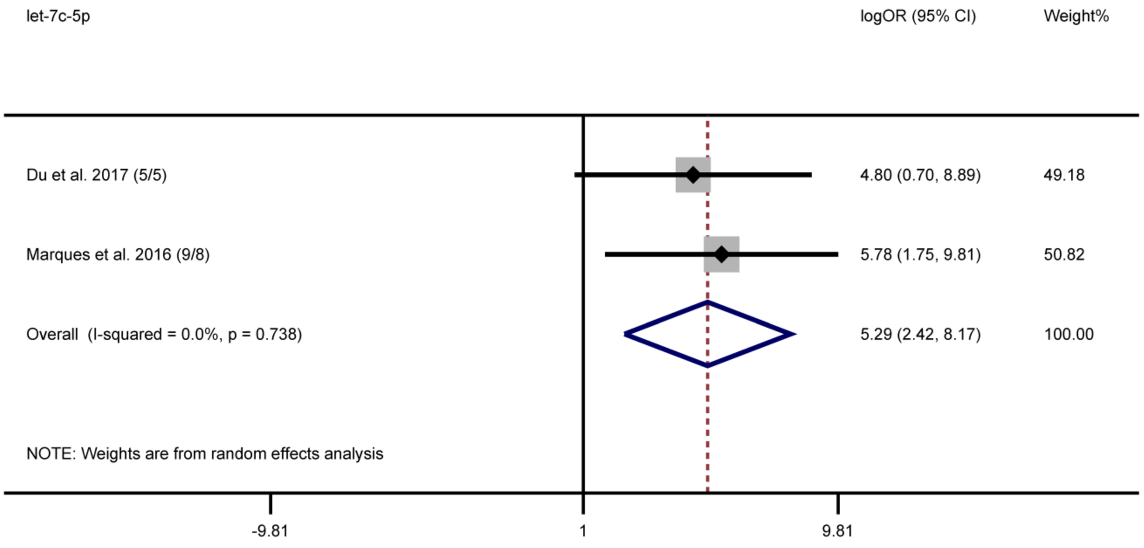

Supplementary Figure2. Forest plot of let-7c-5p

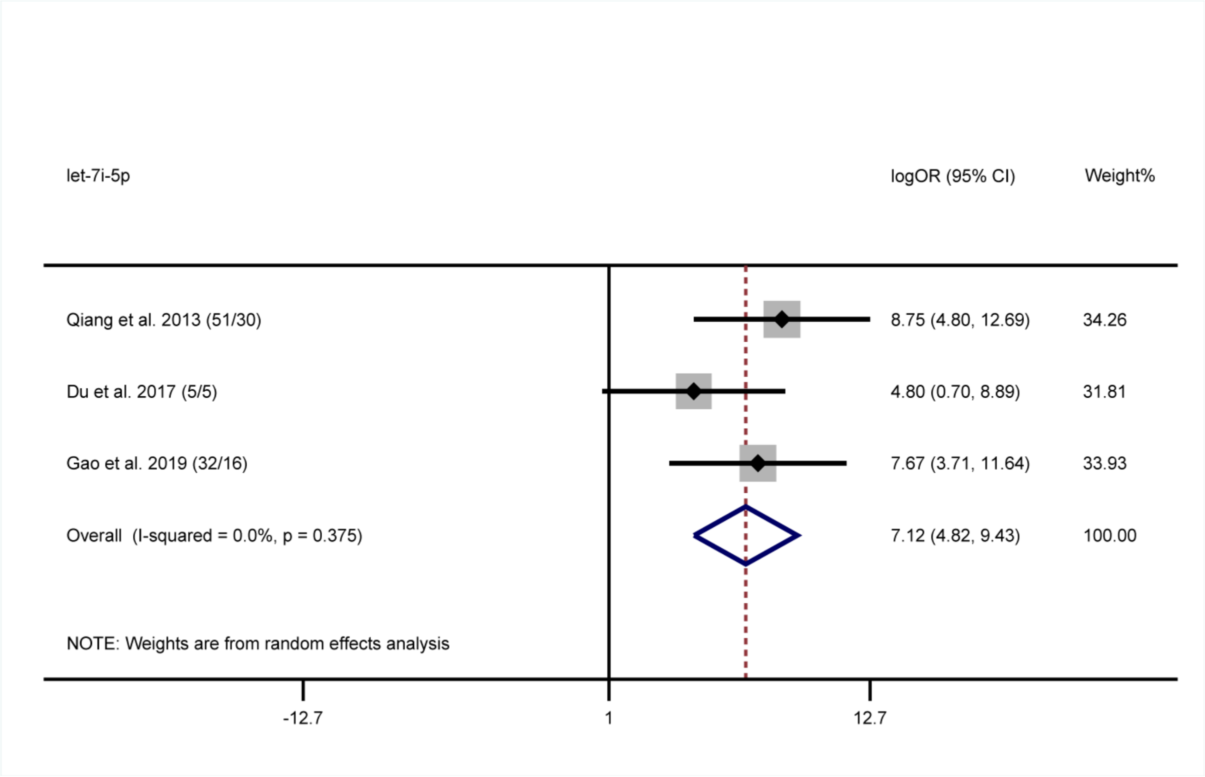

Supplementary Figure3. Forest plot of let-7i-5p

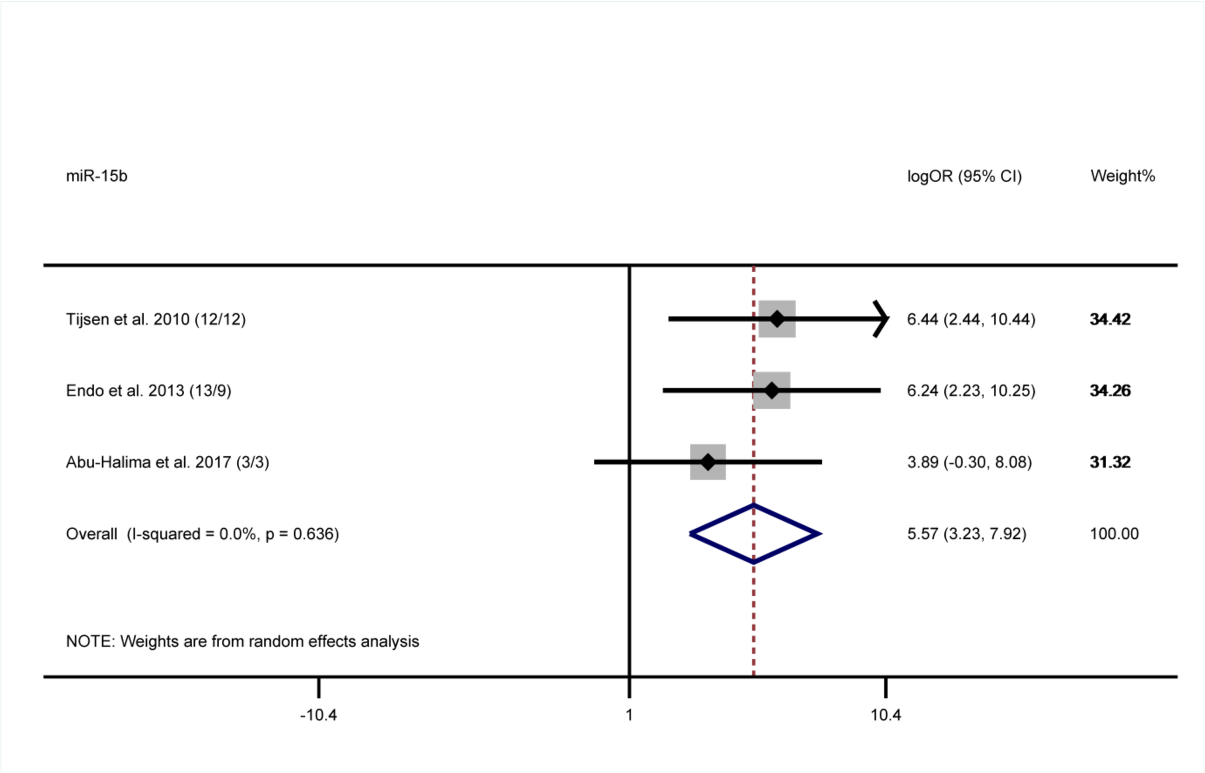

Supplementary Figure4. Forest plot of miR-15b

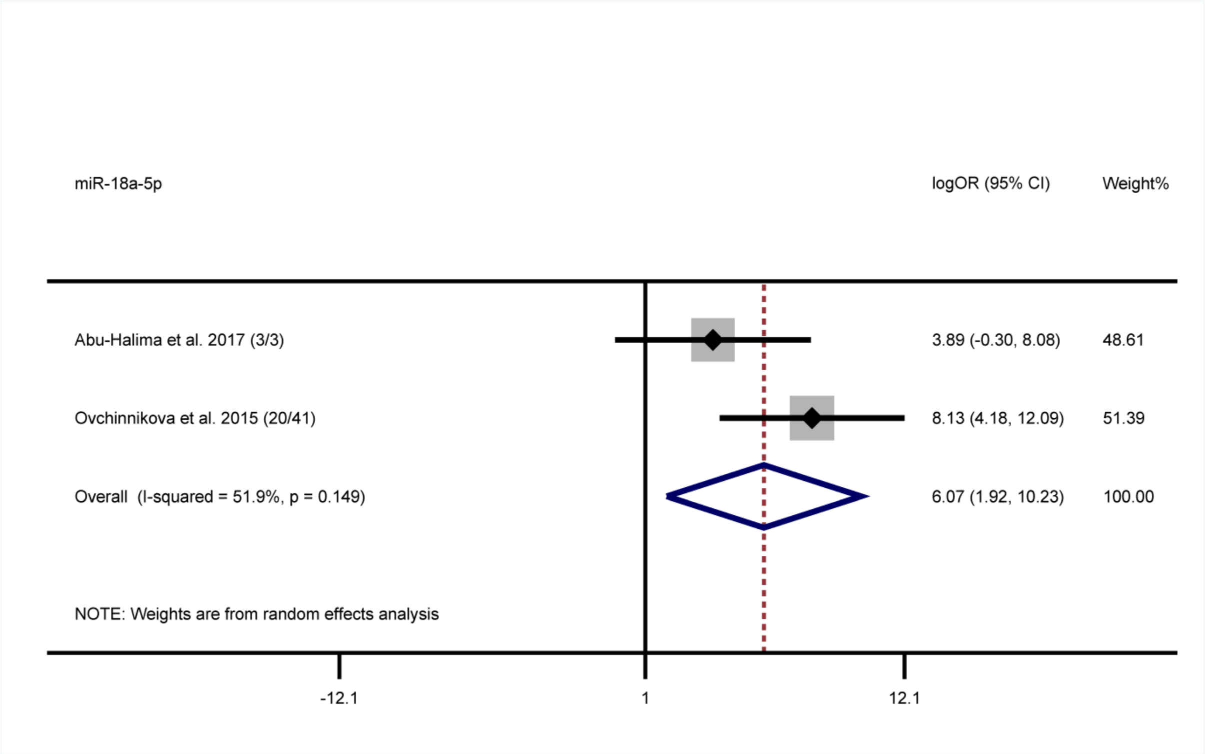

Supplementary Figure5. Forest plot of miR-18a-5p

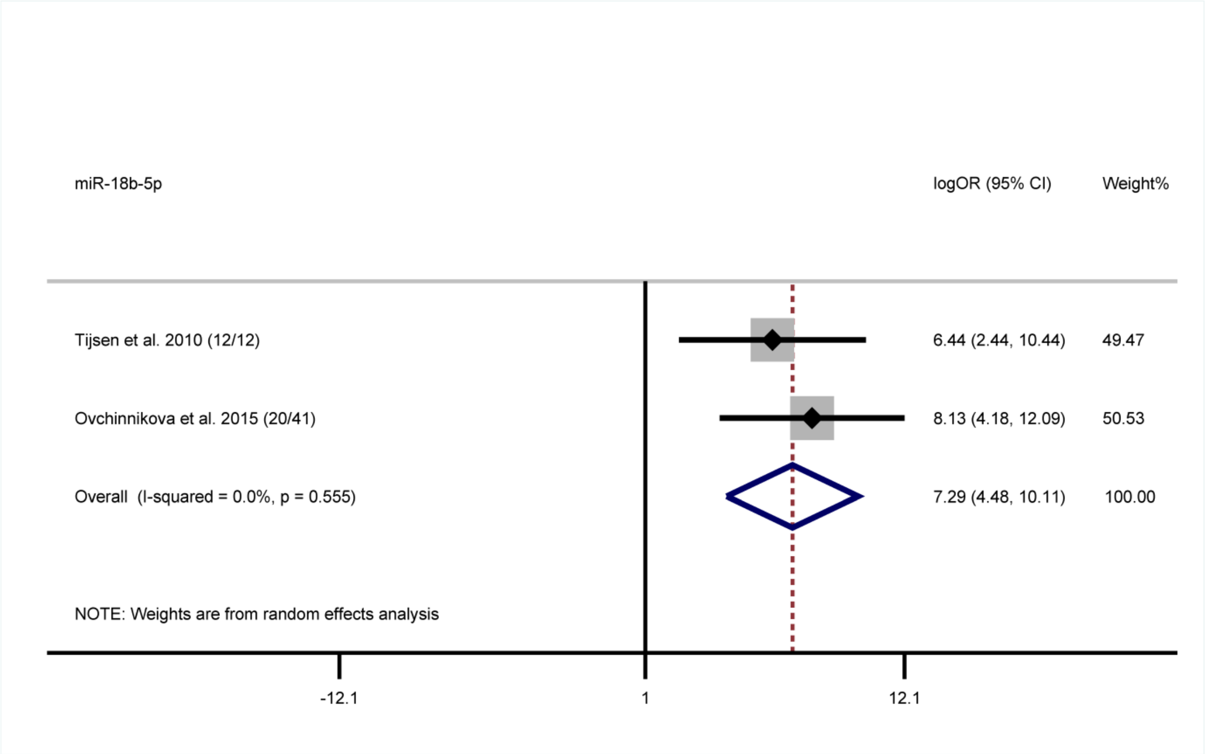

Supplementary Figure6. Forest plot of miR-18b-5p

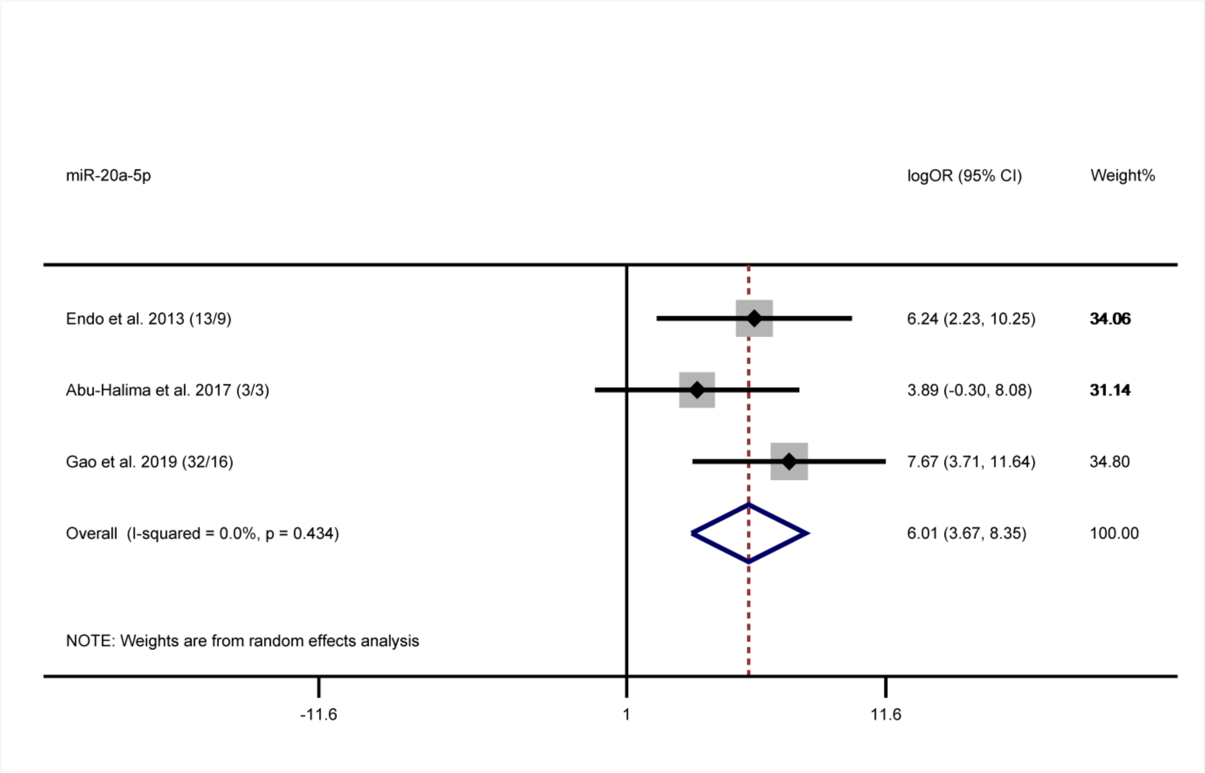

Supplementary Figure 7. Forest plot of miR-20a-5p

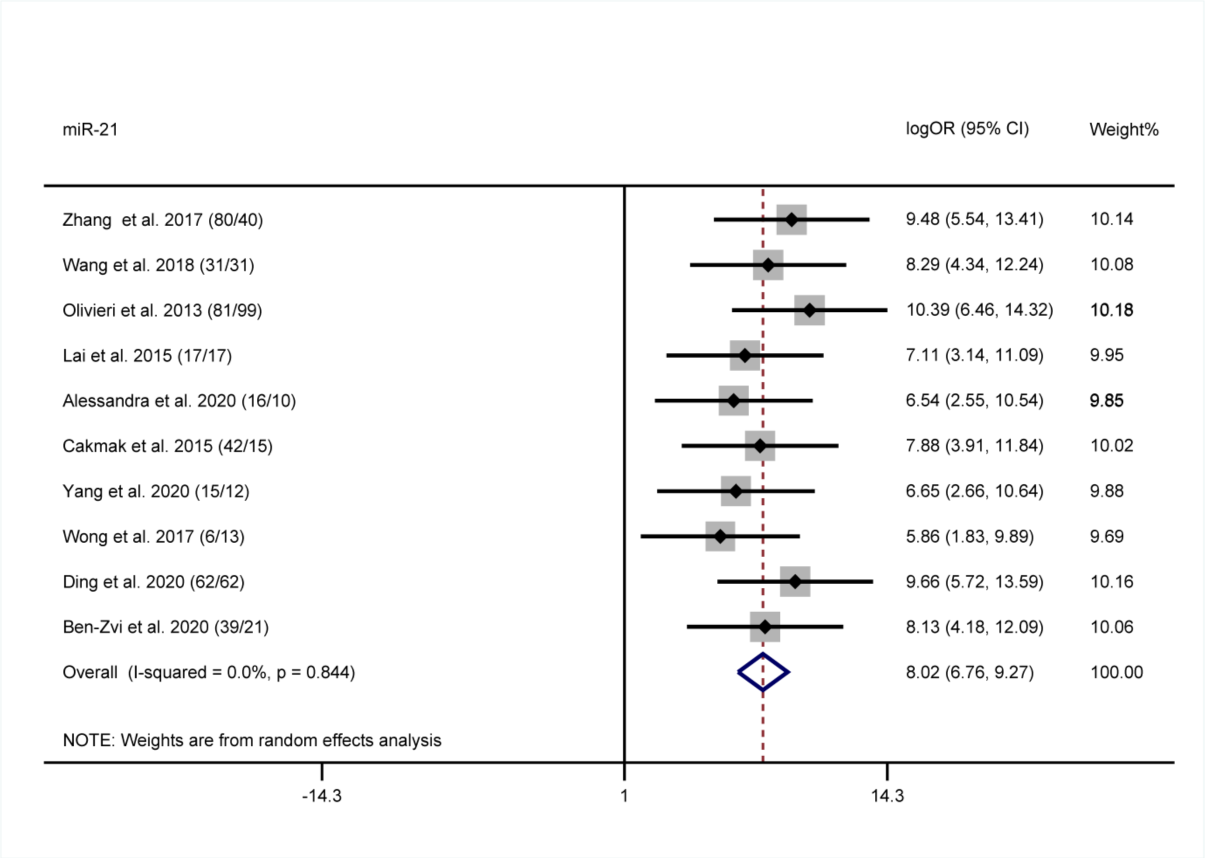

Supplementary Figure8. Forest plot of miR-21

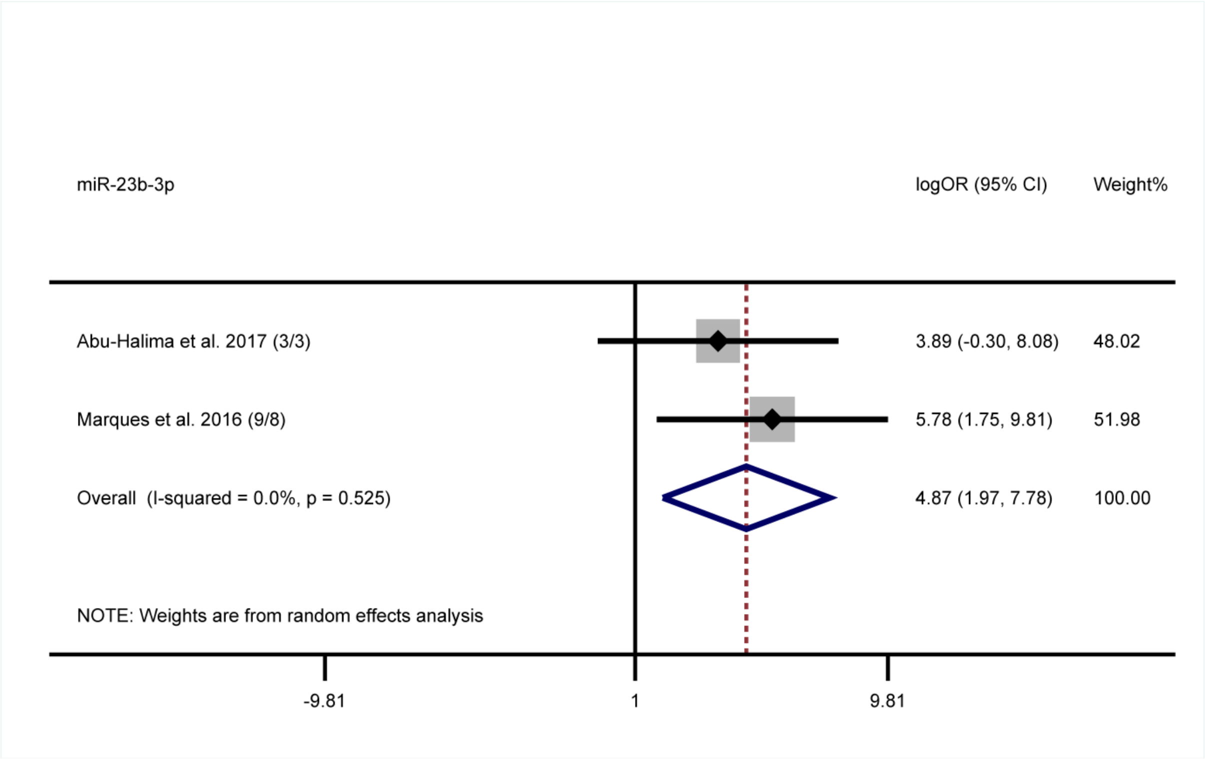

Supplementary Figure9. Forest plot of miR-23b-3p

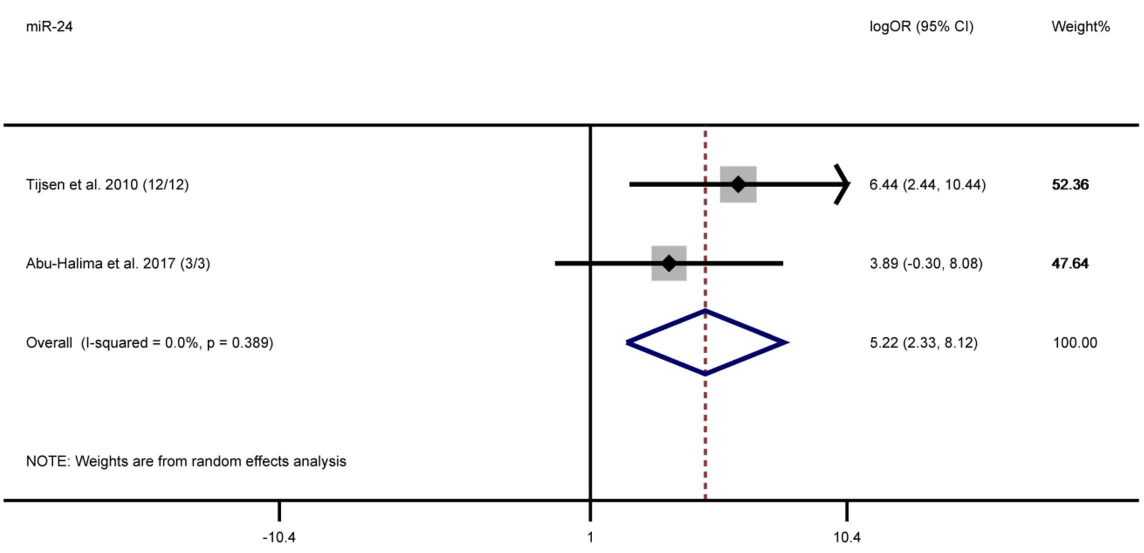

Supplementary Figure 10. Forest plot of miR-24

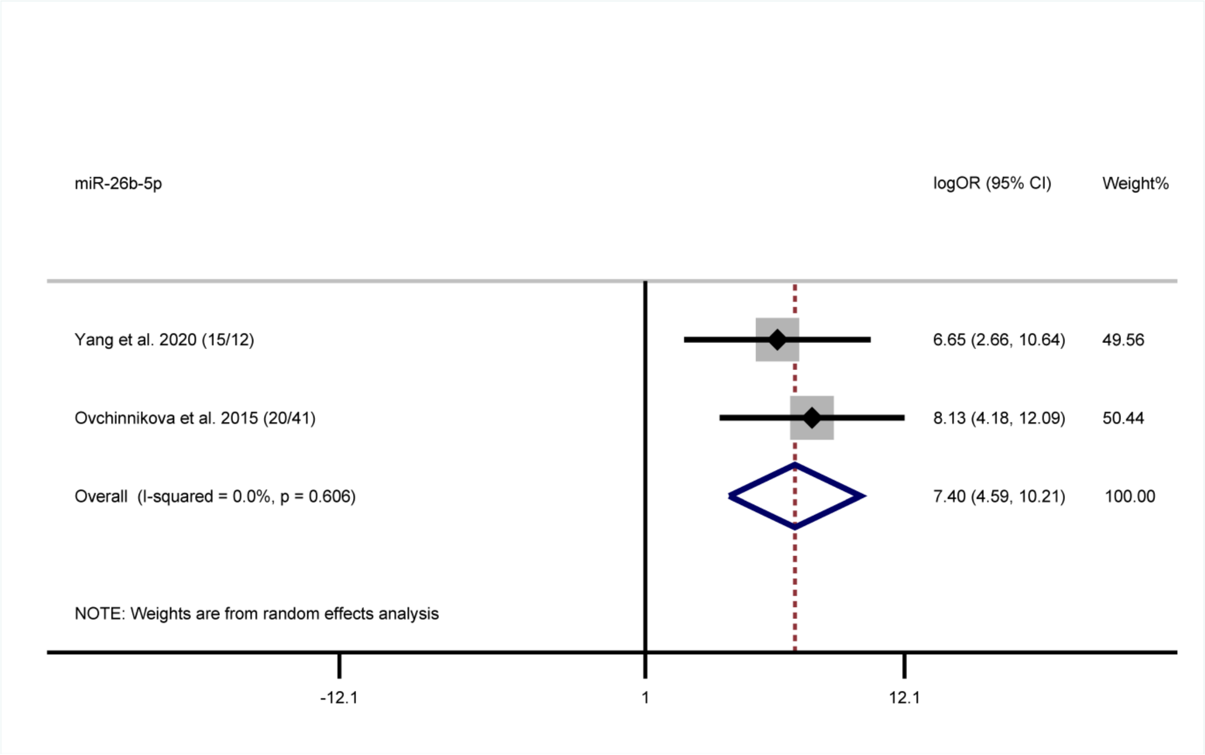

Supplementary Figure 11. Forest plot of miR-26b-5p

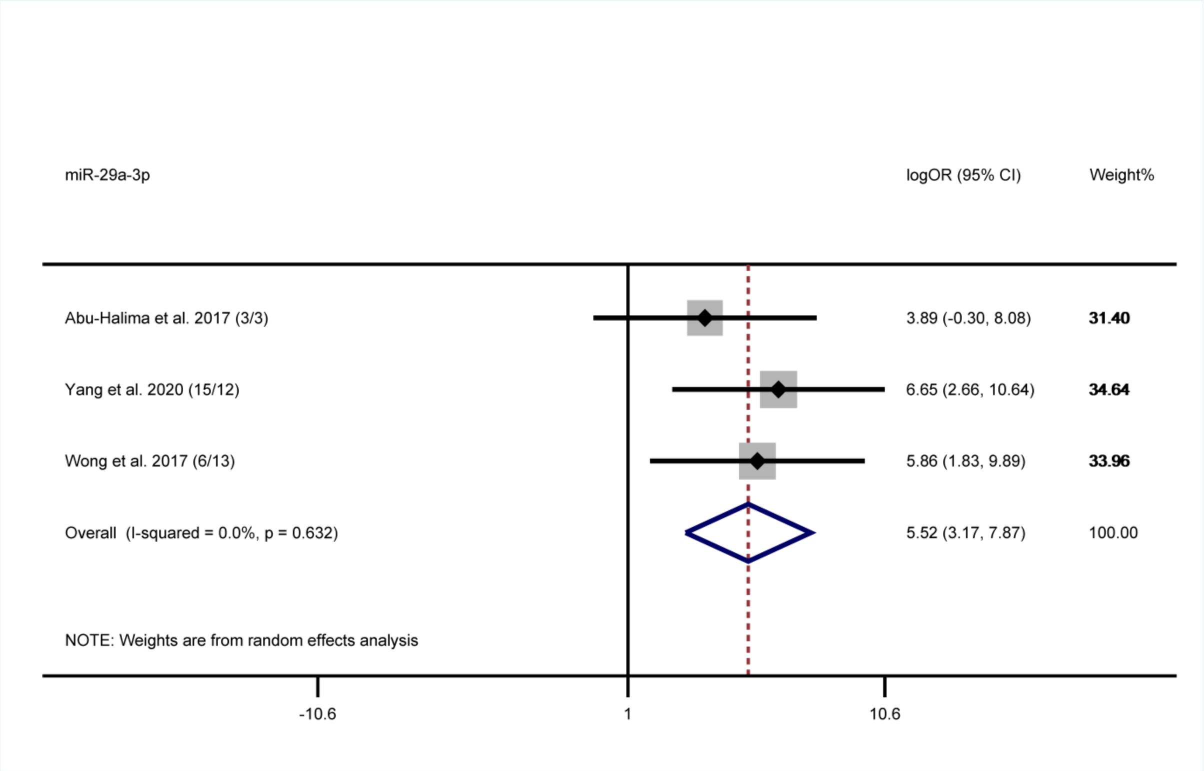

Supplementary Figure12. Forest plot of miR-29a-3p

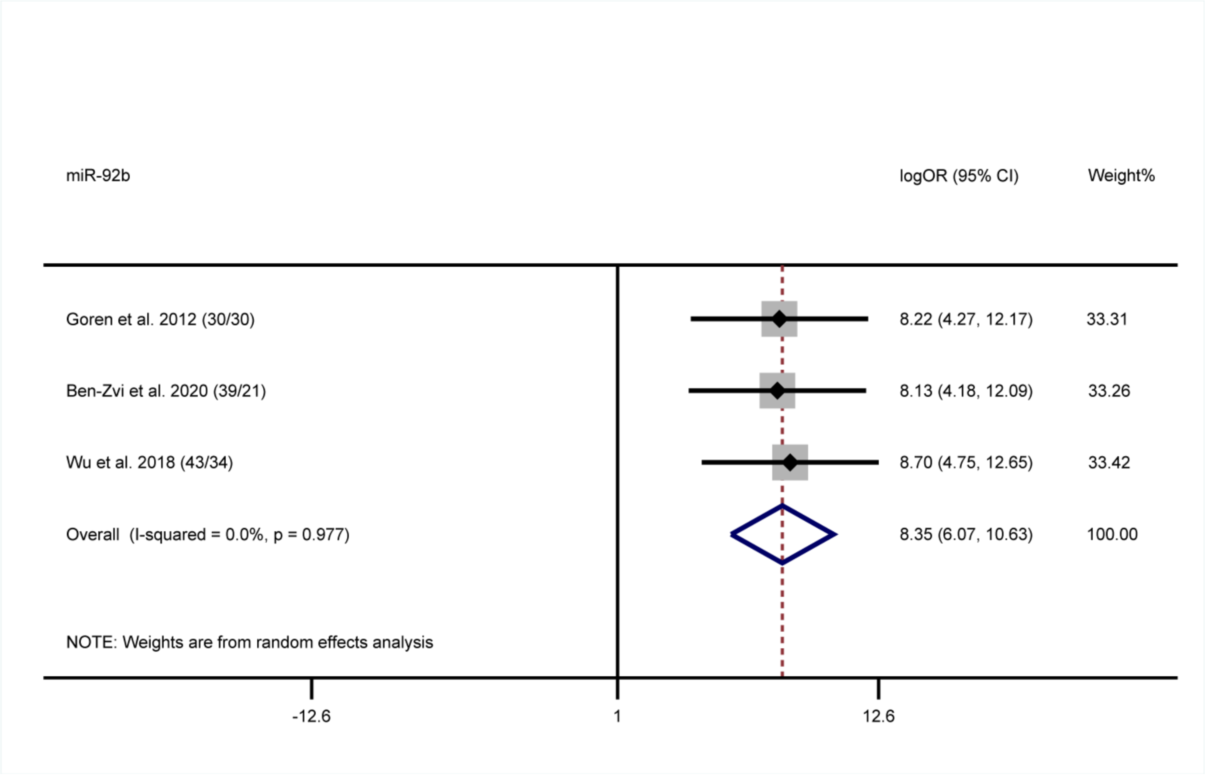

Supplementary Figure13. Forest plot of miR-92b

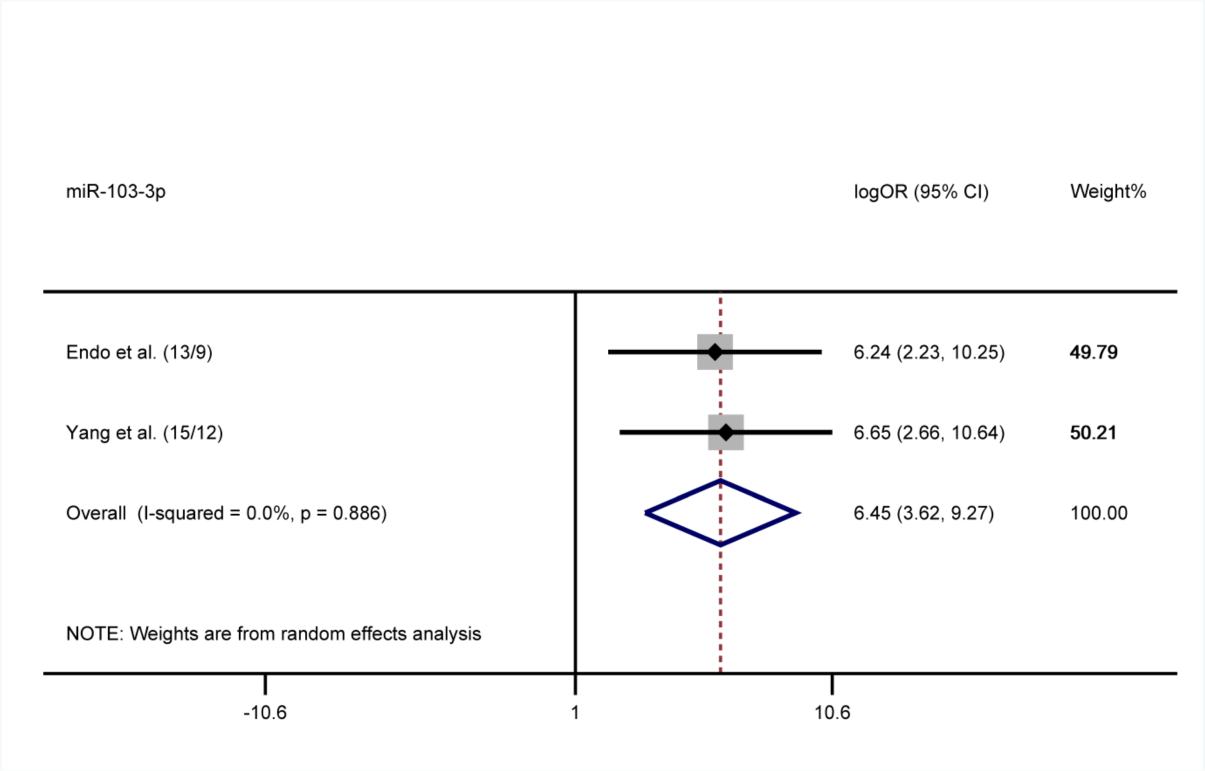

Supplementary Figure14. Forest plot of miR-103-3p

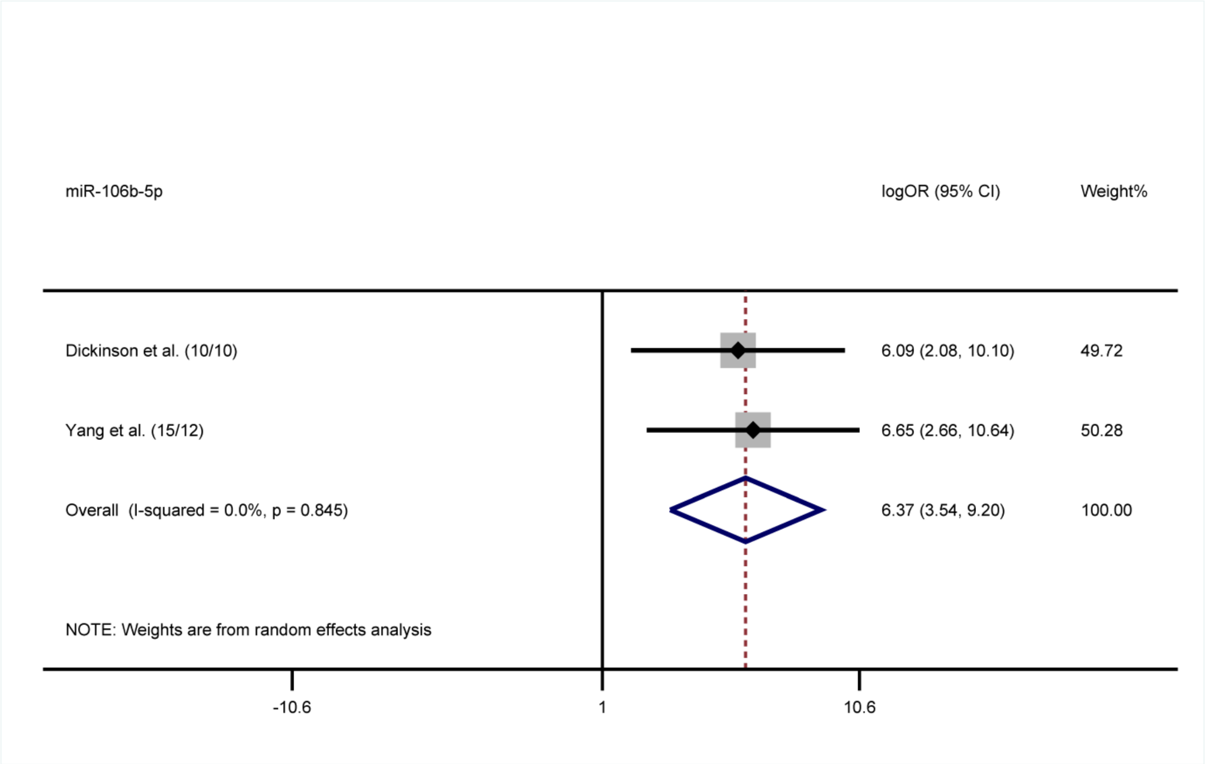

Supplementary Figure 15. Forest plot of miR-106b-5p

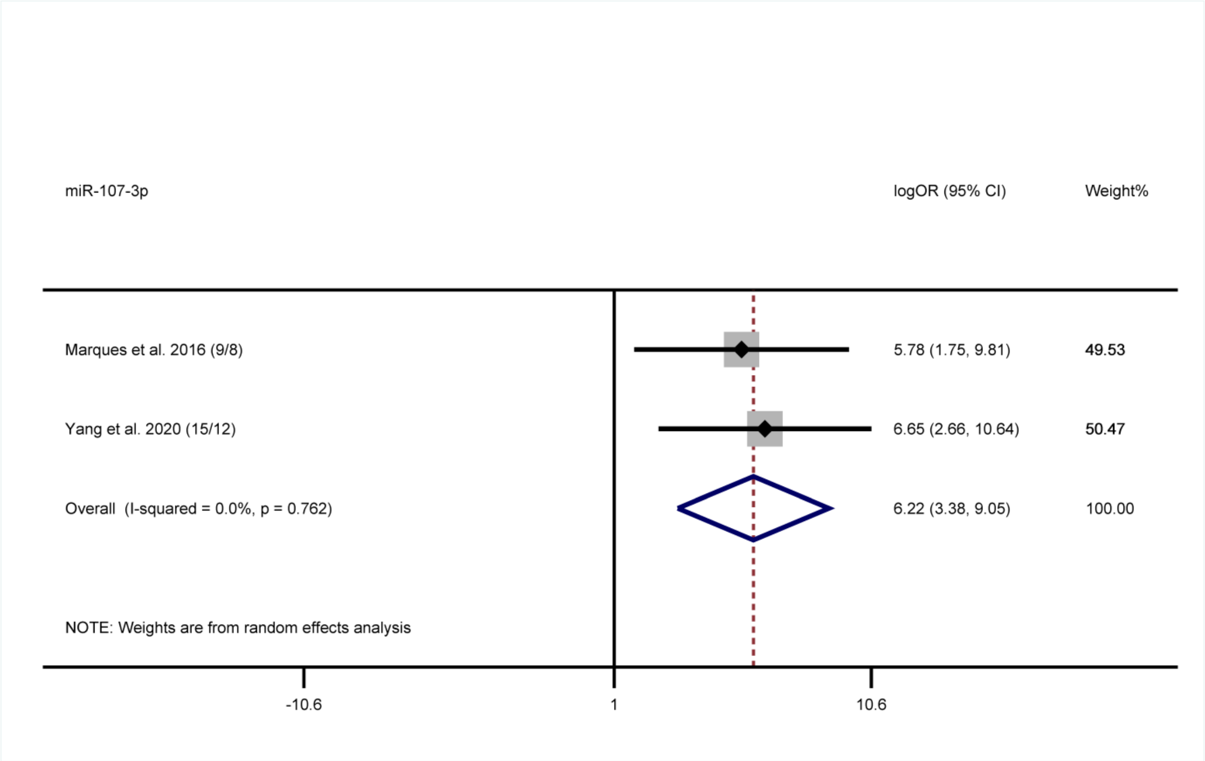

Supplementary Figure 16. Forest plot of miR-107-3p

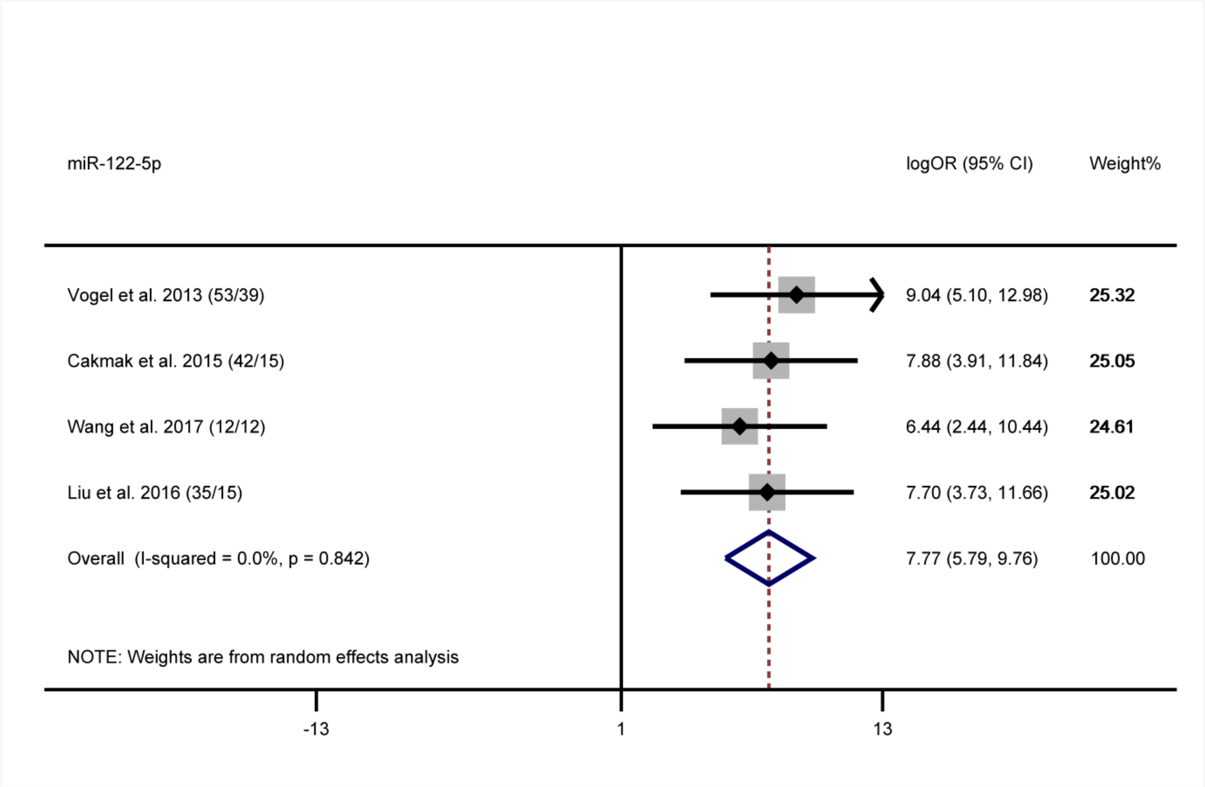

Supplementary Figure17. Forest plot of miR-122-5p

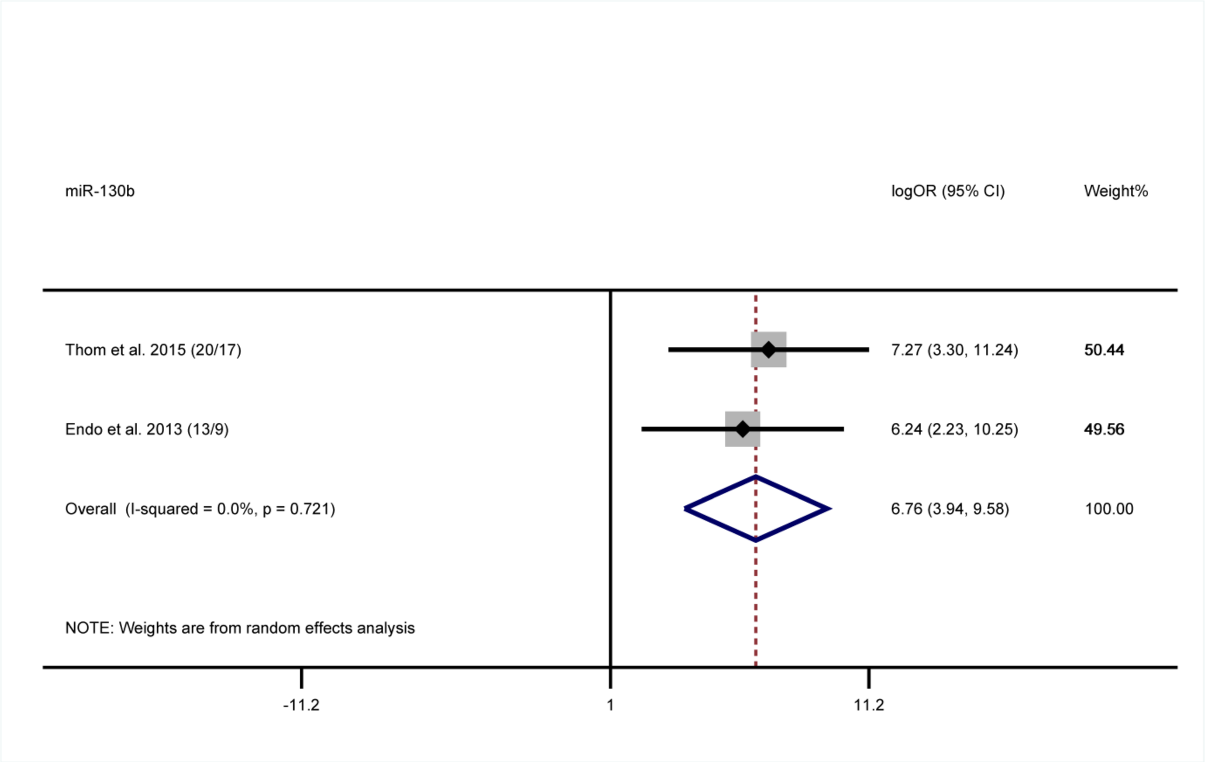

Supplementary Figure18. Forest plot of miR-130b

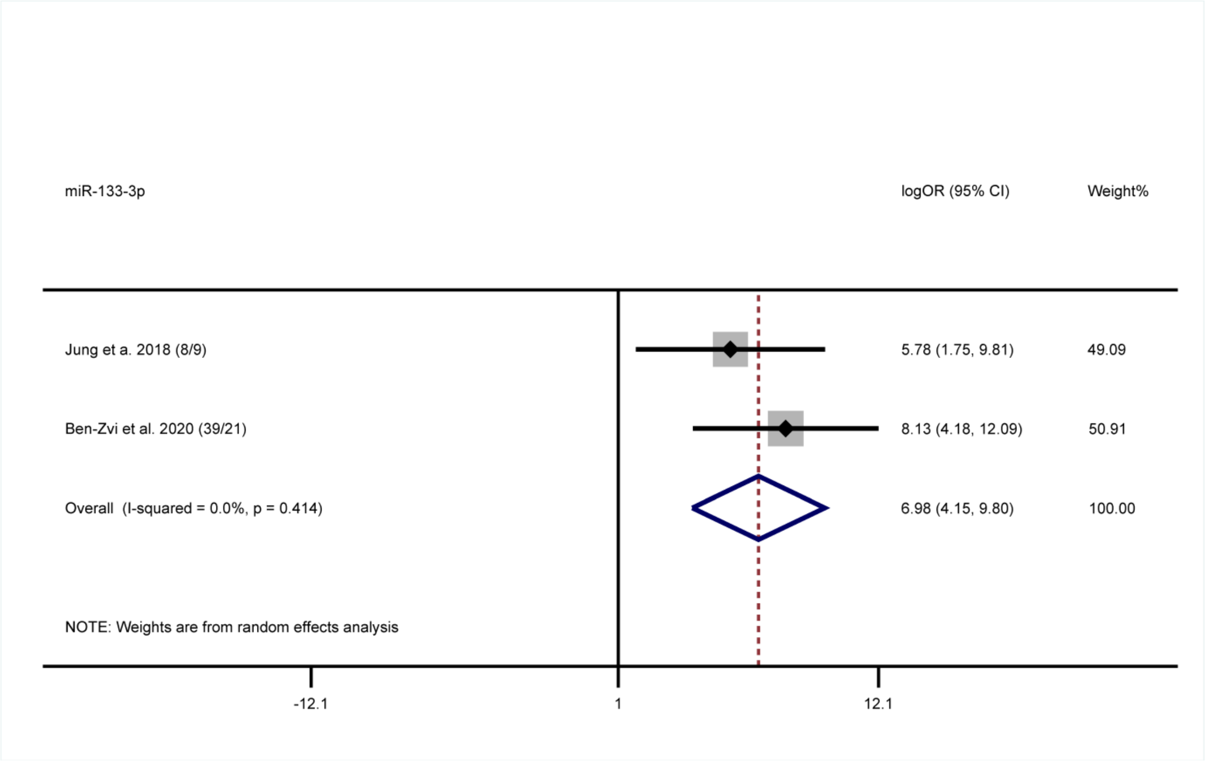

Supplementary Figure19. Forest plot of miR-133-3p

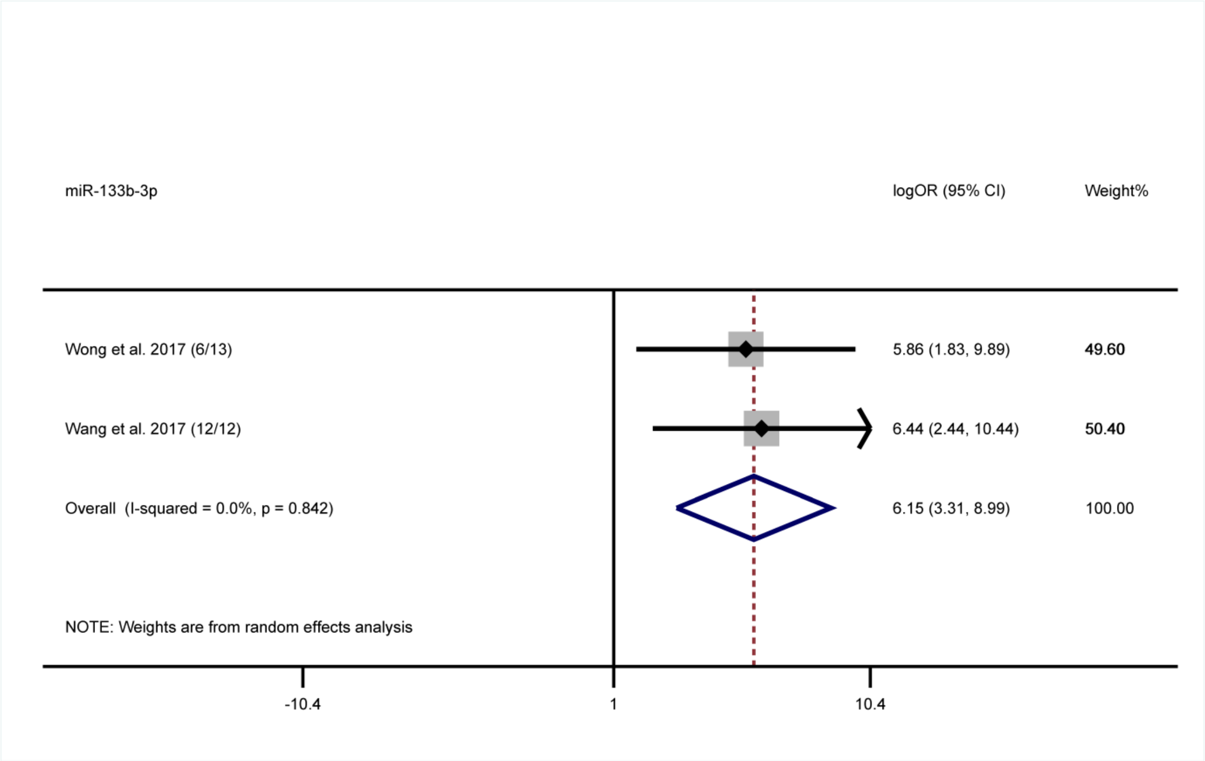

Supplementary Figure 20. Forest plot of miR-133b-3p

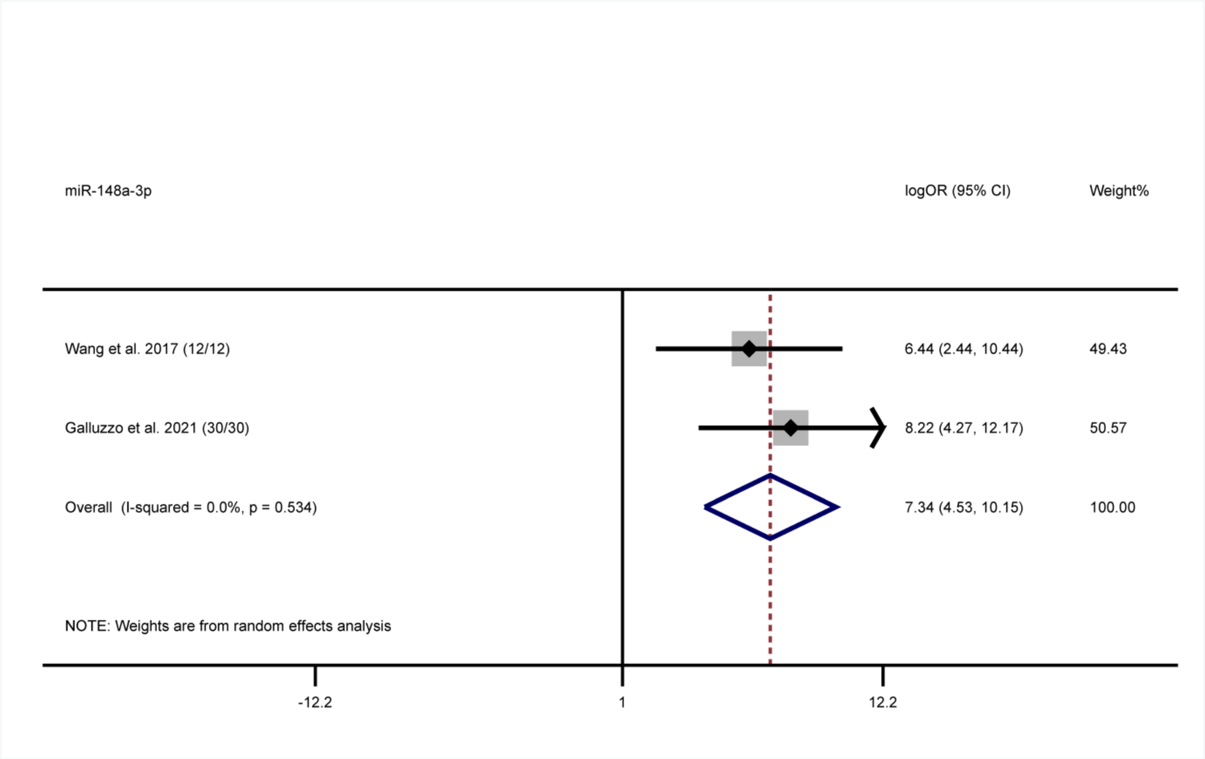

Supplementary Figure21. Forest plot of miR-148a-3p

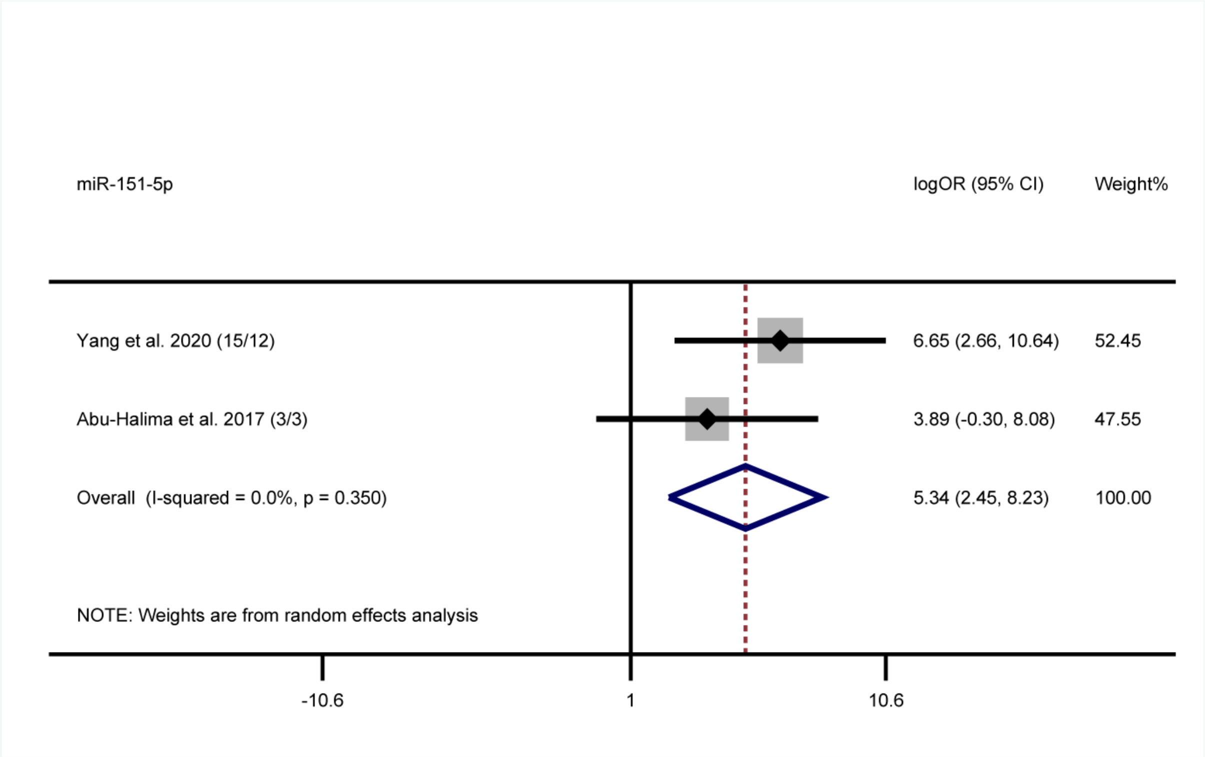

Supplementary Figure22. Forest plot of miR-151-5p

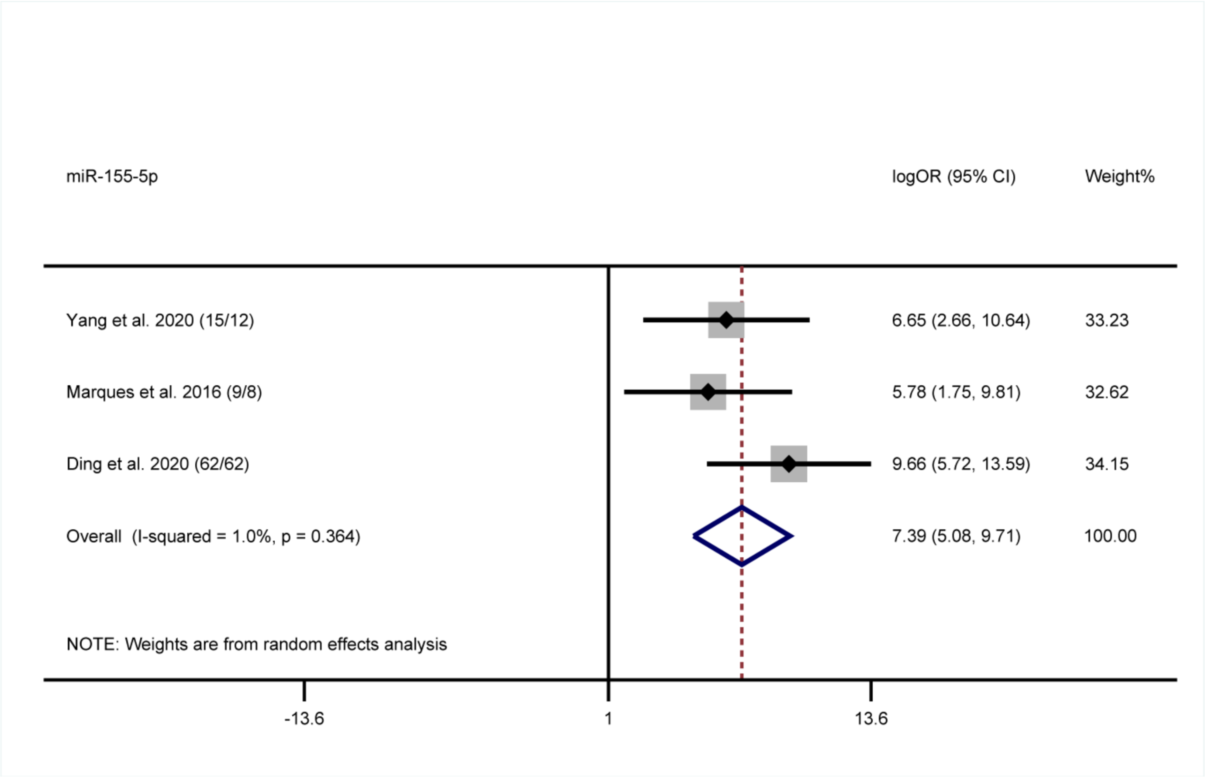

Supplementary Figure23. Forest plot of miR-155-5p

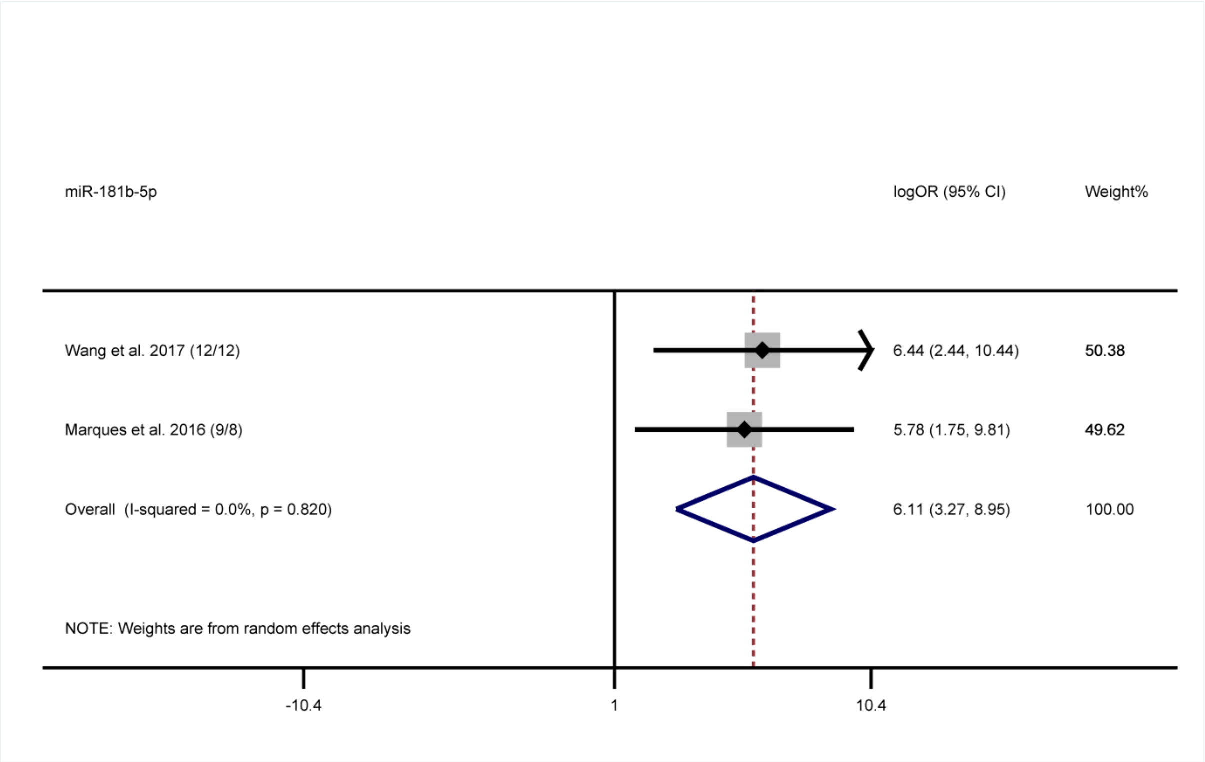

Supplementary Figure24. Forest plot of miR-181b-5p

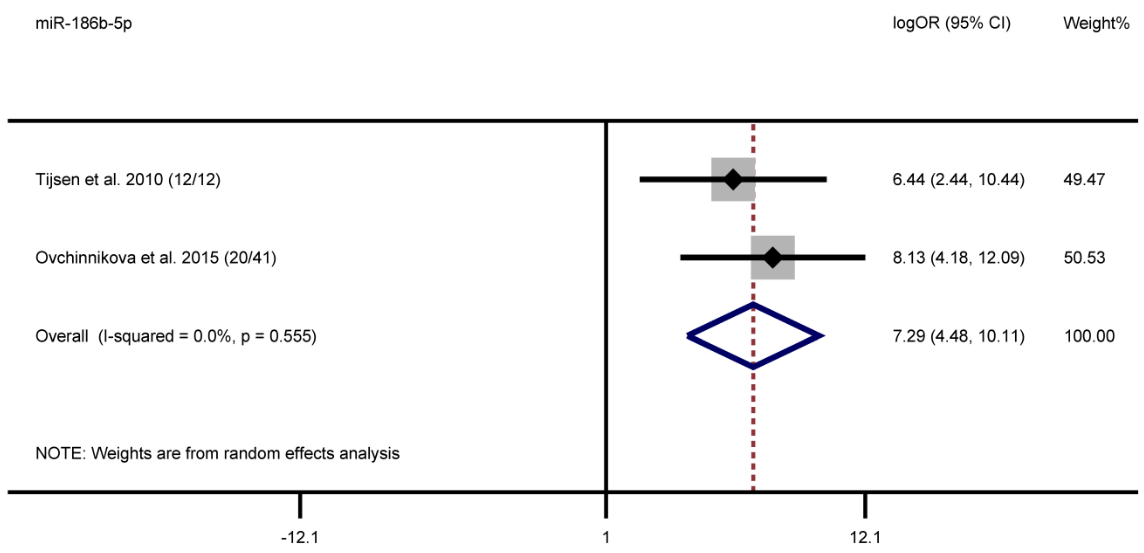

Supplementary Figure25. Forest plot of miR-186b-5p

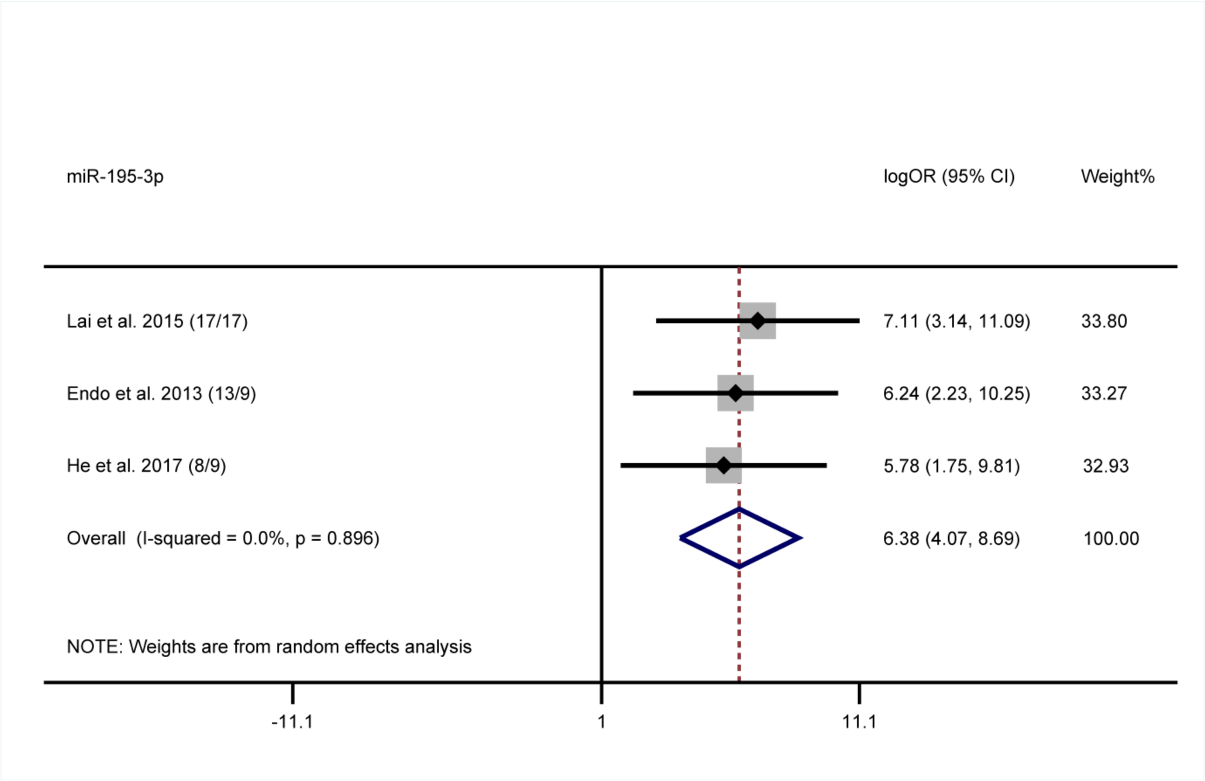

Supplementary Figure26. Forest plot of miR-195-3p

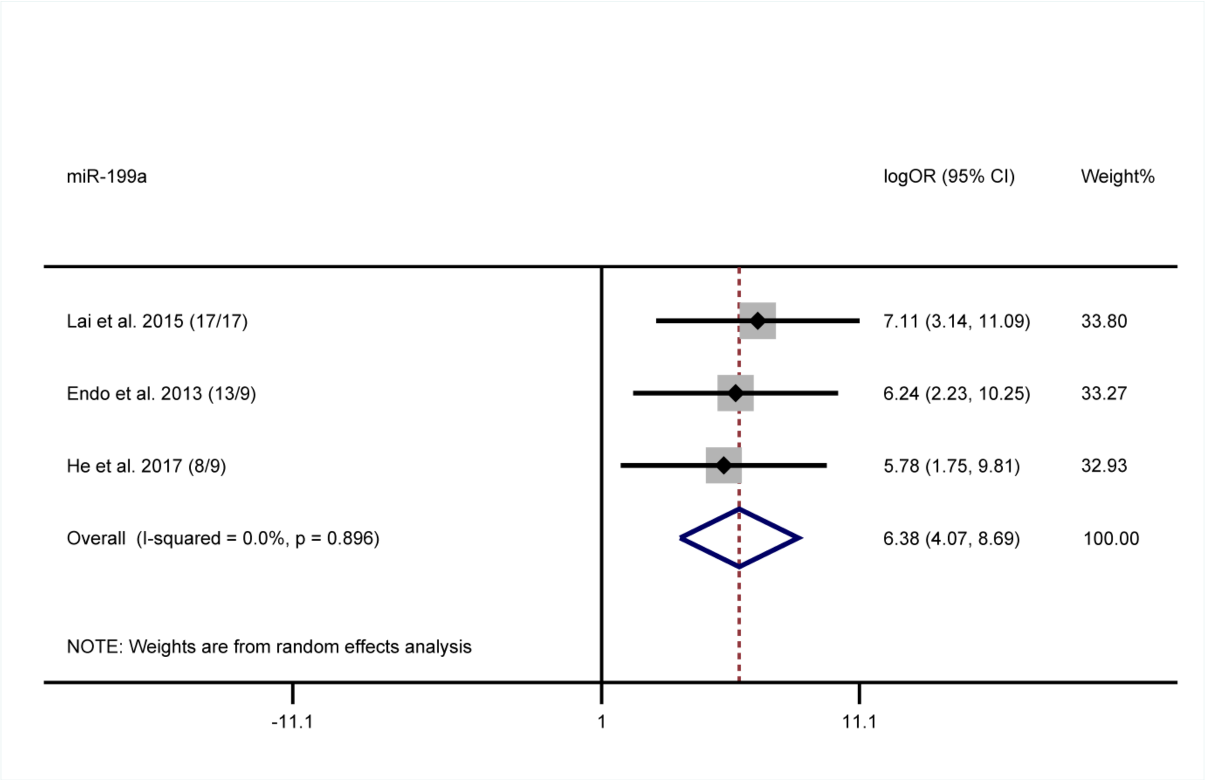

Supplementary Figure 27. Forest plot of miR-199a

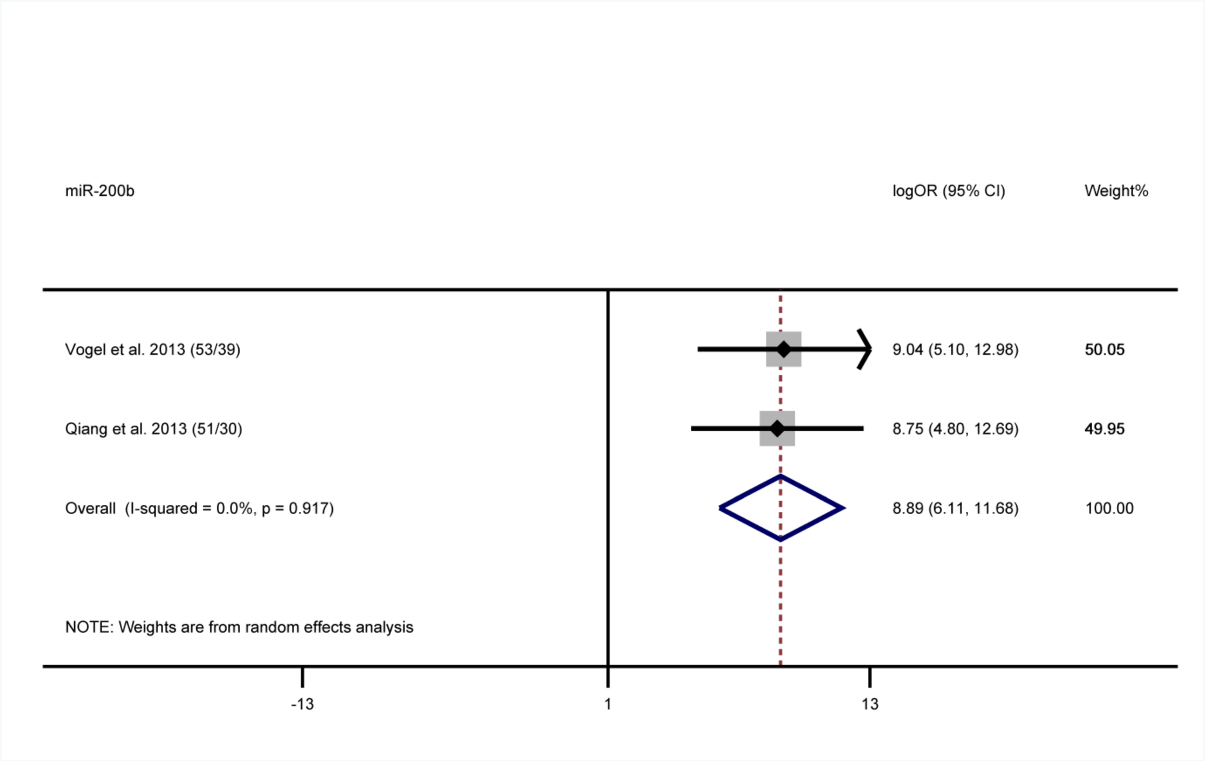

Supplementary Figure 28. Forest plot of miR-200b

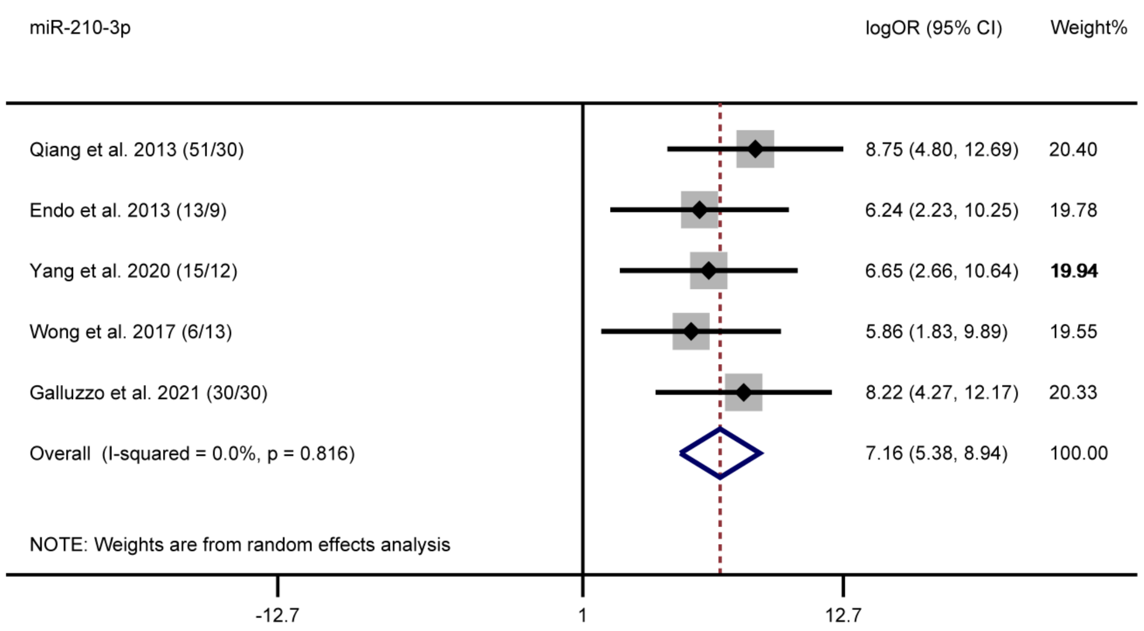

Supplementary Figure29. Forest plot of miR-210-3p

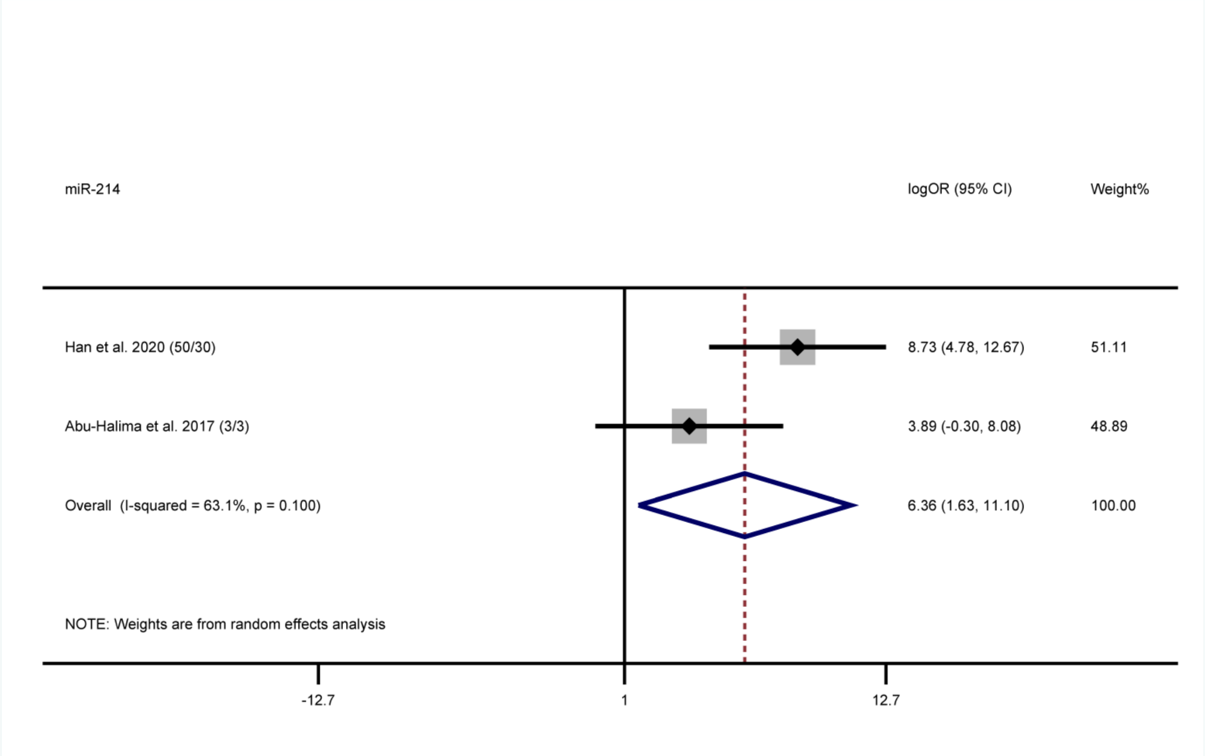

Supplementary Figure30. Forest plot of miR-214

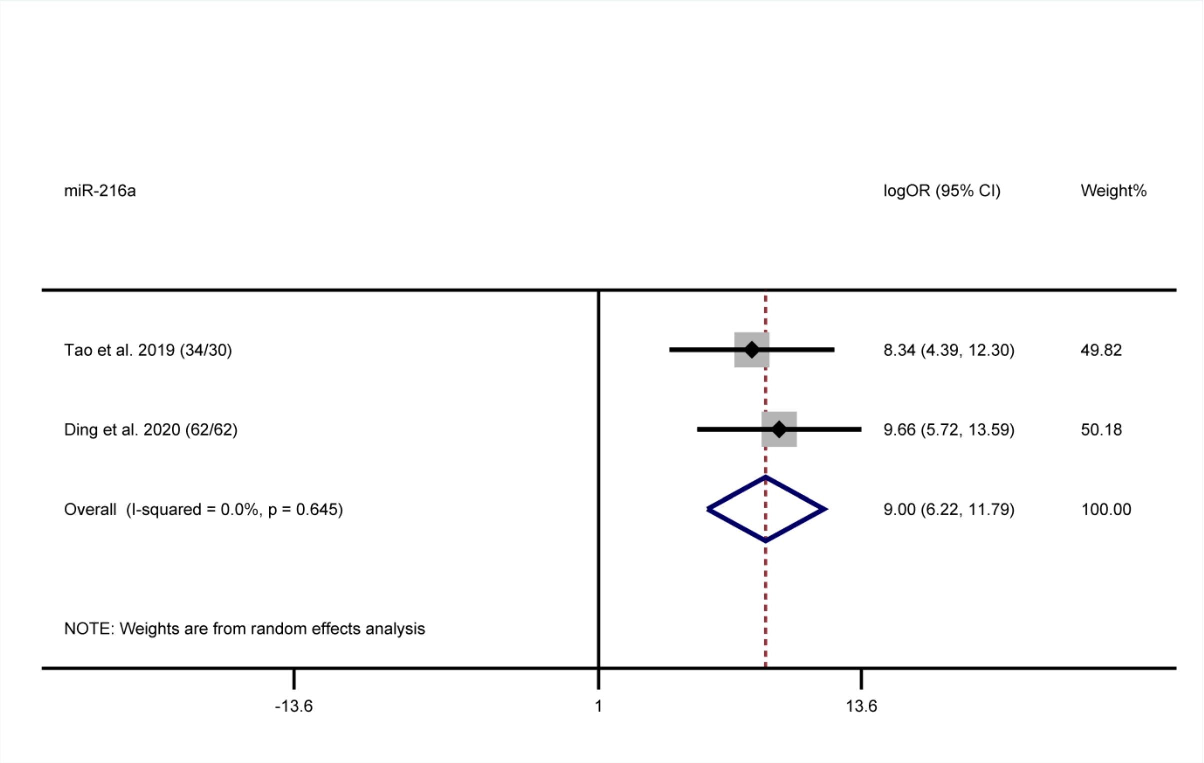

Supplementary Figure 31. Forest plot of miR-216a

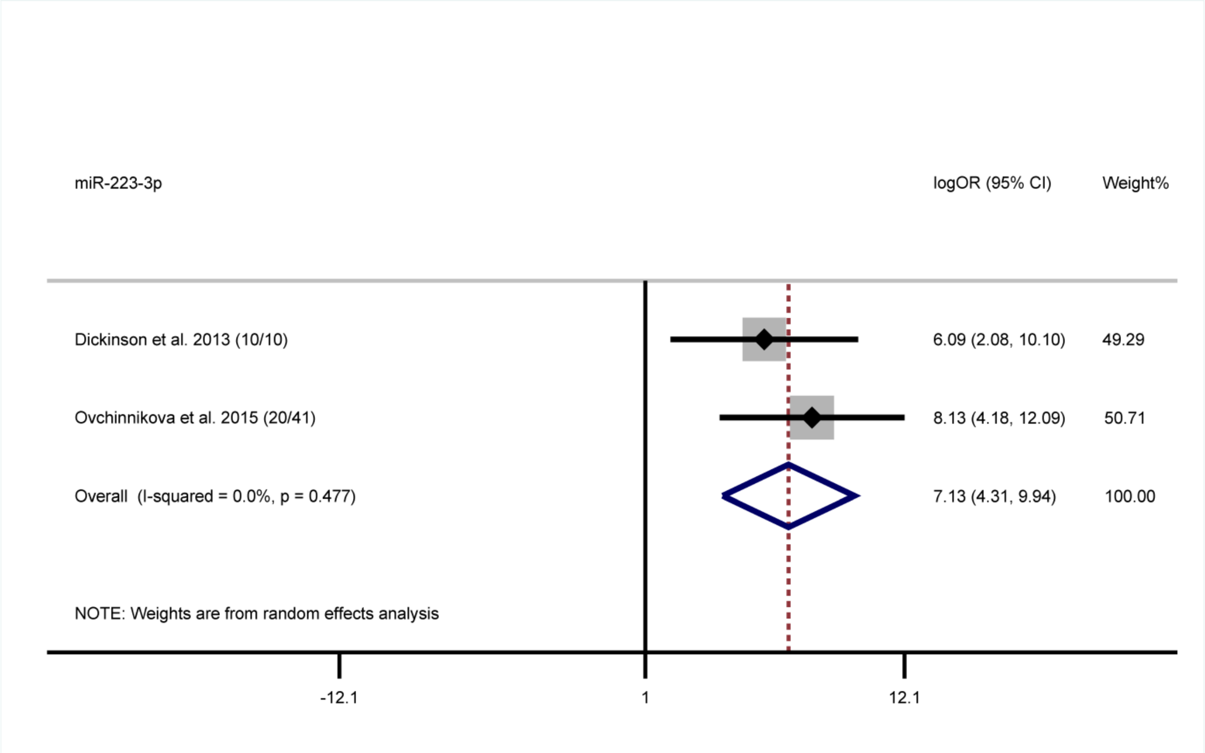

Supplementary Figure32. Forest plot of miR-223-3p

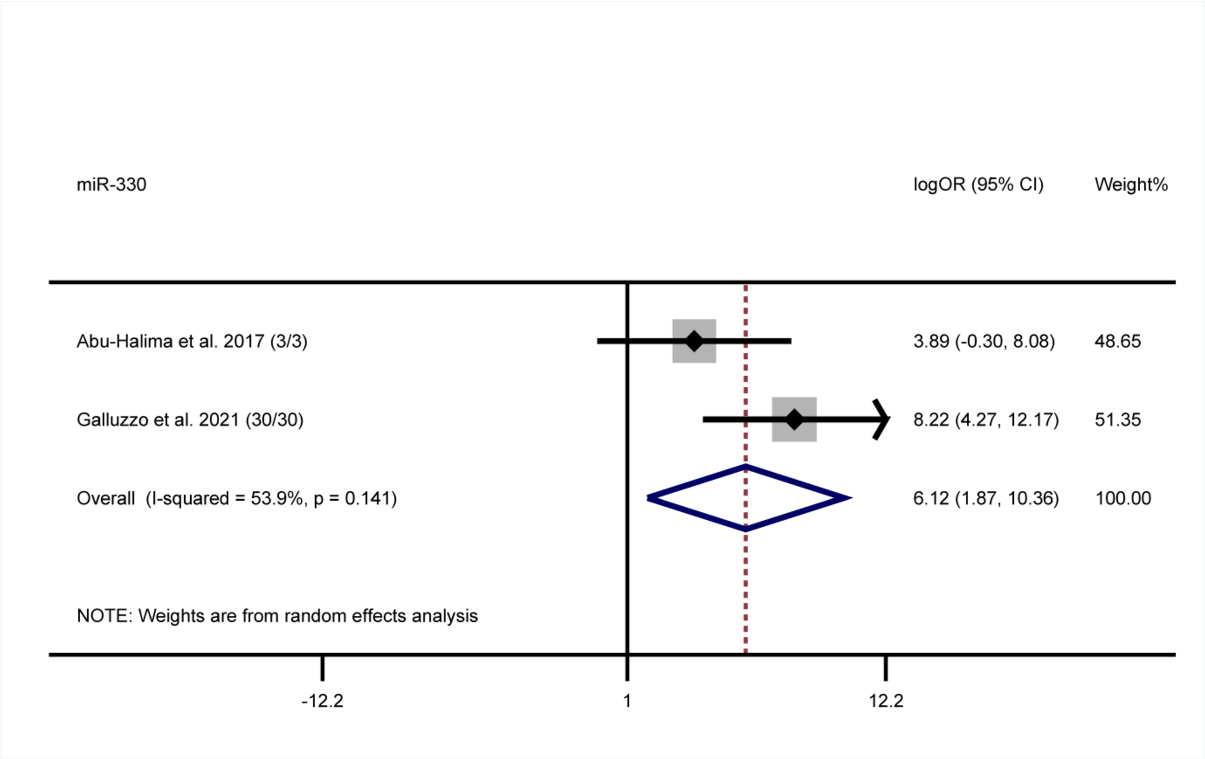

Supplementary Figure33. Forest plot of miR-330

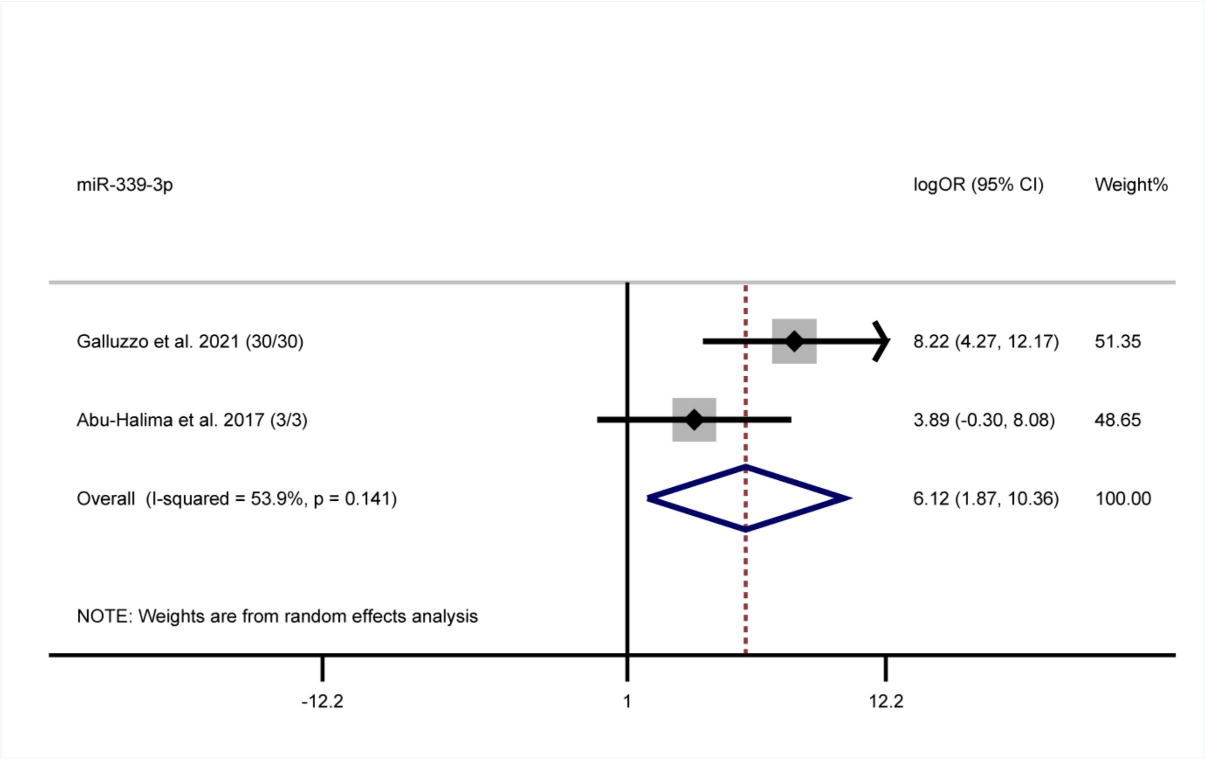

Supplementary Figure34. Forest plot of miR-339-3p

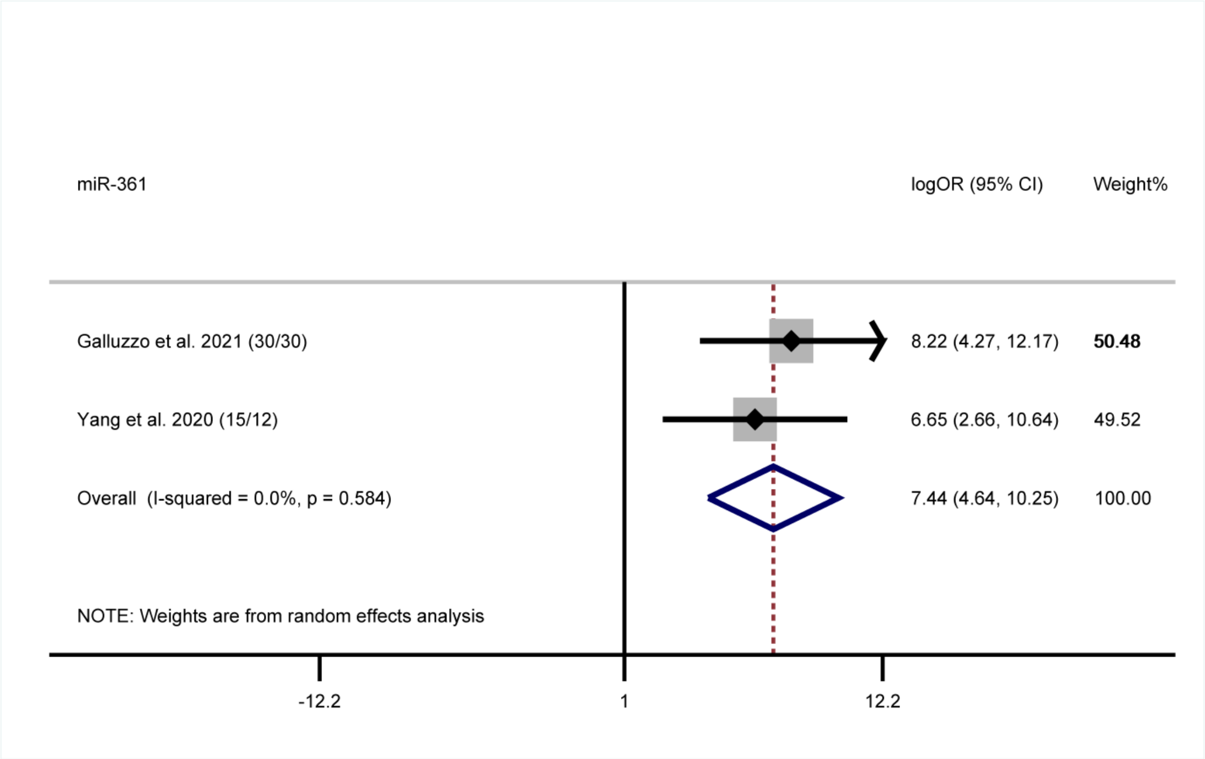

Supplementary Figure35. Forest plot of miR-361

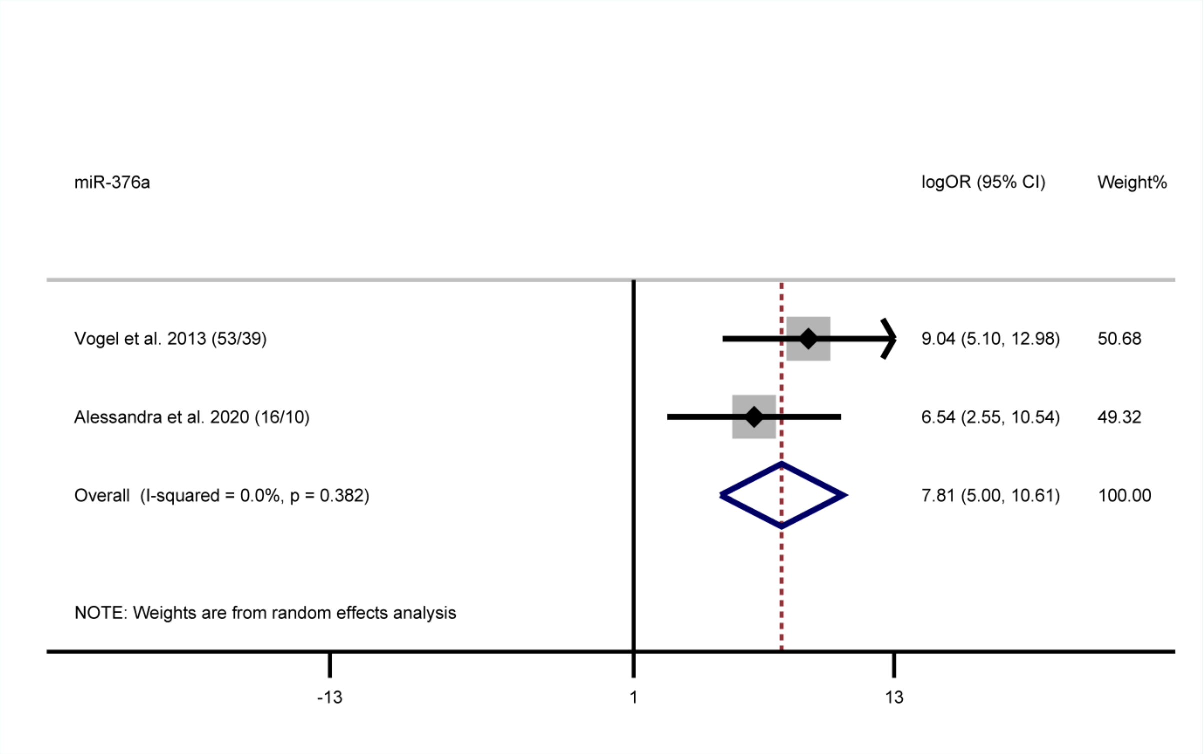

Supplementary Figure36. Forest plot of miR-376a

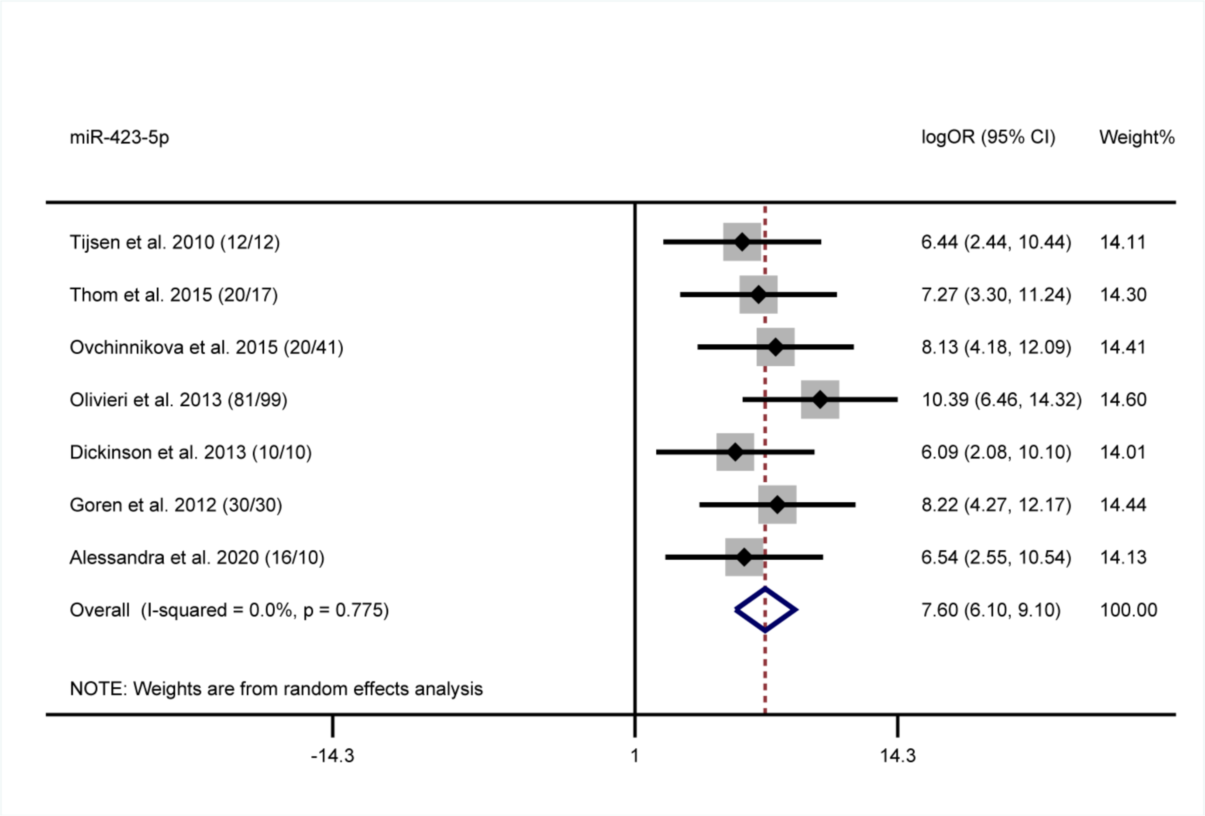

Supplementary Figure37. Forest plot of miR-423-5p

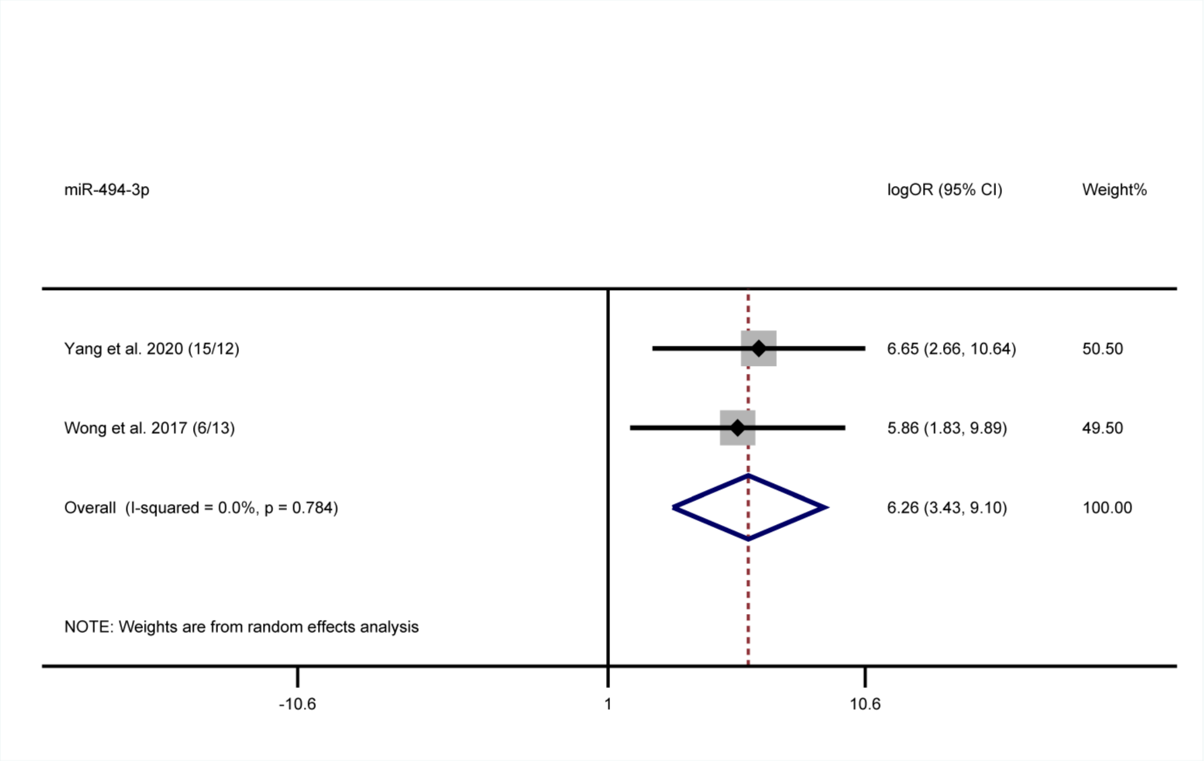

Supplementary Figure38. Forest plot of miR-494-3p

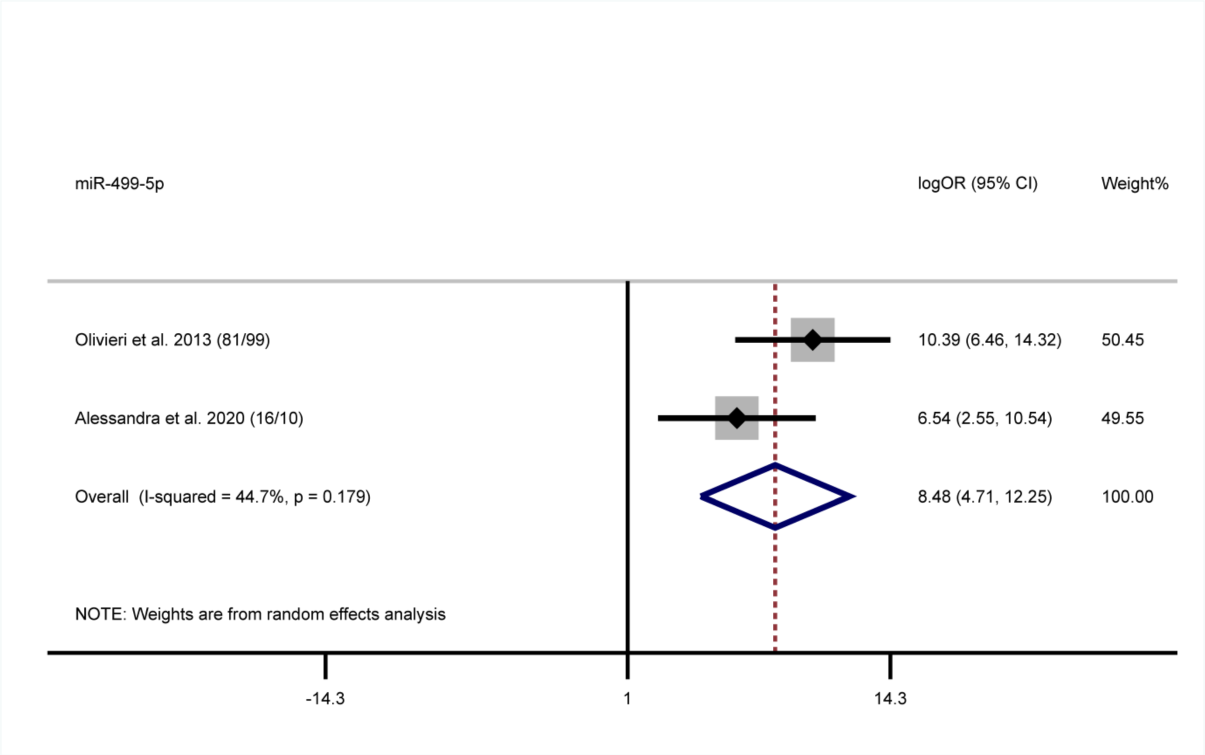

Supplementary Figure39. Forest plot of miR-499-5p

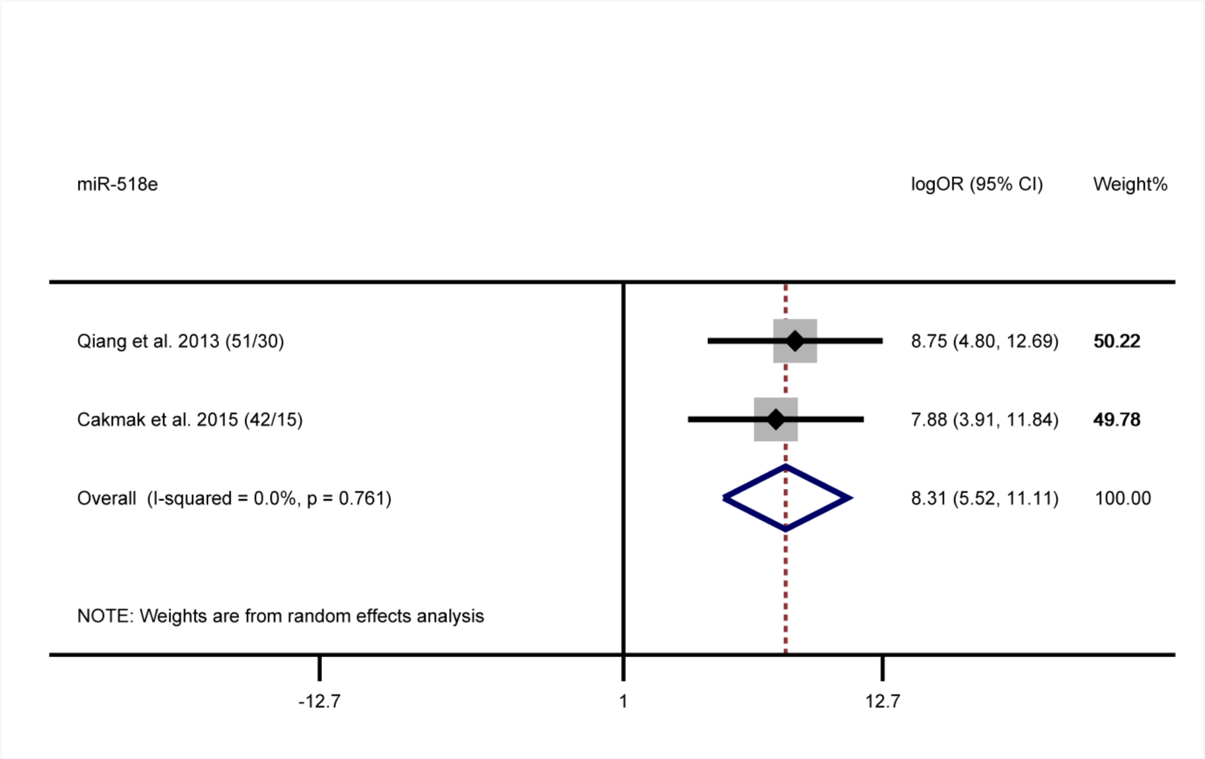

Supplementary Figure40. Forest plot of miR-518e

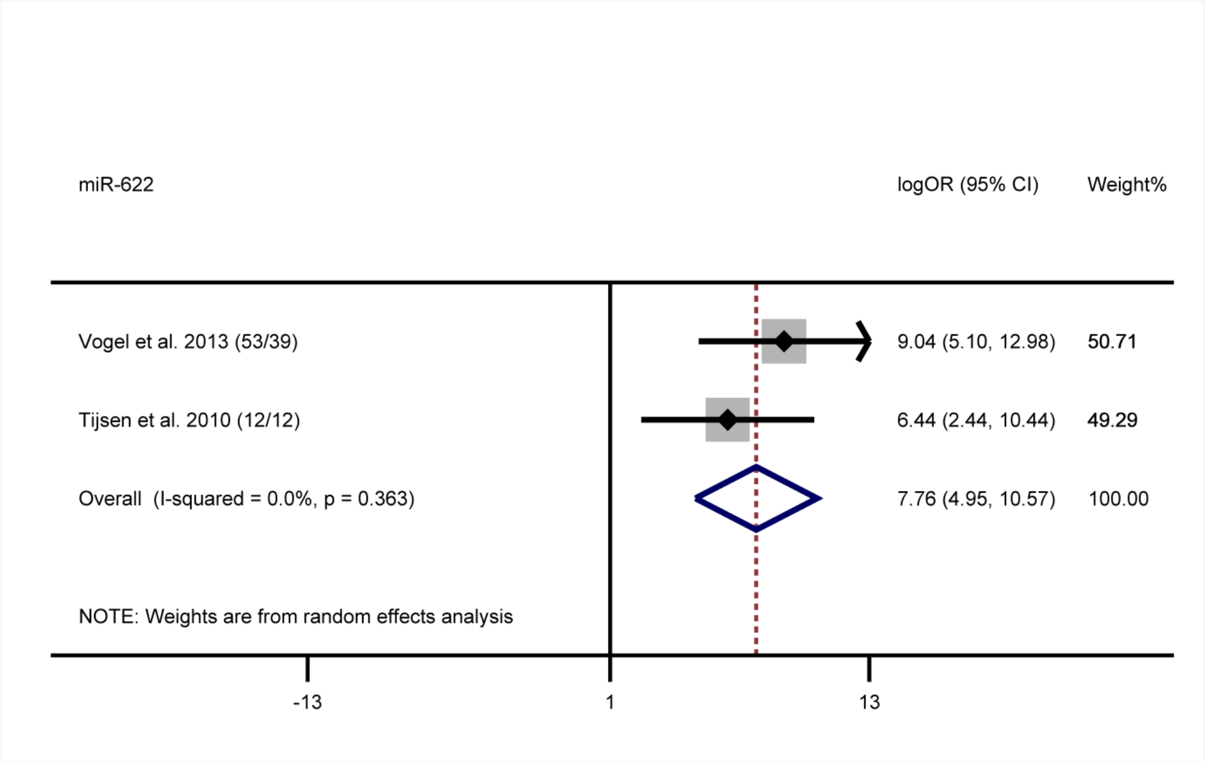

Supplementary Figure41. Forest plot of miR-622

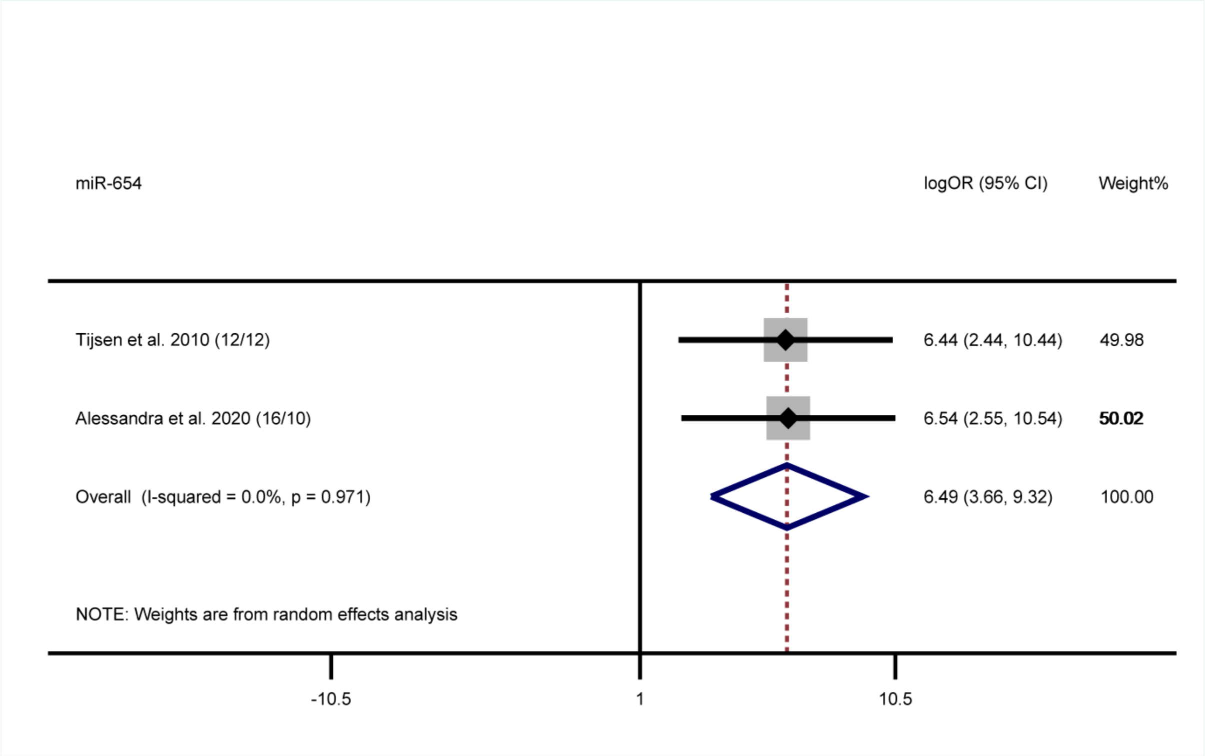

Supplementary Figure42. Forest plot of miR-654

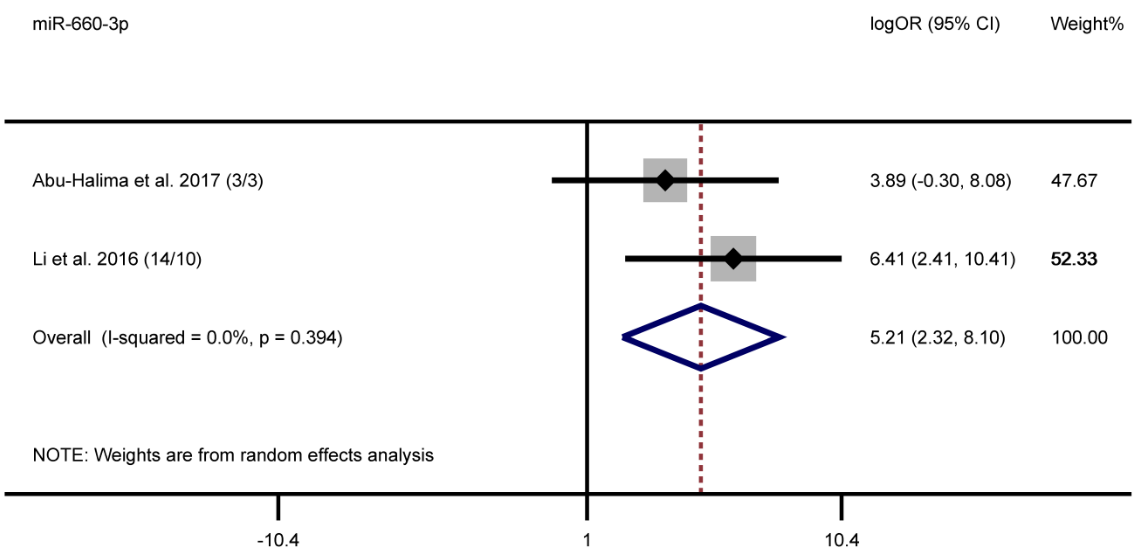

Supplementary Figure43. Forest plot of miR-660-3p

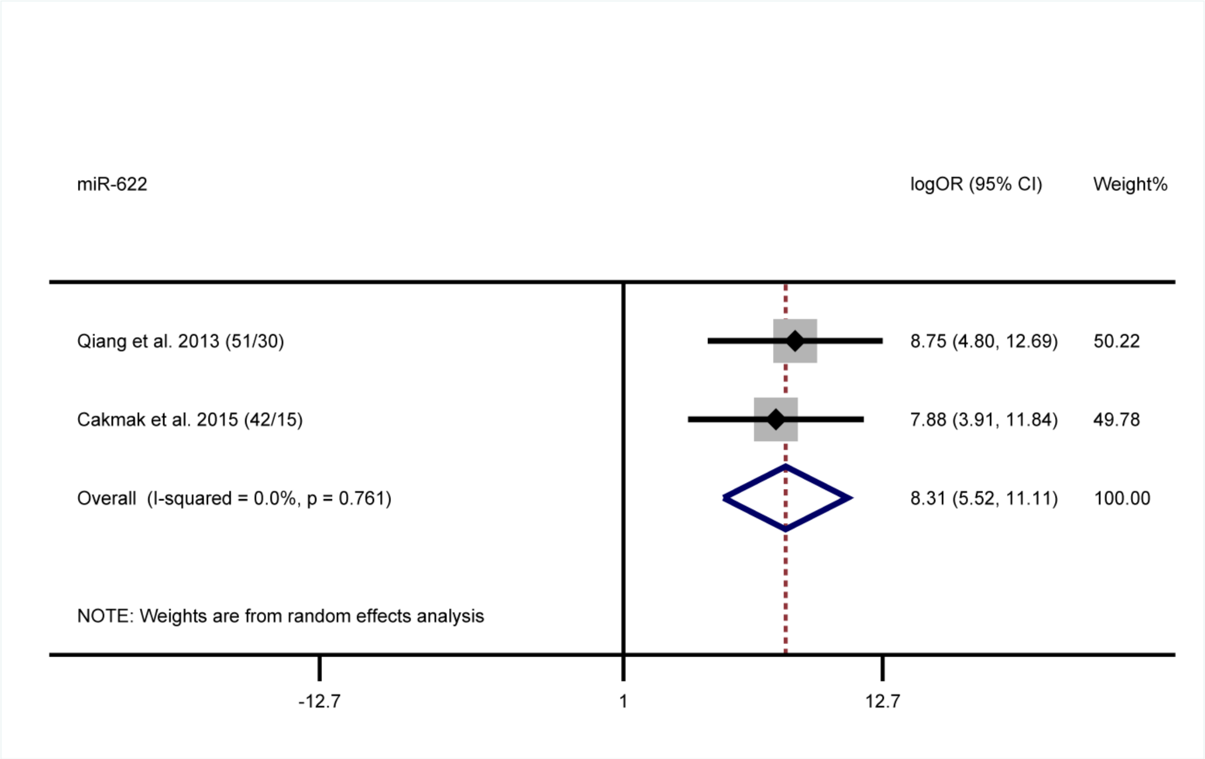

Supplementary Figure44. Forest plot of miR-662

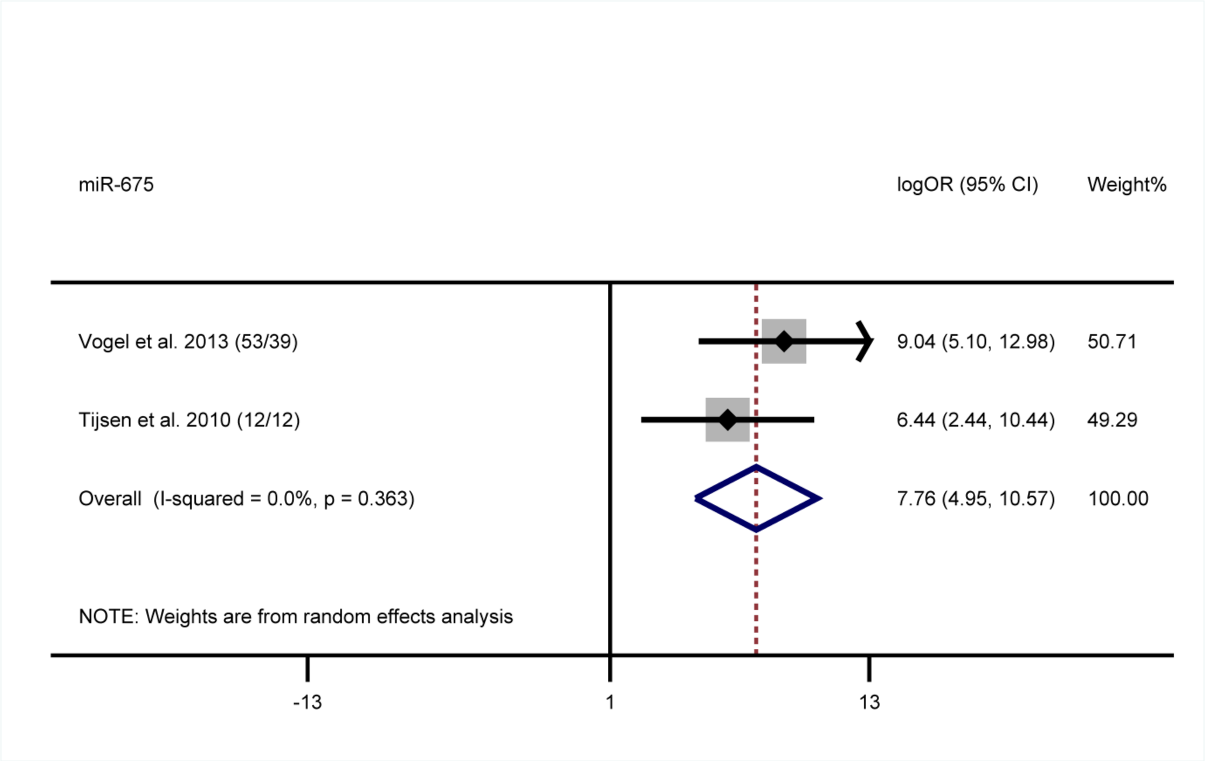

Supplementary Figure45. Forest plot of miR-675

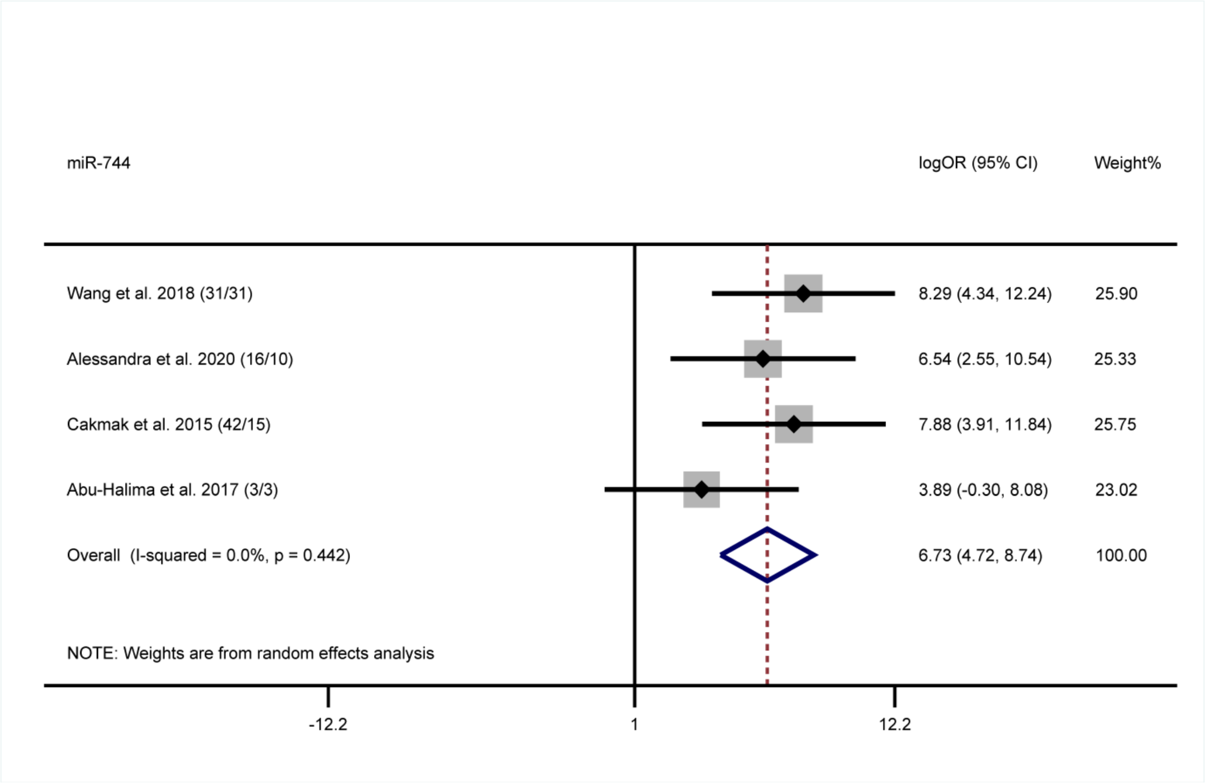

Supplementary Figure46. Forest plot of miR-744

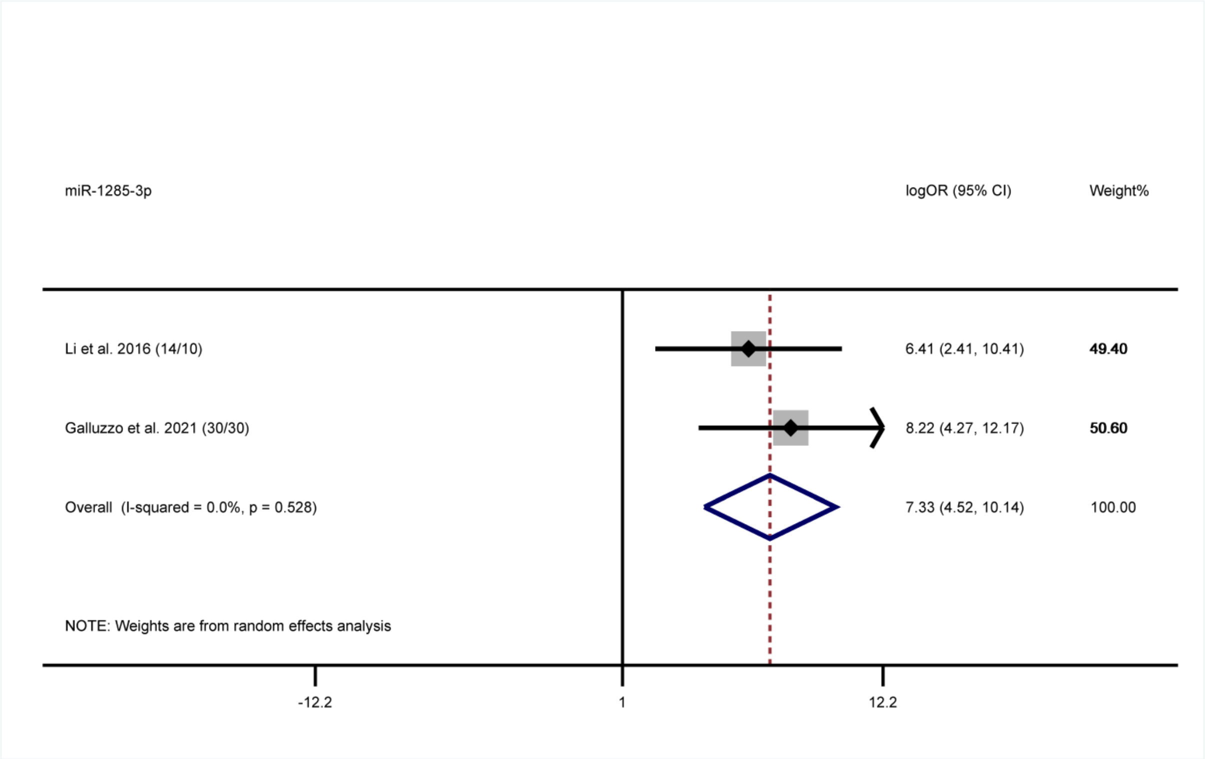

Supplementary Figure47. Forest plot of miR-1285-3p

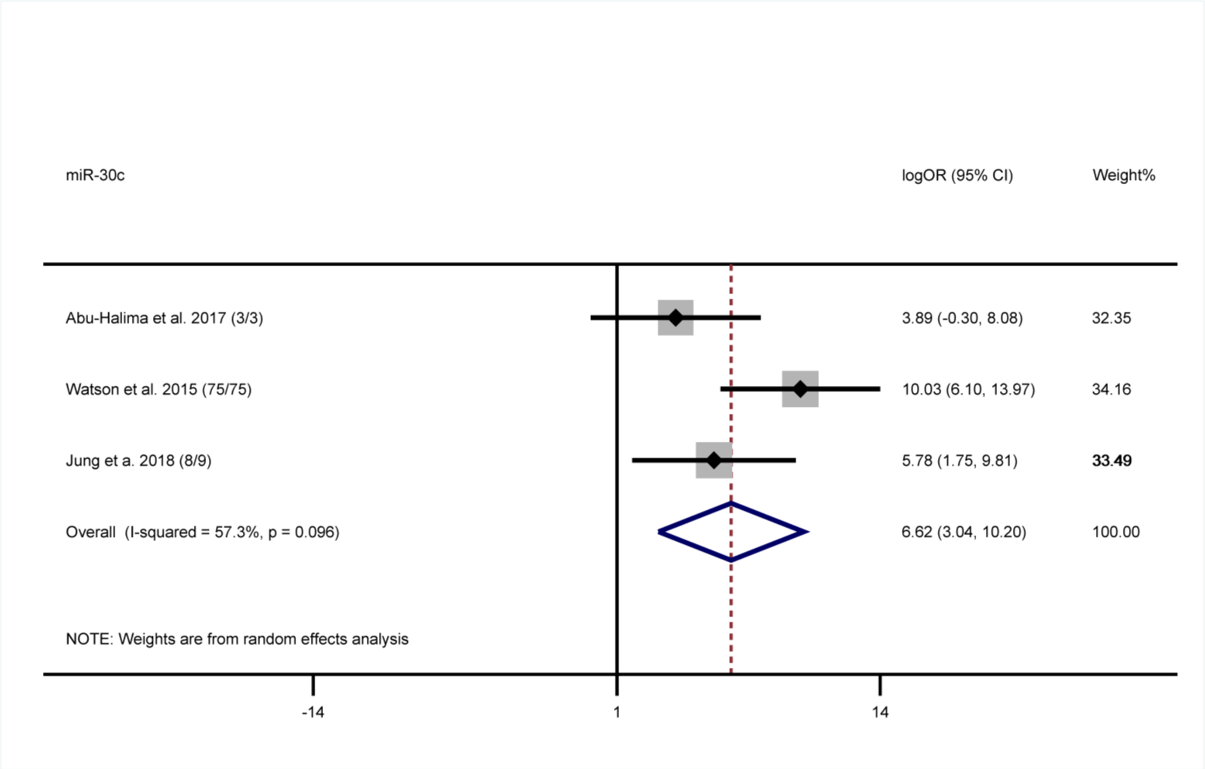

Supplementary Figure48. Forest plot of miR-30c

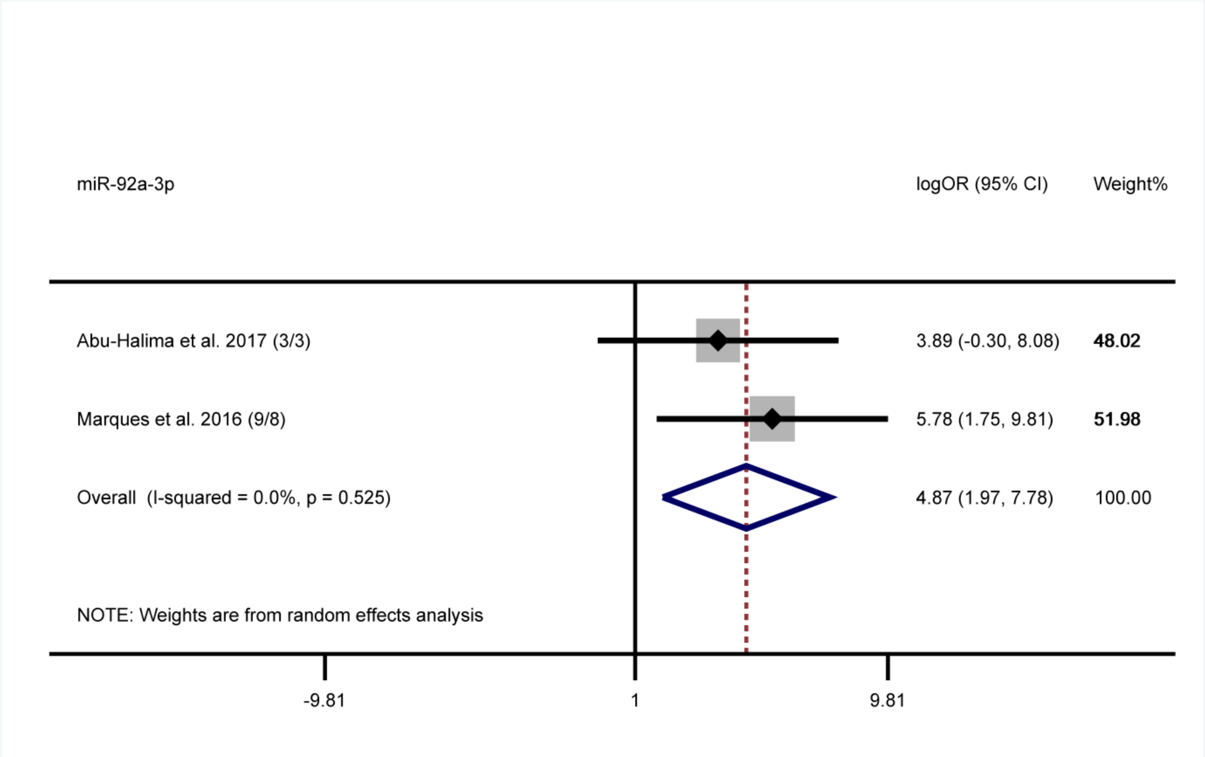

Supplementary Figure49. Forest plot of miR-92a-3p

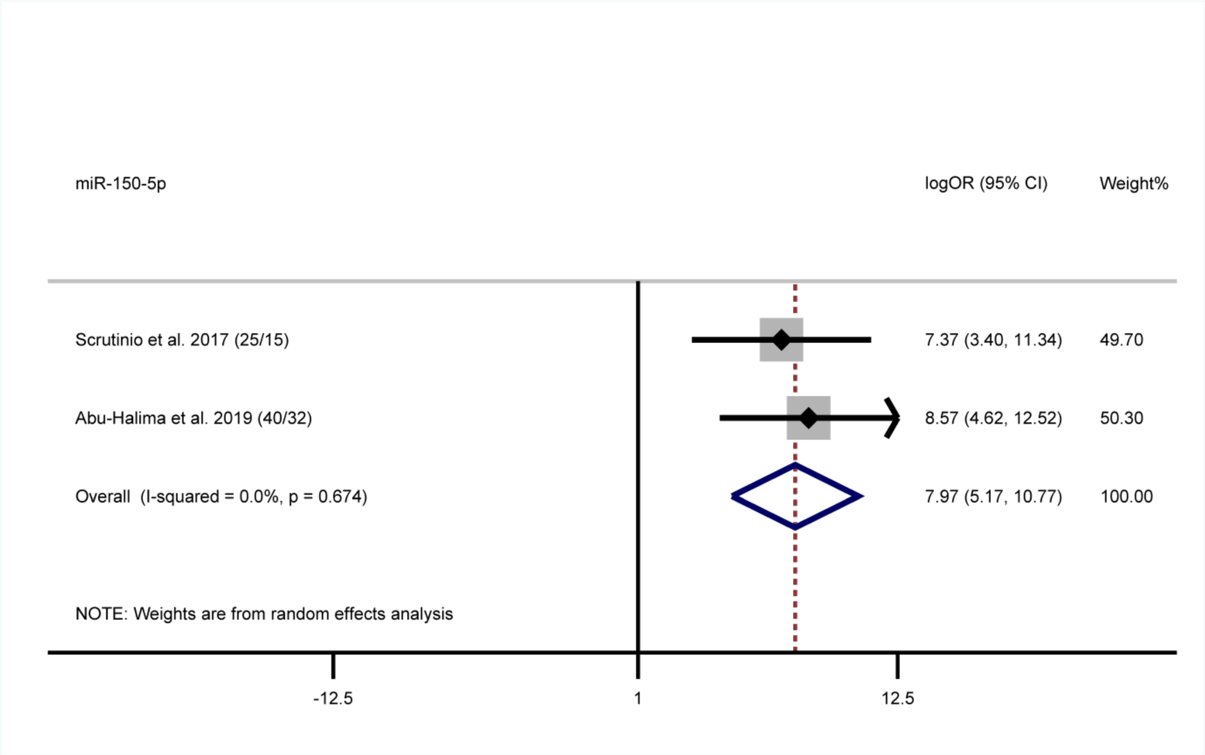

Supplementary Figure50. Forest plot of miR-150-5p

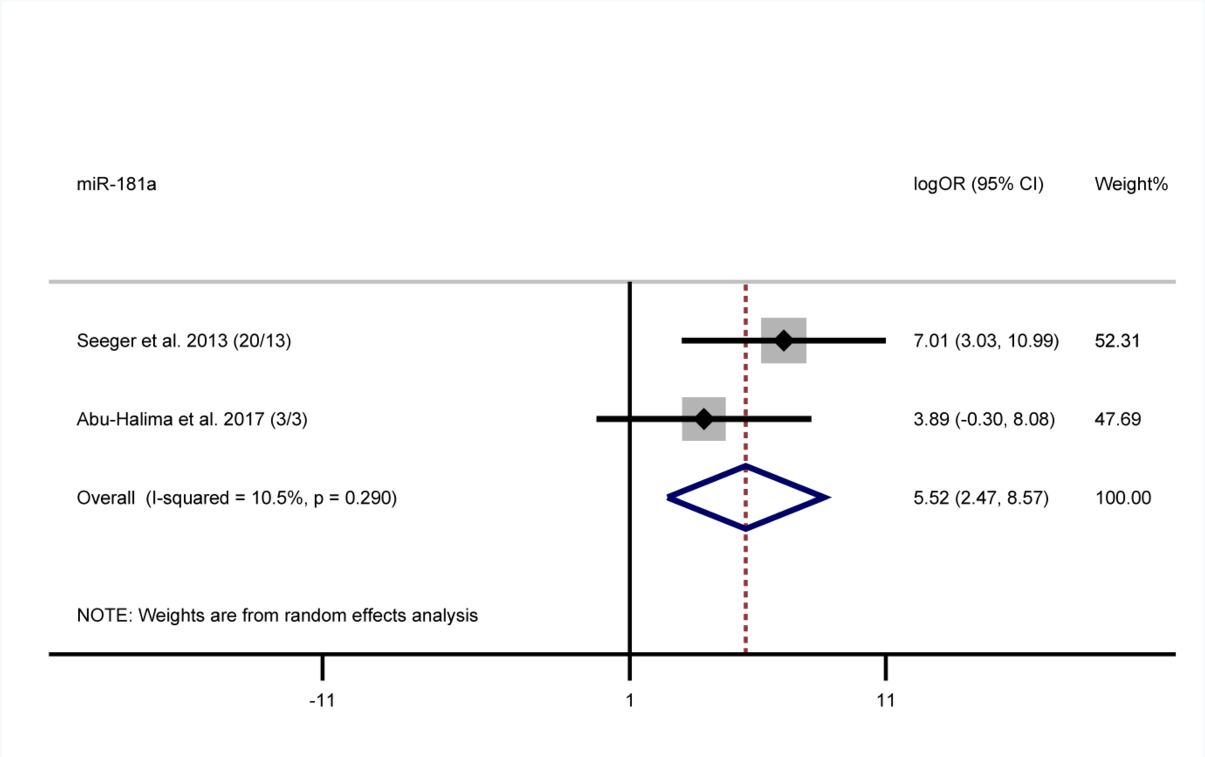

Supplementary Figure51. Forest plot of miR-181a

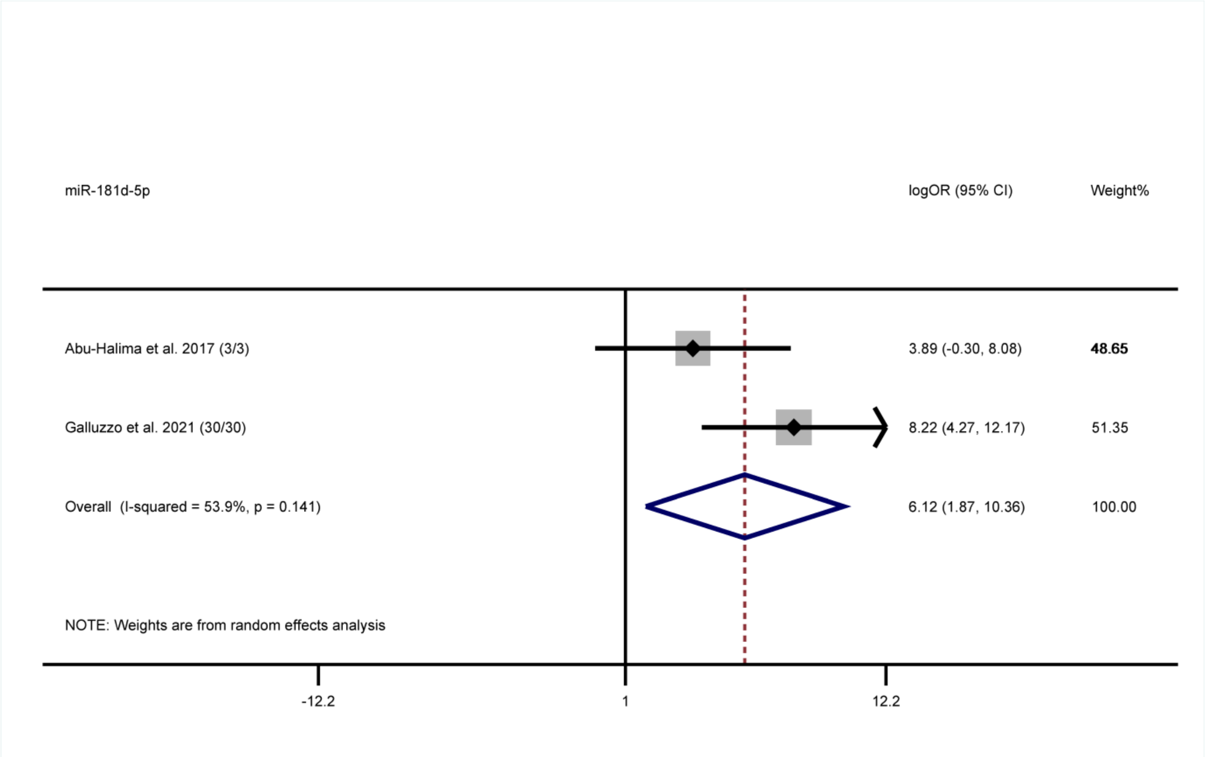

Supplementary Figure52. Forest plot of miR-181d-5p

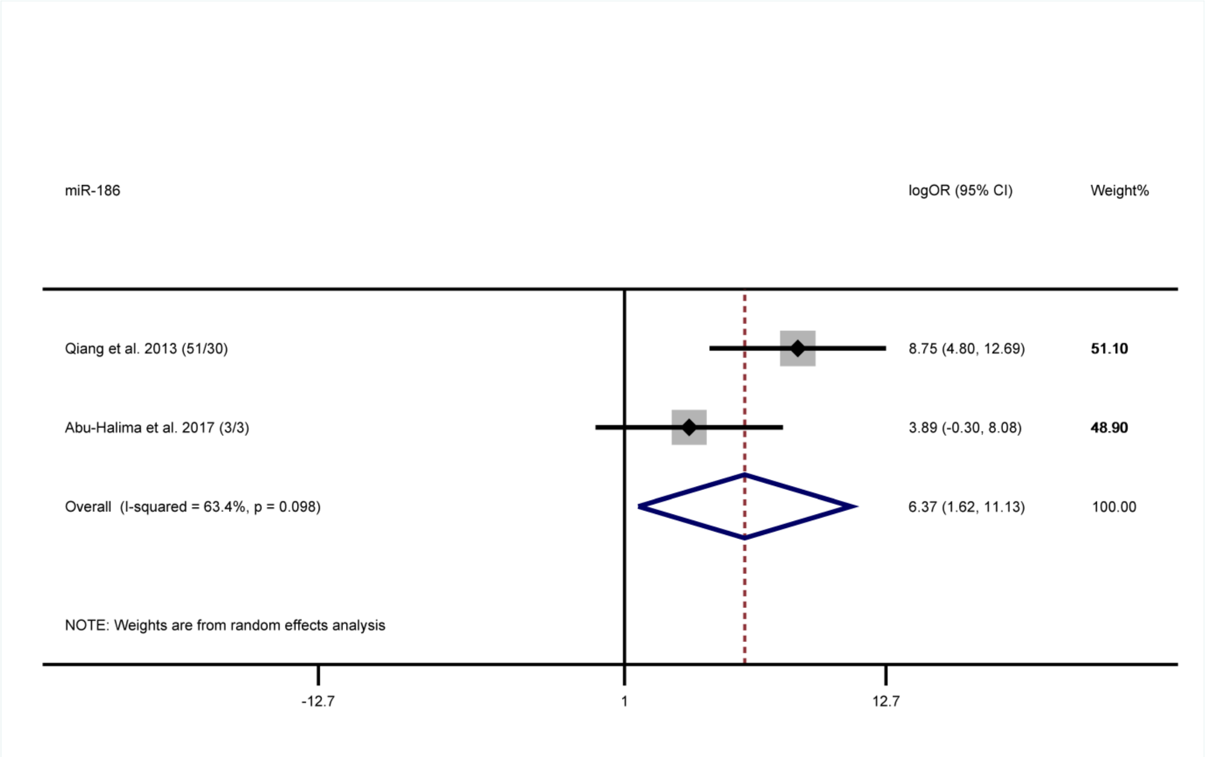

Supplementary Figure53. Forest plot of miR-186

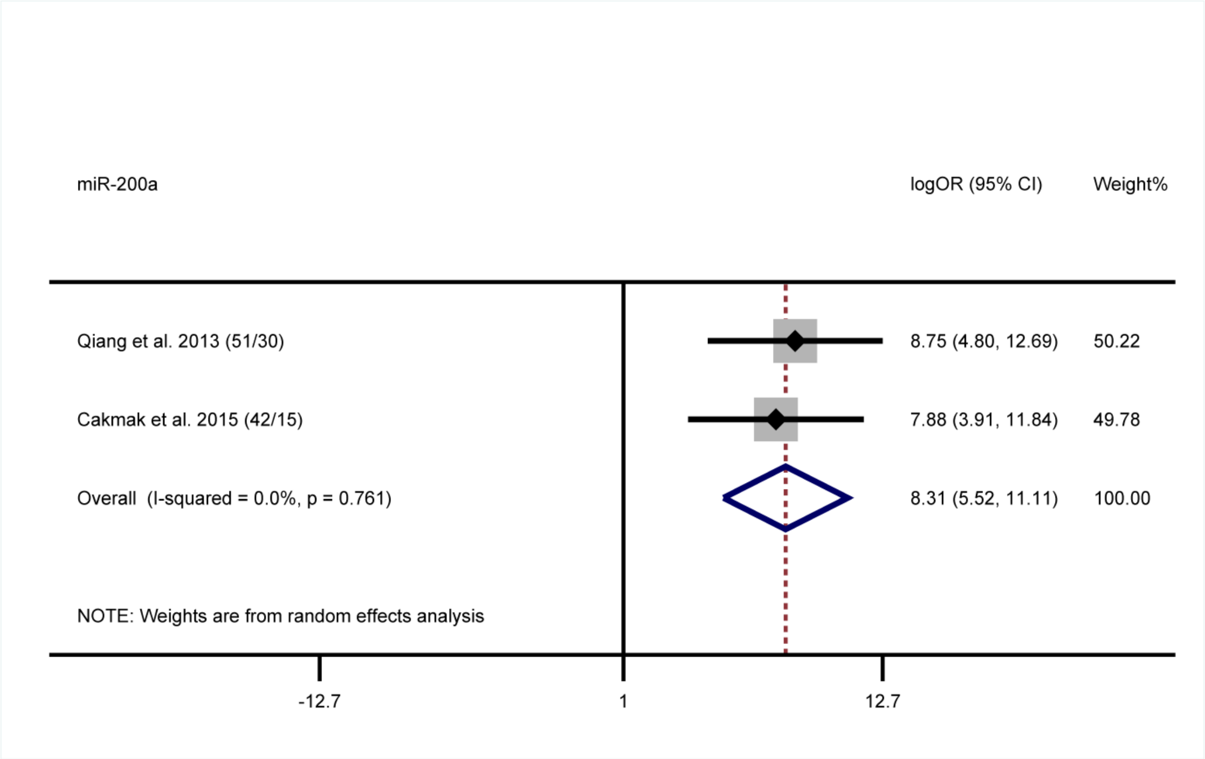

Supplementary Figure54. Forest plot of miR-200a

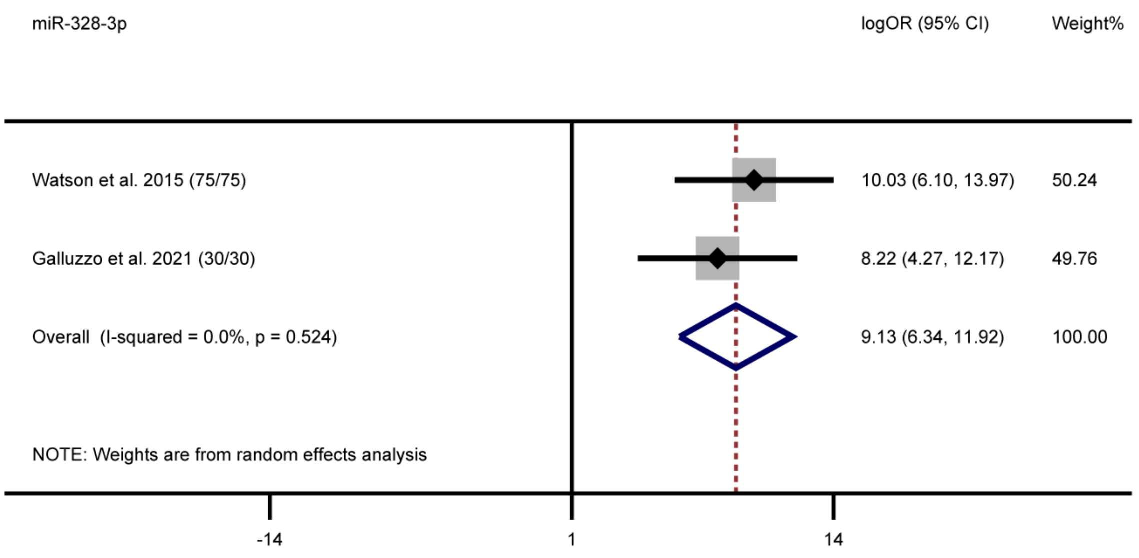

Supplementary Figure55. Forest plot of miR-328-3p

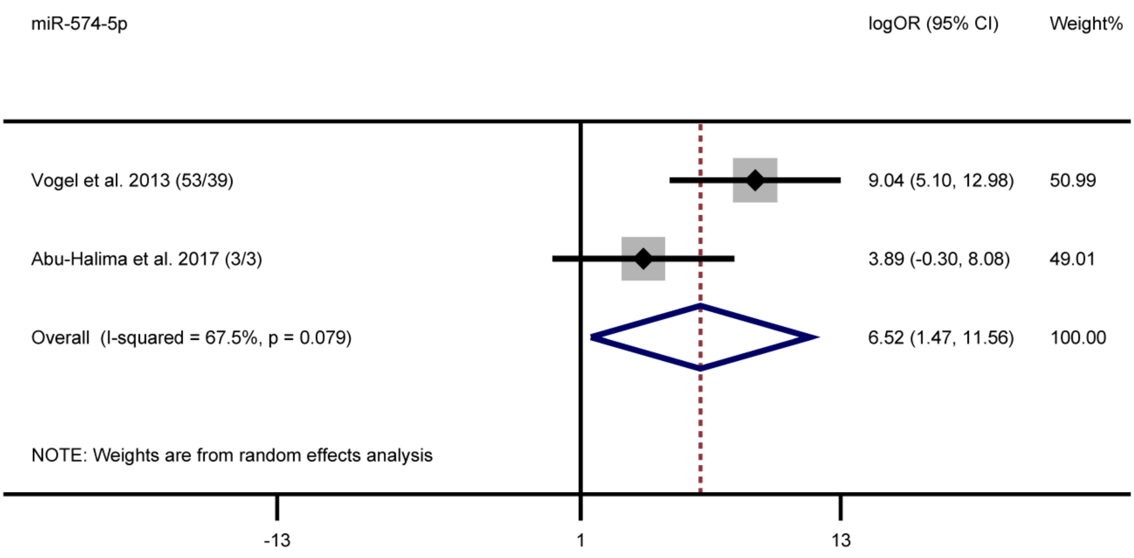

Supplementary Figure56. Forest plot of miR-574-5p

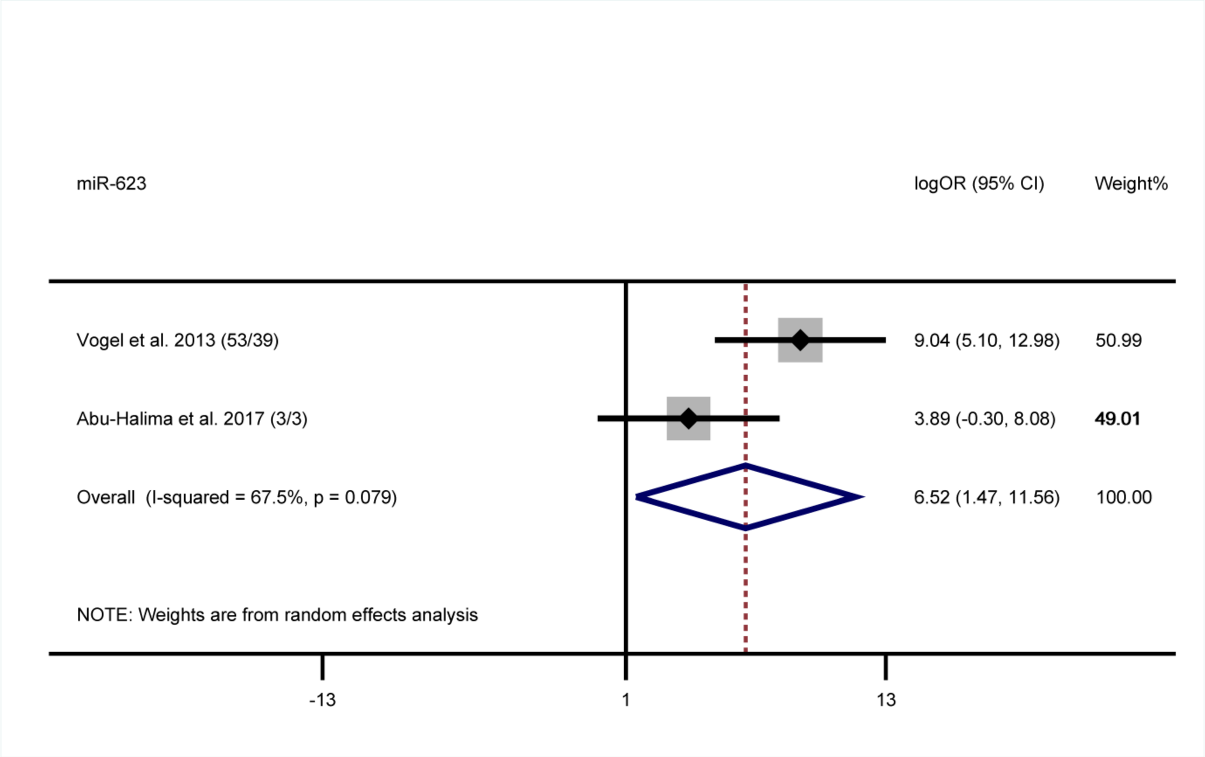

Supplementary Figure57. Forest plot of miR-623

References

1. Wang N, Zhou Z, Liao X, Zhang T. Role of microRNAs in cardiac hypertrophy and heart failure. *IUBMB life* (2009) 61(6):566-71. Epub 2009/05/28. doi: 10.1002/iub.204. PubMed PMID: 19472179.
2. Silva FCD, Iop RDR, Andrade A, Costa VP, Gutierres Filho PJB, Silva RD. Effects of Physical Exercise on the Expression of MicroRNAs: A Systematic Review. *Journal of strength and conditioning research* (2020) 34(1):270-80. Epub 2019/12/27. doi: 10.1519/jsc.0000000000003103. PubMed PMID: 31877120.
3. Paseban M, Marjaneh RM, Banach M, Riahi MM, Bo S, Sahebkar A. Modulation of microRNAs by aspirin in cardiovascular disease. *PloS one* (2020) 30(5):249-54. Epub 2018/04/27. doi: 10.1371/journal.pone.0196697
10.1016/j.tcm.2019.08.005. PubMed PMID: 31444100; PubMed Central PMCID: PMCPMC5919659 manuscript have the following competing interests: Drs. Newton-Cheh and Wu are listed as co-inventors on patents for the therapeutic targeting of microRNAs as potential treatments for hypertension or heart failure (WO 2013188787 A1; Inhibitors of micrornas that regulate production of atrial natriuretic peptide (anp) as therapeutics and uses thereof, WO 2016077574 A1; Inhibitors of micrornas mir-155, mir-103, mir-105 and mir-107 that regulate production of atrial natriuretic peptide (anp) as therapeutics and uses thereof). These patents do not alter our adherence to PLOS ONE policies on sharing data and materials.
4. Masson S, Batkai S, Beermann J, Bar C, Pfanne A, Thum S, et al. Circulating microRNA-132 levels improve risk prediction for heart failure hospitalization in patients with chronic heart failure. *European journal of heart failure* (2017) (no pagination). doi: 10.1002/ejhf.961. PubMed PMID: CN-01421232.
5. Marketou ME, Kontaraki JE, Maragkoudakis S, Patrianakos A, Konstantinou J, Nakou H, et al. MicroRNAs in Peripheral Mononuclear Cells as Potential Biomarkers in Hypertensive Patients With Heart Failure With Preserved Ejection Fraction. *American journal of hypertension* (2018) 31(6):651-7. Epub 2018/03/06. doi: 10.1093/ajh/hpy035. PubMed PMID: 29506053.
6. Liu Q, Li H, Wang N, Chen H, Wang J. Expression of miR-126 and miR-508-5p in endothelial progenitor cells is associated with the prognosis of chronic heart failure patients. *International journal of cardiology* (2013) 168(3):2082-8. doi: 10.1016/j.ijcard.2013.01.160.
7. Lin X, Zhang S, Huo Z. Serum Circulating miR-150 is a Predictor of Post-Acute Myocardial Infarction Heart Failure. *International heart journal* (2019) 60(2):280-6. Epub 2019/02/13. doi: 10.1536/ihj.18-306. PubMed PMID: 30745540.
8. Lin B, Feng DG, Xu J. microRNA-665 silencing improves cardiac function in rats with heart failure through activation of the cAMP signaling pathway. *Journal of cellular physiology* (2019) 234(8):13169-81. doi: 10.1002/jcp.27987.
9. Bauters C, Kumarswamy R, Holzmann A, Bretthauer J, Anker SD, Pinet F, et al. Circulating miR-133a and miR-423-5p fail as biomarkers for left ventricular remodeling after myocardial infarction. *BMC cardiovascular disorders* (2013) 168(3):1837-40. Epub 2017/07/02. doi: 10.1186/s12872-017-0609-z
10.1016/j.ijcard.2012.12.074. PubMed PMID: 23347612; PubMed Central PMCID: PMCPMC5493858 All authors consent to publish. COMPETING INTERESTS: The authors declare that they have no competing interests. PUBLISHER'S NOTE: Springer Nature remains neutral with regard to jurisdictional claims in published maps and institutional affiliations.
10. Bayés-Genis A, Lanfear DE, de Ronde MWJ, Lupón J, Leenders JJ, Liu Z, et al. Prognostic value of circulating microRNAs on heart failure-related morbidity and mortality in two large diverse cohorts of general heart failure patients. *European journal of heart failure* (2018) 20(1):67-75. Epub 2017/09/28. doi: 10.1002/ejhf.984. PubMed PMID: 28949058.
11. Beaumont J, López B, Ravassa S, Hermida N, José GS, Gallego I, et al. MicroRNA-19b is a potential biomarker of increased myocardial collagen cross-linking in patients with aortic stenosis and heart failure. *Scientific reports* (2017) 7:40696. doi: 10.1038/srep40696.
12. Cai WF, Liu GS, Lam CK, Florea S, Qian J, Zhao W, et al. Up-regulation of micro-RNA765 in human failing hearts is associated with post-transcriptional regulation of protein phosphatase

inhibitor-1 and depressed contractility. *European journal of heart failure* (2015) 17(8):782-93. Epub 2015/07/17. doi: 10.1002/ejhf.323. PubMed PMID: 26177627; PubMed Central PMCID: PMCPmc5693221.
13. Chen F, Yang J, Li Y, Wang H. Circulating microRNAs as novel biomarkers for heart failure. *Hellenic journal of cardiology : HJC = Hellenike kardiologike epitheorese* (2018) 59(4):209-14. Epub 2017/11/12. doi: 10.1016/j.hjc.2017.10.002. PubMed PMID: 29126951.
14. Dawson K, Wakili R, Ordög B, Clauss S, Chen Y, Iwasaki Y, et al. MicroRNA29: a mechanistic contributor and potential biomarker in atrial fibrillation. *Circulation* (2013) 127(14):1466-75, 75e1-28. Epub 2013/03/06. doi: 10.1161/circulationaha.112.001207. PubMed PMID: 23459615.
15. Dos Reis Schneider SI, Silvello D, Martinelli NC, Garbin A, Biolo A, Clausell N, et al. Plasma levels of microRNA-21,-126 and-423-5p alter During clinical improvement and are associated with the prognosis of acute heart failure. *Molecular medicine reports* (2018) 17(3):4736-46. doi: 10.3892/mmr.2018.8428.
16. Duan Q, Yang L, Gong W, chaugai S, Wang F, Chen C, et al. MicroRNA-214 Is Upregulated in Heart Failure Patients and Suppresses XBP1-Mediated Endothelial Cells Angiogenesis. *Journal of cellular physiology* (2015) 230(8):1964-73. doi: 10.1002/jcp.24942.
17. Funahashi H, Izawa H, Hirashiki A, Cheng XW, Inden Y, Nomura M, et al. Altered microRNA expression associated with reduced catecholamine sensitivity in patients with chronic heart failure. *Journal of cardiology* (2011) 57(3):338-44. doi: 10.1016/j.jjcc.2011.01.009.
18. Goldraich LA, Martinelli NC, Matte U, Cohen C, Andrades M, Pimentel M, et al. Transcoronary gradient of plasma microRNA 423-5p in heart failure: Evidence of altered myocardial expression. *Biomarkers : biochemical indicators of exposure, response, and susceptibility to chemicals* (2014) 19(2):135-41. doi: 10.3109/1354750X.2013.870605.
19. Gupta MK, Halley C, Duan ZH, Lappe J, Viterna J, Jana S, et al. miRNA-548c: a specific signature in circulating PBMCs from dilated cardiomyopathy patients. *Journal of molecular and cellular cardiology* (2013) 62:131-41. Epub 2013/06/06. doi: 10.1016/j.yjmcc.2013.05.011. PubMed PMID: 23735785; PubMed Central PMCID: PMCPmc3735826.
20. He Y, Feng Z, Lu J, Wang R, Huang C, Zhou Y. Exploring biomarkers and therapeutic targets for pressure overload induced heart failure based on microarray data. *Cardiovascular diagnosis and therapy* (2020) 10(5):1226-37. doi: 10.21037/cdt-20-465.
21. Hu L, Xu YN, Wang Q, Liu MJ, Zhang P, Zhao LT, et al. Aerobic exercise improves cardiac function in rats with chronic heart failure through inhibition of the long non-coding RNA metastasis-associated lung adenocarcinoma transcript 1 (MALAT1). *Annals of translational medicine* (2021) 9(4). doi: 10.21037/atm-20-8250.
22. Dubois-Deruy E, Cuvelliez M, Fiedler J, Charrier H, Mulder P, Hebbar E, et al. MicroRNAs regulating superoxide dismutase 2 are new circulating biomarkers of heart failure. (2017) 7(1):14747. doi: 10.1038/s41598-017-15011-6. PubMed PMID: 29116107.
